# Machine Learning for Predicting Delayed Graft Function and Graft Survival After Kidney Transplantation: A Systematic Review and Meta-Analysis

**DOI:** 10.3390/jcm15218535

**Published:** 2026-11-02

**Authors:** Shekhar Gogna, Piyush Gupta, David Walls, Reginald Gohh

**Affiliations:** MedStar Georgetown Transplant Institute, Department of Surgery, MedStar Georgetown University Hospital, Georgetown University School of Medicine, 3800 Reservoir Road NW, Washington, DC 20007, USA; piyushesama@gmail.com (P.G.); david.walls@medstar.net (D.W.); reginald.y.gohh@medstar.net (R.G.)

**Keywords:** kidney transplantation, delayed graft function, graft survival, machine learning, prediction model, external validation, calibration, PROBAST

## Abstract

**Background/Objectives:** Machine-learning (ML) models are promoted for predicting delayed graft function (DGF) and graft survival after kidney transplantation; we examined whether they outperform regression and how well either has been validated. **Methods:** We searched PubMed/MEDLINE, Embase, and Web of Science through 28 June 2026, searched citations for named DGF models, extracted data in duplicate, assessed risk of bias using the Prediction Model Risk of Bias ASsessment Tool (PROBAST), and pooled only external validations of the same published model. **Results:** We included 217 reports (145 on DGF and 91 on graft survival, 19 on both); across seven external cohorts, the Irish 2010 DGF model had a pooled area under the curve (AUC) of 0.73 (95% confidence interval 0.69–0.77), the median difference in AUC or C-statistic between ML and regression in 28 same-cohort comparisons was 0.01 (interquartile range −0.02 to 0.03), and 208 of the 212 non-pediatric reports were at high risk of bias. **Conclusions:** ML has not been shown to predict DGF or graft survival better than regression, and the comparisons are too heterogeneous to show that it is worse; a model should inform organ acceptance or recipient counseling only after it has shown discrimination and calibration in an independent cohort.

## 1. Introduction

Kidney transplantation is the preferred treatment for end-stage kidney disease, yet delayed graft function (DGF) and long-term graft survival remain difficult to predict. DGF, usually defined as dialysis within the first week after transplantation, occurs in 21–31% of extended-criteria donor transplants and is associated with higher risks of graft loss and acute rejection [1,2]. Reliable prediction could support donor selection, recipient counseling, and early postoperative care.

Over the past decade, many machine-learning (ML) models have been developed to predict DGF and graft survival [3]. They include survival forests, gradient boosting, and deep-learning models trained on registry and single-center data [4,5,6,7]. Such models are expected to capture relationships that regression misses, but this advantage has not been shown consistently in clinical prediction research [8].

The evidence is hard to interpret. Many studies report no confidence interval, no calibration, and no performance in an independent population, so a model can look accurate in its own study and perform poorly elsewhere [9]. The Prediction model Risk Of Bias ASsessment Tool (PROBAST) is the recommended tool for assessing risk of bias in prediction-model studies, and TRIPOD+AI (Transparent Reporting of a multivariable prediction model for Individual Prognosis Or Diagnosis, extended to artificial intelligence) sets the reporting standard for models built with regression or ML [10,11]. Neither has been applied consistently in transplantation.

Previous reviews have examined graft survival or DGF separately [12,13]. Studies that compare ML with regression also differ in outcome, metric, predictors, and validation design, which limits what a summary of them can show.

We systematically reviewed regression and ML models that predict DGF or graft survival after kidney transplantation. We aimed to describe their discrimination, calibration, validation, and risk of bias, to pool external validations of models that have been tested in several independent cohorts, and to compare ML with regression where both were evaluated in the same patients. The review follows the Preferred Reporting Items for Systematic Reviews and Meta-Analyses (PRISMA) 2020 statement [14]. The PRISMA 2020 checklist is provided in Supplementary File S1.

## 2. Materials and Methods

### 2.1. Protocol

We followed a protocol based on the CHARMS checklist (Checklist for critical Appraisal and data extraction for systematic Reviews of prediction Modeling Studies) that was written before screening but not prospectively registered [15]. Three amendments to the protocol are reported here. First, the protocol limited inclusion to adults; pediatric studies are instead summarized separately and do not enter any other summary. Second, discrimination was pooled only across external validations of the same named model, and not across unrelated models, because only the former estimate a common quantity [16]. Third, the protocol required a reported discrimination statistic for inclusion; prediction studies that reported none were excluded, so that reporting in this field could be described (Section 2.2). A named model is a published model or score that later studies identify by name and apply without refitting.

### 2.2. Eligibility Criteria

We included original studies in kidney transplantation that developed, validated, or updated a multivariable model, score, or ML algorithm, or that evaluated the discrimination of a single marker for predicting DGF or graft failure. A prediction study remained eligible when it reported no area under the receiver operating characteristic curve (AUC) or C-statistic. We excluded analyses that reported only associations (odds or hazard ratios), studies of other organs or other outcomes, reviews, editorials, conference abstracts, and animal studies. Gray literature and preprint servers were not searched.

DGF was accepted as each study defined it, and the definition was recorded. Recipient-level occurrence of DGF was analyzed separately from DGF duration, DGF severity, donor-level endpoints, and composites of delayed and slow graft function. For graft survival, we accepted graft failure as each study defined it, recorded whether death was censored, and recorded the metric, the prediction horizon, and the time at which the prediction was made. Composite outcomes that combined graft failure with a decline in graft function or with other events were not accepted.

### 2.3. Information Sources and Search

We searched PubMed/MEDLINE, Embase (Ovid) and the Web of Science Core Collection from inception to 28 June 2026, with separate strategies for DGF and graft survival. Scopus and Semantic Scholar were searched through the Consensus interface. To find external validations that keyword searching could miss, we screened all records citing five named DGF models (Irish 2003, Irish 2010, Jeldres 2009, Chapal 2014 and Zaza 2015) in Europe PMC [17,18,19,20,21]. Search strategies, limits, and yields are given in Supplementary Table S1.

### 2.4. Study Selection

Two reviewers (P.G. and S.G.) independently screened titles and abstracts and resolved disagreements by consensus (DGF search: 380 records, 92.9% agreement, κ = 0.85; graft-survival search: 100 records, 97% agreement, κ = 0.94). Records retrieved by the DGF search were also screened against the graft-survival criteria, and every report assessed in full text was assessed for both outcomes. Because a prediction study was eligible without an AUC, every record whose abstract described a model or marker was carried to full-text assessment even when the abstract gave no discrimination statistic. Both reviewers assessed the full texts. The full text of one report could not be obtained; it was assessed from the published abstract, which gave the population, the outcome, and the model, and is marked with a dagger in the supplementary tables. Reports excluded at full text are listed with reasons in Supplementary Table S7.

### 2.5. Data Extraction

Two reviewers independently extracted data from the full text of each included report (from the abstract for the one report without a full text) with a prespecified form, and each extracted estimate was checked against the passage or table that reported it. Disagreements were resolved by consensus after a further reading of the report. We extracted the population, setting and sample size; the outcome definition and number of events; each model, its class and its predictors; the time at which predictions were made; every reported AUC or C-statistic with its confidence interval and evaluation design; calibration; handling of missing data; and internal and external validation. Evaluation design was classified as apparent (the data used to develop the model), internal (random split, cross-validation, or bootstrap), temporal or geographic (a later period or held-out centers from the same source), or external (an independent cohort). The prediction time for graft survival was classified as before or at transplantation, from a posttransplant landmark (a fixed time after transplantation at which prediction starts), or dynamic (updated during follow-up). The machine-assisted extraction was independently reviewed by P.G. and S.G.

One estimate per report and outcome was taken for the descriptive summaries. We used the model that the authors presented as final, in the most independent evaluation they reported. When several estimates remained at that level, for example, a marker measured at several times or several existing scores validated in one report, we took their median and not the highest value. For graft survival we used the overall C-statistic and, where only horizon-specific values were given, the horizon closest to five years. Reports whose first authors share a surname and a year of publication are distinguished by a letter after the year, in order of PubMed identifier. Numerical intervals for individual graft-survival estimates are provided in Supplementary Table S4.

### 2.6. Risk-of-Bias Assessment

Both reviewers applied PROBAST to every included report, answering its 20 signaling questions across the participant, predictor, outcome, and analysis domains [10]. A domain was rated at high risk when any question was answered no or probably no, at low risk when all were answered yes or probably yes, and unclear otherwise; the overall rating followed the same rule across domains. Absence of a calibration assessment counted against the analysis domain; absence of a confidence interval or of external validation did not. For reports of a single marker, the questions on calibration, univariable selection and model weights were not applicable. For the one report assessed from the abstract, a question was answered no only when the abstract stated the fact, and otherwise as no information. P.G. and S.G. independently reviewed all PROBAST assessments.

### 2.7. Synthesis and Statistical Analysis

Validations of the five named DGF models were sought by citation searching. Each model was meta-analyzed when at least three independent cohorts had externally validated it for the occurrence of DGF and had reported a 95% confidence interval. AUCs were pooled on the logit scale with a DerSimonian–Laird estimate of between-study variance and the Hartung–Knapp–Sidik–Jonkman adjustment, applied so that the adjusted interval was never narrower than the conventional one [22,23]. We report a 95% confidence interval (CI), a 95% prediction interval, I^2,^ and τ^2^. In sensitivity analyses, we added cohorts that gave no interval, deriving the standard error from the reported numbers of events with the Hanley–McNeil method, and, where at least four cohorts were pooled, we omitted each cohort in turn [24]. Where a report gave the same model in several samples, we used the sample to which the model’s own eligibility criteria had been applied and otherwise the largest sample (Supplementary Table S3).

All other estimates were summarized as medians with interquartile ranges (IQR), by outcome, model class, evaluation design and setting. They were not pooled. Because pooling was limited to named models with few cohorts each, we did not assess reporting bias statistically or grade the certainty of the evidence; risk of bias is reported for every report instead. A funnel plot is shown for the Irish 2010 model, the only named model validated in ten or more cohorts. Its asymmetry was not tested, because the standard error of a logit-transformed AUC depends on the AUC itself and 7 of the 14 standard errors were derived from numbers of events.

Where a report evaluated ML and regression in the same cohort, we calculated the difference in AUC or C-statistic (ML minus regression). The comparison used models built on the same predictors when the report offered them, and the most independent evaluation in which both classes were assessed. When several predictor sets qualified, we used the one that contained the authors’ final model; when no set was shared, each class was represented by its models on its largest predictor set. When several comparisons still qualified, for example, in separate eras, we took the median difference. For graft survival, the horizon was chosen as for the descriptive summaries. The regression comparator was a model fitted in the study cohort; a published regression model applied unchanged was used only when the report fitted none. Penalized regression was counted as ML when the report presented it as an ML algorithm and as regression otherwise. ML models were averaged within family (tree ensembles, support-vector machines, neural networks and others). To avoid selecting the most favorable result, the main comparison used the mean of the family averages; the best family is shown for reference. The differences are also shown for each ML family separately; because the families were evaluated in different comparisons, they are not compared with one another. We describe these differences and the features in which the comparisons differ. We did not pool or test them, because they do not estimate a common quantity and the uncertainty of a paired difference was rarely reported. Analyses were performed in Python 3.14 with NumPy and SciPy.

## 3. Results

### 3.1. Study Selection

**Figure 1:**
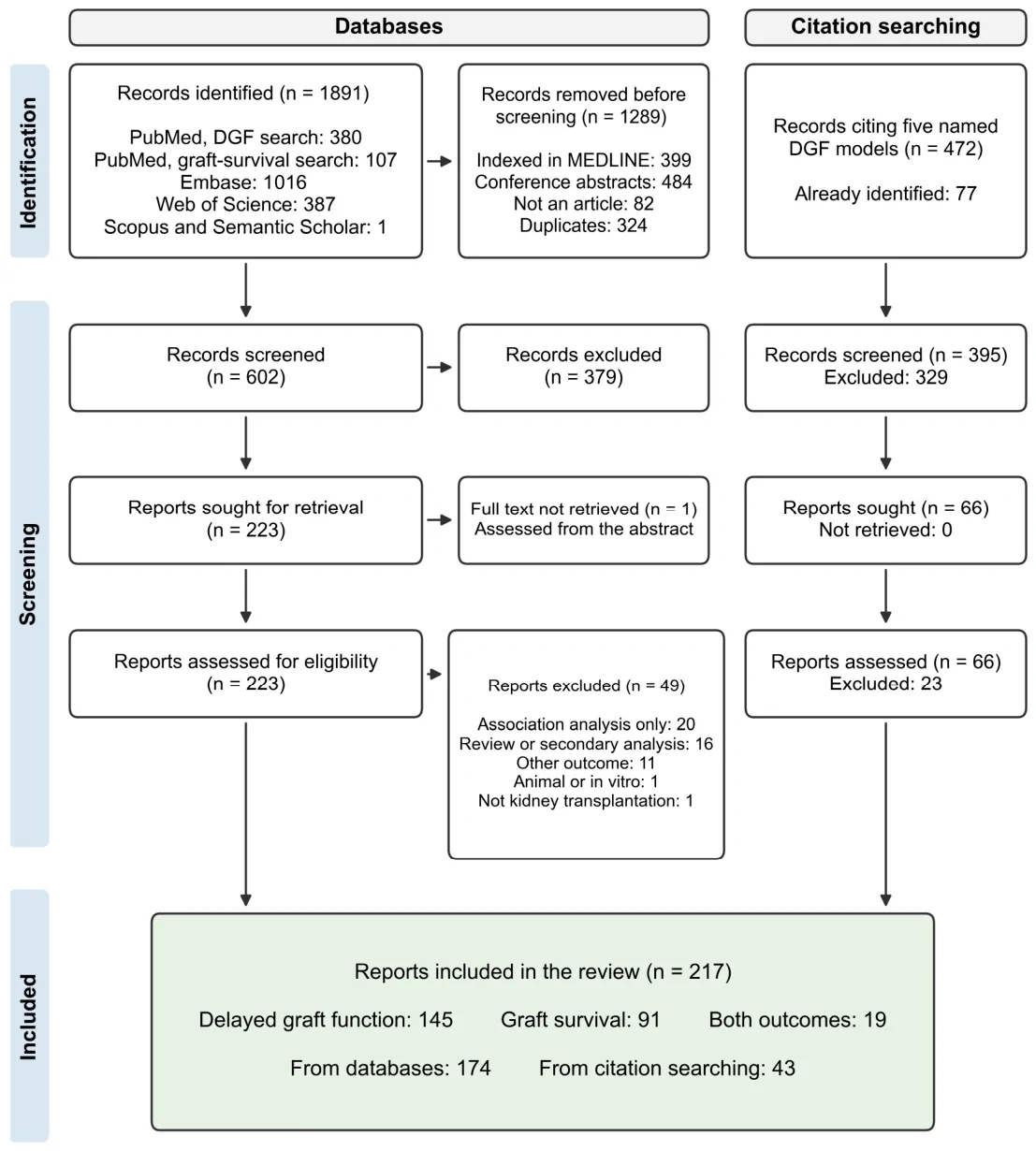
PRISMA 2020 flow diagram. The left column shows records from the database searches and the right column shows records from citation searching of five named DGF models. Reports assessed in full text were assessed for both outcomes. One report whose full text could not be obtained was assessed from the abstract. DGF, delayed graft function.

The database searches identified 1891 records (Figure 1). Before screening, we removed 1289: 399 Embase records indexed in MEDLINE, 484 conference abstracts, 82 records that were not articles, and 324 duplicates. We screened 602 records and excluded 379 at title and abstract. Of 223 reports sought, 222 were assessed in full text and 1 was assessed based on its abstract; 49 were excluded, most often because they analyzed associations without evaluating a prediction model or marker (20), were reviews or secondary analyses or gave no original data (16), or their outcome was not DGF or graft failure (11). Citation searching identified 472 records, of which 395 were new; 66 reports were assessed in full text, and 43 were included.

In total, 217 reports were included: 145 addressed DGF and 91 graft survival, 19 of them both (Table 1). Five reports were restricted to children and are summarized separately in Section 3.5. The others, called non-pediatric below, comprise 139 reports in adults, 10 in mixed-age cohorts and 63 that did not state an age range.

### 3.2. Prediction of Delayed Graft Function

**Figure 2:**
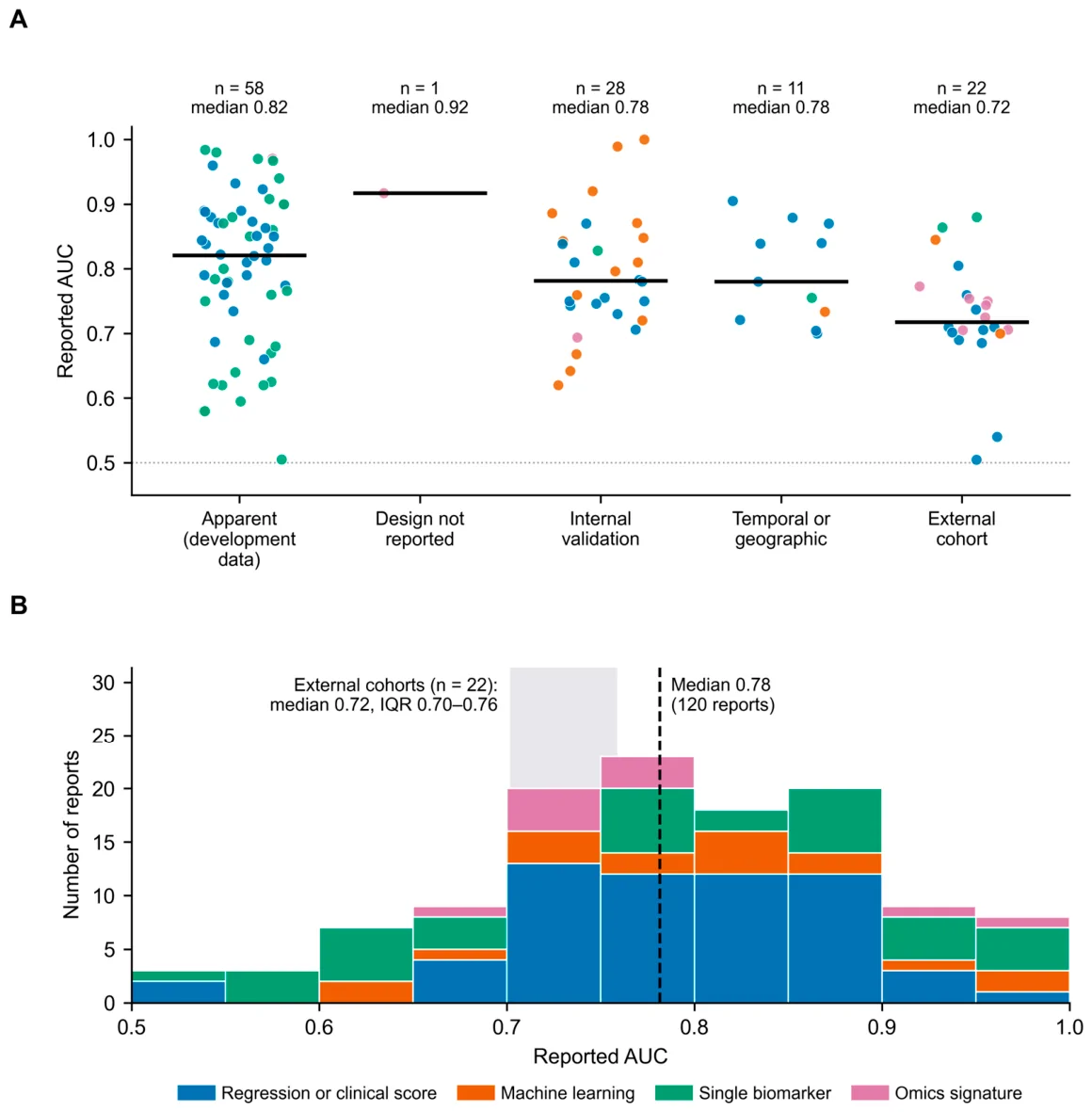
Reported discrimination of models for the occurrence of delayed graft function, one estimate per report. (**A**) By evaluation design; each point is one report, and horizontal bars are medians. (**B**) Distribution by model class; the dashed line is the median of all reports, and the shaded band is the interquartile range of the reports evaluated in an external cohort. Colors show model class in both panels. Values are descriptive and were not pooled. AUC, area under the receiver operating characteristic curve; IQR, interquartile range.

Of the 142 non-pediatric DGF reports, 120 reported discrimination for the occurrence of DGF in recipients (Figure 2A), 15 for another target (DGF duration or severity, a donor-level endpoint, or delayed and slow graft function combined), and 7 reported no AUC. DGF was defined as dialysis within seven days in 100 reports, by another definition in 32, and was not defined in 10. The median cohort had 201 recipients and 44 with DGF; 73 of the 94 reports that gave the number of events had fewer than 100.

The median reported AUC was 0.78 (IQR 0.71–0.87), with a range of 0.50–1.00 (Table 2, Figure 2A). It was 0.82 in the 58 reports that evaluated the model only in its development data, 0.78 in 28 with internal validation, 0.78 in 11 with temporal or geographic validation, and 0.72 in the 22 with an external cohort. Single-center reports gave higher values than registry or multicenter reports (median 0.82 versus 0.73). Median AUCs were 0.79 for regression models and clinical scores, 0.81 for ML models, 0.78 for single biomarkers, and 0.75 for omics signatures. A 95% confidence interval accompanied 60 of the 120 estimates. In the 27 reports that gave the AUC of the final model both in its development data and in a validation sample, the development value was higher by a median of 0.01 (higher in 17); in the 12 with an external cohort, the median was 0.02.

**Figure 3:**
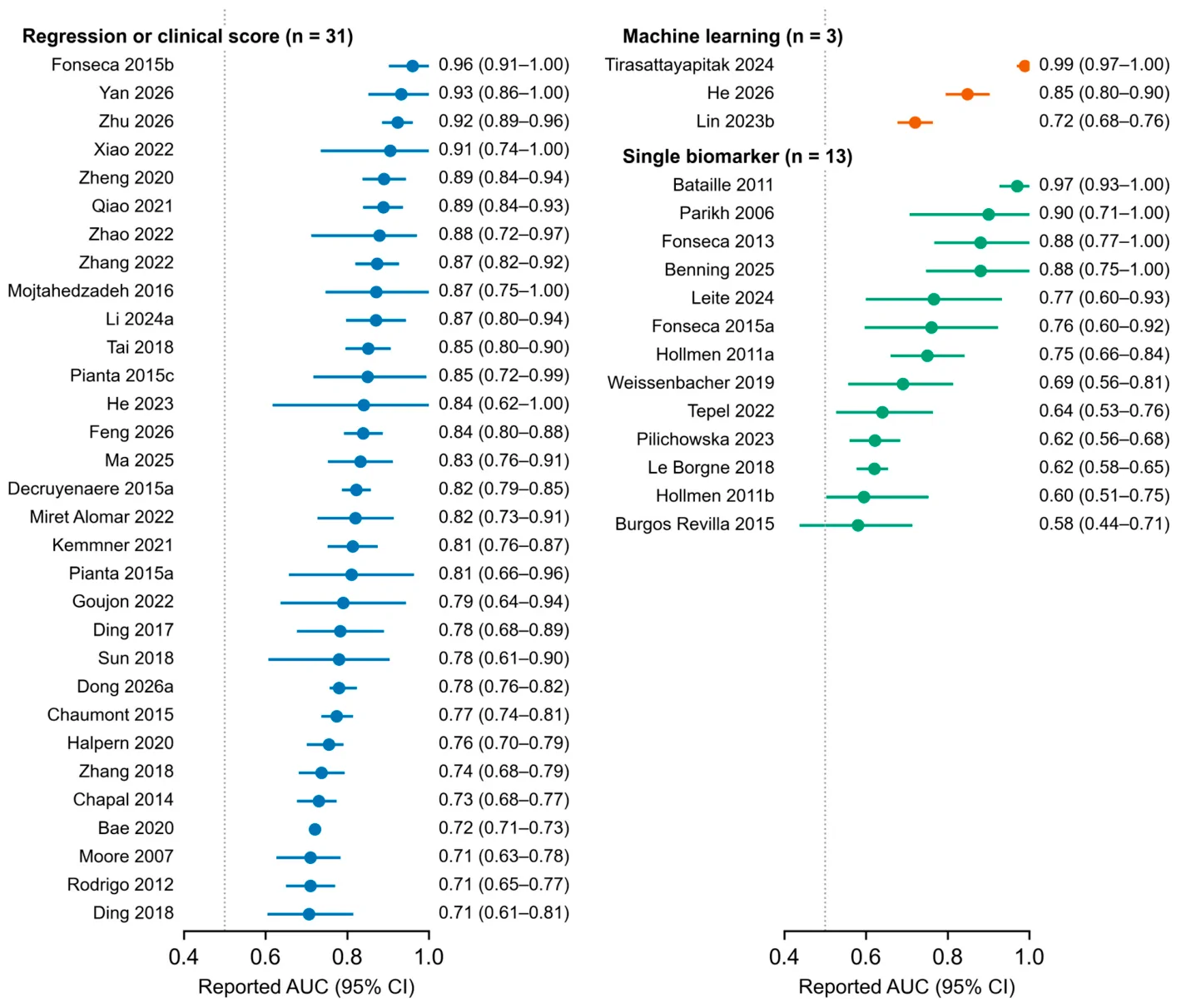
Reported AUCs with 95% confidence intervals for the occurrence of delayed graft function, in the 47 reports whose estimates carried their own intervals (Supplementary Table S2) [20,25,26,27,28,29,30,31,32,33,34,35,36,37,38,39]. The dotted line marks an AUC of 0.50. No pooled or summary value is shown, because the models differ. AUC, area under the receiver operating characteristic curve; CI, confidence interval.

Figure 2B shows the distribution of the 120 estimates by model class: 81 lay between 0.70 and 0.90, and the 22 from external cohorts had a median of 0.72 (IQR 0.70–0.76). Figure 3 shows the 47 estimates that carried their own 95% confidence interval; in 20 of them the lower limit was below 0.70.

#### External Validation of Named Models

**Figure 4:**
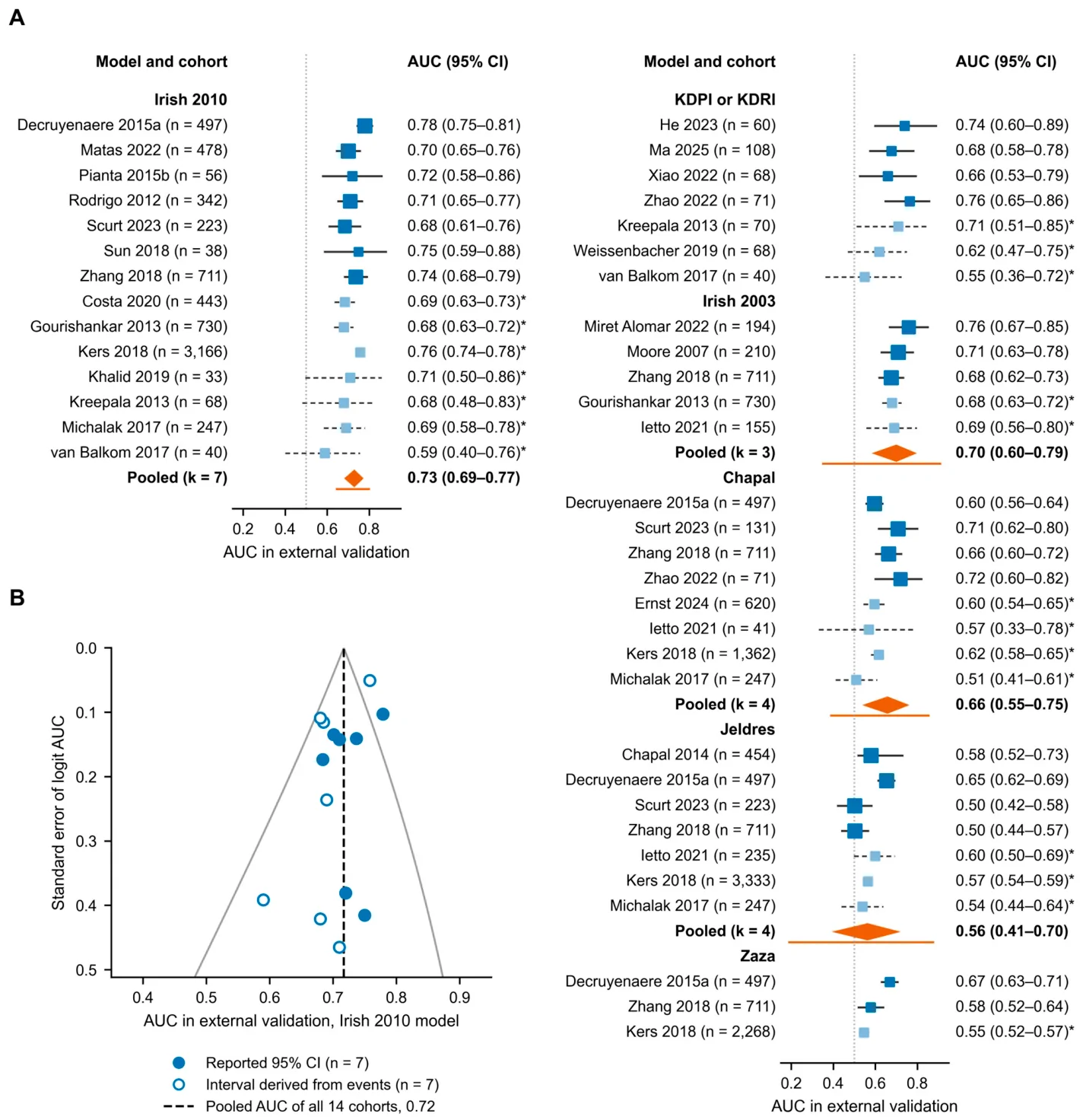
External validation of named models for delayed graft function [17,18,19,20,21,25,26,27,28,29,30,31,32,33,34,35,36,40,41,42,43,44,45,46,47,48,49,50,52]. (**A**) Cohort estimates (squares) with 95% confidence intervals; dashed intervals, marked with an asterisk, were derived from the reported numbers of events. Diamonds are random-effects pooled estimates of the k cohorts that reported an interval, and the line beneath each diamond is the 95% prediction interval. (**B**) Funnel plot of the 14 cohorts that validated the Irish 2010 model. The dashed line is the pooled AUC of all 14 cohorts (0.72), and the curves mark the 95% limits expected around it in the absence of heterogeneity; for open circles, the standard error was derived from the reported numbers of events. Dark blue indicates estimates with reported 95% confidence intervals, light blue indicates estimates with derived intervals, and orange diamonds indicate pooled estimates. AUC, area under the receiver operating characteristic curve; CI, confidence interval; KDPI, Kidney Donor Profile Index; KDRI, Kidney Donor Risk Index.

Fourteen independent cohorts externally validated the Irish 2010 model [25,26,27,28,29,40,41,42,43,44,45,46,47,48]. Seven reported a 95% confidence interval, and their pooled AUC was 0.73 (95% CI 0.69–0.77), with a 95% prediction interval of 0.65–0.80 (Figure 4A, Table 3). Heterogeneity between cohorts was moderate (Q = 9.84, df = 6, *p* = 0.13; I^2^ = 39%). Omitting each cohort in turn gave pooled AUCs of 0.71–0.74. Adding the seven cohorts without a reported interval gave 0.72 (95% CI 0.69–0.74; I^2^ = 57%). Figure 4B plots the AUC of each of the 14 cohorts against its standard error. Nine lay below the pooled value of all fourteen cohorts, and the less precise half did not report higher AUCs than the more precise half (median 0.69 against 0.71). Asymmetry was not tested (Section 2.7).

The Kidney Donor Profile Index (KDPI) or its underlying Kidney Donor Risk Index (KDRI), which were designed to predict graft failure and not DGF [49], were tested as predictors of DGF in seven cohorts, with AUCs of 0.55–0.76 [30,31,32,33,34,46,48]. The Irish 2003 nomogram had AUCs of 0.68–0.76 in five cohorts [29,35,36,43,50]; the three that reported an interval gave a pooled AUC of 0.70 (95% CI 0.60–0.79). A sixth report applied this nomogram to 169 recipients at one United States center and gave no AUC; predicted risk did not differ significantly between recipients with and without DGF (*p* = 0.07), and the mean predicted risk was 41% against an observed incidence of 25% [51]. The Chapal score had AUCs of 0.51–0.72 in eight cohorts [25,29,33,41,44,47,50,52]; the four that reported an interval gave a pooled AUC of 0.66 (95% CI 0.55–0.75). The Jeldres nomogram had AUCs of 0.50–0.65 in seven cohorts [20,25,29,41,44,47,50]; the four that reported an interval gave a pooled AUC of 0.56 (95% CI 0.41–0.70). The Zaza model had AUCs of 0.55–0.67 in three cohorts [25,29,44]. Where the calibration of the Irish 2003, Chapal, Jeldres and Zaza scores was tested with the Hosmer–Lemeshow statistic, the fit was rejected: for all four in the Chinese cohort (*p* < 0.05 each) [29], for the Jeldres, Chapal and Zaza scores in one Belgian cohort (*p* < 0.001 each) [25] and for the Jeldres and Chapal scores in another (*p* < 0.05 and *p* = 0.02) [47]. In the Dutch registry, where calibration was assessed with calibration plots, the Chapal score was not well calibrated [44]. Heterogeneity was substantial for the Chapal score and Jeldres nomogram (I^2^ = 65% and 86%); the pooled value for the Irish 2003 nomogram rests on three cohorts, so its prediction interval is wide although heterogeneity was low. Prediction intervals are given in Table 3, and none of these pooled values can be interpreted on its own. No other named score had a higher pooled AUC than the Irish 2010 model.

Calibration of the Irish 2010 model was assessed in 5 of the 14 reports that validated it. It was adequate in four, by the Hosmer–Lemeshow test in three (*p* = 0.31 to 0.89) [27,29,47] and with a calibration slope of 1.00 in the Dutch registry [44], and the model overestimated risk in one cohort (*p* = 0.018) [25]. A sixth report found that the model overestimated risk in pump-perfused kidneys, a group excluded when the model was developed [40]. In the Chinese cohort, where calibration was adequate overall, observed DGF was 27% against 9% predicted in kidneys from donors aged five years or younger [29].

**Table 3:** External validations of named prediction models.

Model and Validation Cohort	Recipients (Events)	AUC or C-Statistic (95% CI)
**Irish 2010, for delayed graft function**		
Decruyenaere 2015 [25]	497 (62)	0.78 (0.75–0.81)
Matas 2022 [40]	478 (116)	0.70 (0.65–0.76)
Pianta 2015b [26]	56 (22)	0.72 (0.58–0.86)
Rodrigo 2012 [27]	342	0.71 (0.65–0.77)
Scurt 2023 [41]	223 (109)	0.68 (0.61–0.76)
Sun 2018 [28]	38 (5)	0.75 (0.59–0.88)
Zhang 2018 [29]	711 (125)	0.74 (0.68–0.79)
Costa 2020 [42]	443 (235)	0.69 (not reported)
Gourishankar 2013 [43]	730 (189)	0.68 (not reported)
Kers 2018 [44]	3166 (1148)	0.76 (not reported)
Khalid 2019 [45]	33 (13)	0.71 (not reported)
Kreepala 2013 [46]	68 (12)	0.68 (not reported)
Michalak 2017 [47]	247 (38)	0.69 (not reported)
van Balkom 2017 [48]	40 (27)	0.59 (not reported)
Pooled, cohorts with a reported interval (k = 7)		0.73 (0.69–0.77); PI 0.65–0.80
**KDPI or KDRI, for delayed graft function**		
He 2023 [30]	60 (21)	0.74 (0.60–0.89)
Ma 2025 [31]	108 (48)	0.68 (0.58–0.78)
Xiao 2022 [32]	68 (45)	0.66 (0.53–0.79)
Zhao 2022 [33]	71 (23)	0.76 (0.65–0.86)
Kreepala 2013 [46]	70 (12)	0.71 (not reported)
Weissenbacher 2019 [34]	68 (23)	0.62 (not reported)
van Balkom 2017 [48]	40 (27)	0.55 (not reported)
**Irish 2003, for delayed graft function**		
Miret Alomar 2022 [35]	194 (43)	0.76 (0.67–0.85)
Moore 2007 [36]	210 (75)	0.71 (0.63–0.78)
Zhang 2018 [29]	711 (125)	0.68 (0.62–0.73)
Gourishankar 2013 [43]	730 (189)	0.68 (not reported)
Ietto 2021 [50]	155 (25)	0.69 (not reported)
Pooled, cohorts with a reported interval (k = 3)		0.70 (0.60–0.79); PI 0.35–0.91
**Chapal, for delayed graft function**		
Decruyenaere 2015a [25]	497 (62)	0.60 (0.56–0.64)
Scurt 2023 [41]	131	0.71 (0.62–0.80)
Zhang 2018 [29]	711 (125)	0.66 (0.60–0.72)
Zhao 2022 [33]	71 (23)	0.72 (0.60–0.82)
Ernst 2024 [52]	620 (166)	0.60 (not reported)
Ietto 2021 [50]	41 (7)	0.57 (not reported)
Kers 2018 [44]	1362 (292)	0.62 (not reported)
Michalak 2017 [47]	247 (38)	0.51 (not reported)
Pooled, cohorts with a reported interval (k = 4)		0.66 (0.55–0.75); PI 0.39–0.85
**Jeldres, for delayed graft function**		
Chapal 2014 [20]	454	0.58 (0.52–0.73)
Decruyenaere 2015a [25]	497 (62)	0.65 (0.62–0.69)
Scurt 2023 [41]	223 (109)	0.50 (0.42–0.58)
Zhang 2018 [29]	711 (125)	0.50 (0.44–0.57)
Ietto 2021 [50]	235 (41)	0.60 (not reported)
Kers 2018 [44]	3333 (1183)	0.57 (not reported)
Michalak 2017 [47]	247 (38)	0.54 (not reported)
Pooled, cohorts with a reported interval (k = 4)		0.56 (0.41–0.70); PI 0.19–0.88
**Zaza, for delayed graft function**		
Decruyenaere 2015a [25]	497 (62)	0.67 (0.63–0.71)
Zhang 2018 [29]	711 (125)	0.58 (0.52–0.64)
Kers 2018 [44]	2268 (801)	0.55 (not reported)

Each row is one cohort; where a report gave a model in several samples, the sample taken is named in Supplementary Table S3. Pooled estimates use a random-effects model with the Hartung–Knapp–Sidik–Jonkman adjustment. Every pooled cohort defined DGF as dialysis in the first week, except **Sun 2018 [28]**, which also counted a fall in serum creatinine of less than 10% in the first 48 h. For **Zhang 2018 [29]**, the values of four models are those printed in a figure in the report, whose text describes them as below 0.6; the number of recipients is that of the whole cohort. KDPI and KDRI were not among the five models whose validations were sought by citation searching, so their cohorts are listed and not pooled. For **Miret Alomar 2022 [35]**, the **Irish 2003 score [17]** was not calculated for pre-emptive recipients (13 of 202), and the number evaluated is not stated. PI, 95% prediction interval; CI, confidence interval; KDPI, Kidney Donor Profile Index; KDRI, Kidney Donor Risk Index.

### 3.3. Prediction of Graft Survival

**Figure 5:**
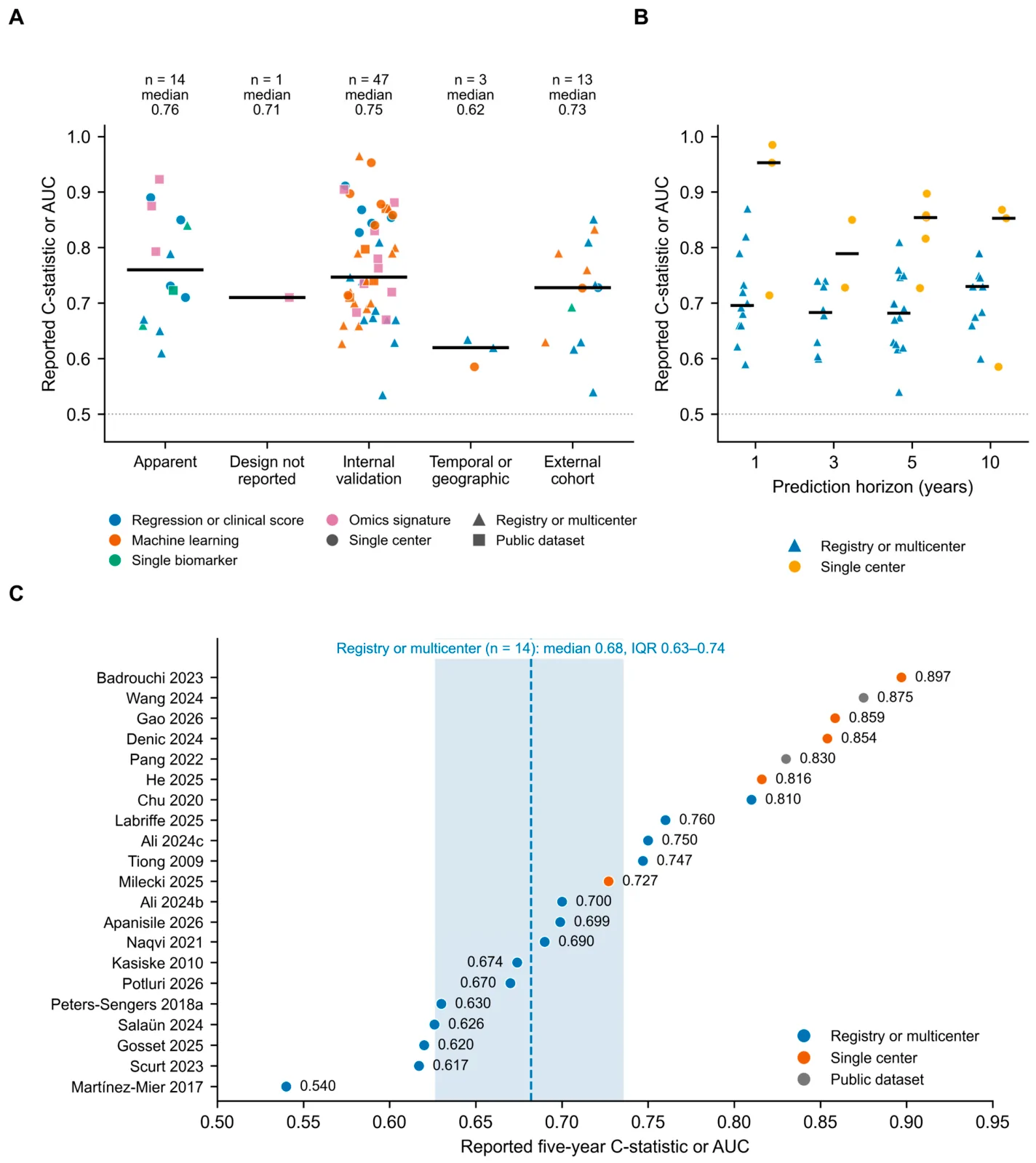
Reported discrimination of graft-survival models. (**A**) By evaluation design; colors show model class and symbols the setting. (**B**) By prediction horizon, in the registry or multicenter and single-center reports that gave horizon-specific values; reports from public datasets are not shown. In (**A**,**B**), each point is one report and horizontal bars are medians. (**C**) Five-year values in the 21 reports that gave one, by setting [4,5,6,7,41,53,54,55,56,57,58,59,60,61,62,63,64,65,66,67,68]: the final model of each report, and the median where it was given several five-year values (Supplementary Table S4). The dashed line and shaded band are the median and interquartile range of the registry or multicenter reports. Values are descriptive and were not pooled. AUC, area under the receiver operating characteristic curve; IQR, interquartile range.

Of the 89 non-pediatric graft-survival reports, 78 reported a C-statistic or AUC (Figure 5). The outcome was death-censored graft failure in 43 reports, all-cause graft loss in 13, and another definition of graft loss (early or cause-specific) in 4; 29 did not state whether death was censored. Predictions were made before or at transplantation in 36 reports, from a posttransplant landmark in 32, and dynamically in 8; 13 did not state the prediction time.

The median reported discrimination was 0.74 (IQR 0.67–0.84), with a range of 0.54–0.97 (Table 2, Figure 5A). Registry and multicenter reports gave lower values than single-center reports (median 0.69 versus 0.85). Models that predicted before or at transplantation had a median of 0.69, and models that predicted from a posttransplant landmark had a median of 0.81; the two answer different clinical questions and are not directly comparable. In registry and multicenter reports, median values were 0.70 at one year, 0.68 at five years, and 0.73 at ten years (Figure 5B). Figure 5C shows the 21 reports that gave a five-year value; the five single-center reports had a median of 0.85. Source-reported uncertainty for individual graft-survival estimates is detailed in Supplementary Table S4. In the 23 reports that gave the final model in its development data and in a validation sample, the development value was higher by a median of 0.05 (higher in 15); the validation was internal in 20 of them. Among the included reports, no graft-survival model had three external validations with a confidence interval, so none was pooled.

### 3.4. Machine Learning Compared with Regression in the Same Cohort

**Figure 6:**
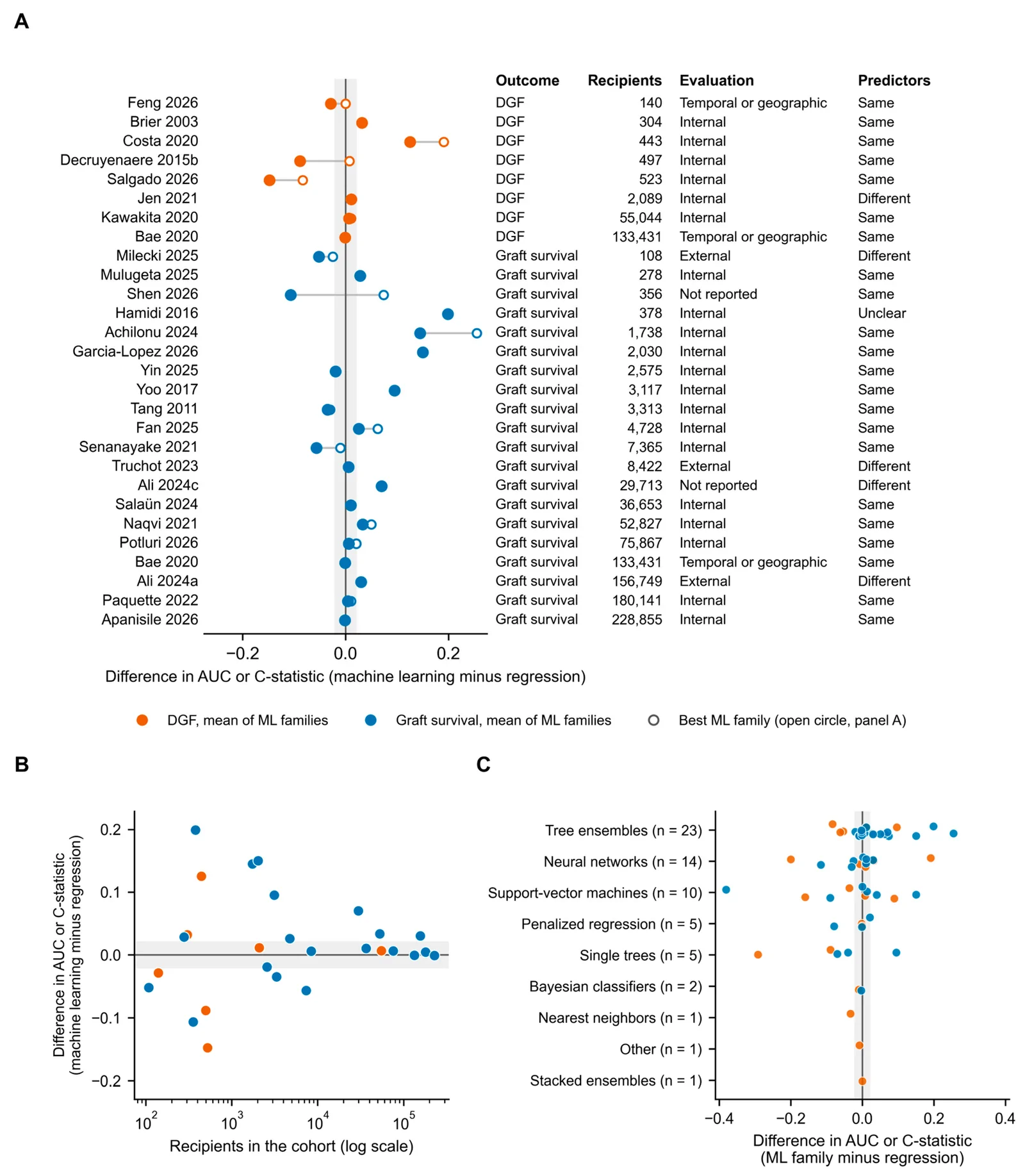
Difference in discrimination between machine-learning (ML) and regression models evaluated in the same cohort [3,4,5,37,38,39,42,53,54,55,56,57,58,59,60,61,62,63,64,65,66,67,68,69,70,71,72]. (**A**) Each comparison, ordered by outcome and cohort size. Filled circles use the mean of the ML families and open circles the best family; the columns show features in which the comparisons differ. (**B**) By cohort size (recipients as listed in Table 4). Each point is one comparison and uses the mean of the ML families. Two of the points coincide. No trend line is fitted. (**C**) Each family of ML models against the regression model of the same comparison. A comparison contributes one point to each family that it evaluated. The families were evaluated in different comparisons, so the rows cannot be compared with one another, and no summary value is shown. Orange, delayed graft function (DGF); blue, graft survival. The shaded band marks differences within 0.02 and is a visual guide, not an equivalence margin. No pooled difference is shown. AUC, area under the receiver operating characteristic curve.

Twenty-seven reports contributed 28 comparisons in which ML and regression were evaluated in the same cohort under the same evaluation design: 8 for DGF and 20 for graft survival (Table 4, Figure 6A). One further report evaluated the two classes under different designs, and one compared its models with a published index without stating the sample in which the index was evaluated; both are listed in Supplementary Table S5 without being summarized. The comparisons were heterogeneous. Cohorts ranged from 108 to 228,855 recipients. The metric was an AUC in 16 comparisons and a C-statistic in 12. The evaluation was external in 3, temporal or geographic in 3, internal in 20, and not reported in 2. Both model classes used the same predictors in 22, and in 3, the regression comparator was a published model applied unchanged. The authors of the report tested the difference between ML and regression statistically in seven comparisons. A further 12 reports fitted models of both classes but did not report an AUC or C-statistic for both.

The median difference between ML and regression was 0.01 (IQR −0.02 to 0.03; range −0.15 to 0.20). ML exceeded regression by more than 0.02 in 11 comparisons, regression exceeded ML by more than 0.02 in 7, and the difference was within 0.02 in 10. Taking the best ML family in each report instead of the mean of the families raised the difference by 0.03 on average. The median difference was 0.00 for DGF and 0.01 for graft survival. By cohort size, it was −0.03 in the 9 comparisons with fewer than 1000 recipients, 0.01 in the 9 with 1000 to 9999, and 0.01 in the 10 with at least 10,000 (Figure 6B). Figure 6C shows the differences for each ML family separately: tree ensembles were evaluated in 23 comparisons, neural networks in 14, support-vector machines in 10, and each of the other families in 5 or fewer. The families were evaluated in different comparisons and are not compared with one another. These differences are descriptive. They do not estimate a common effect, and they cannot show that either approach is superior or inferior.

### 3.5. Risk of Bias, Calibration and Validation

**Figure 7:**
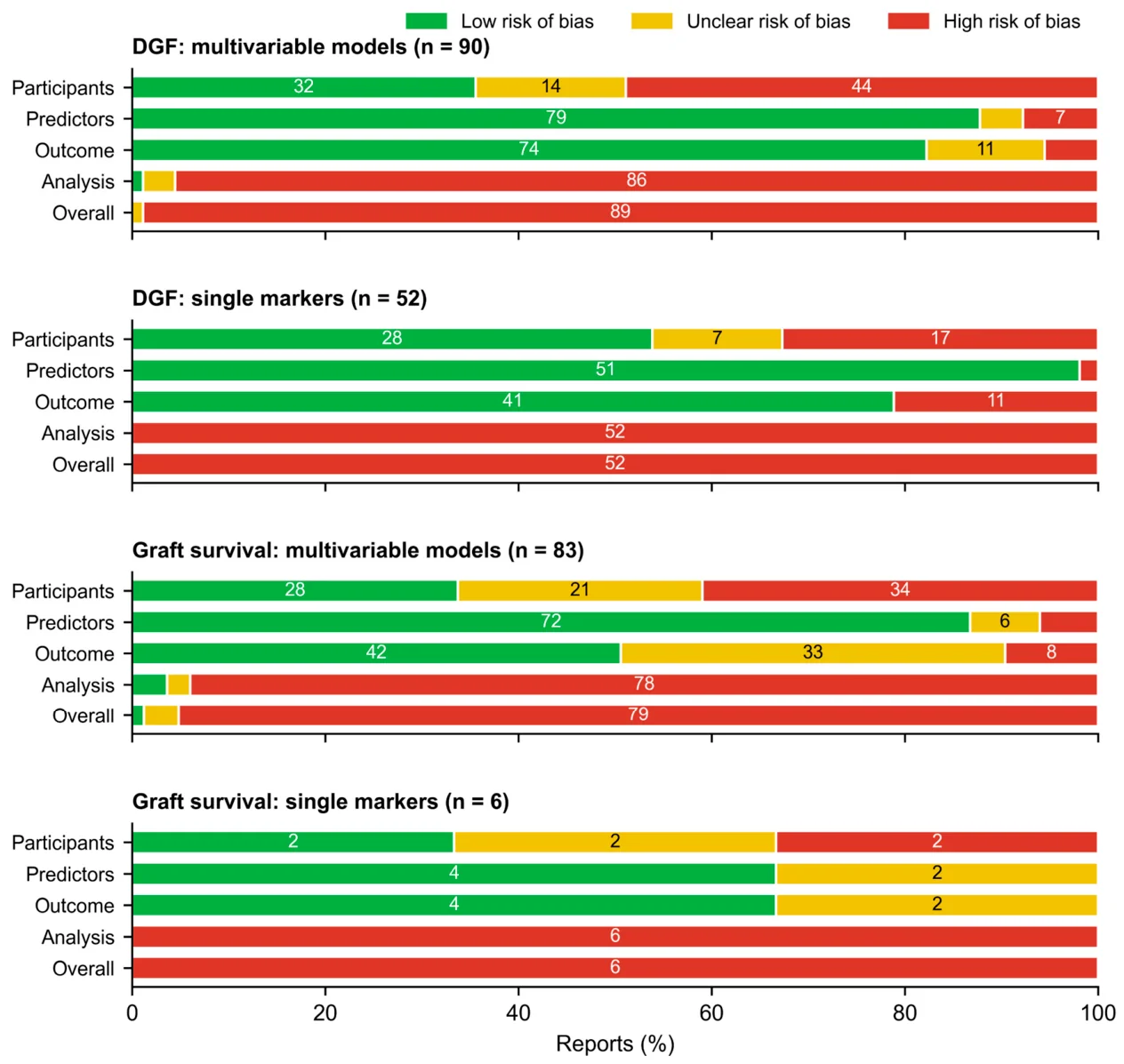
PROBAST risk-of-bias judgments by domain, for reports of multivariable models and of single markers. Numbers inside the bars are the number of reports. DGF, delayed graft function.

All 212 non-pediatric reports were appraised with PROBAST (Table 5, Figure 7). Overall risk of bias was high in 208 (98%), unclear in 3, and low in 1. The analysis domain accounted for most of these judgments. Among the 156 reports of multivariable models, the number of events was too small for the number of candidate predictors in 104; predictors were selected by univariable screening in 65; calibration was not assessed in 85 (it could not be judged in the one report assessed from the abstract), and optimism was not addressed in 89.

Calibration was assessed in 70 of the 156 model reports (45%), and 11 reported a calibration slope or intercept (Table 6). Handling of missing data was not described in 72, and 18 used multiple imputation. Internal validation was absent in 44 reports, was a random split alone in 49, and was cross-validation or bootstrapping in 46. External validation was reported in 42, an assessment of clinical usefulness in 29, and performance by subgroup in 23. Table 5 and Table 6 give these counts by outcome; a report that addressed both outcomes appears under each.

Five pediatric reports were not combined with the other evidence: Kaboré and colleagues reported an AUC of 0.87 for graft failure (internal validation) [73]; Liu and colleagues reported an AUC of 0.91 for DGF (internal validation) [74]; Suh and colleagues reported an AUC of 0.81 for graft failure (internal validation) [75]; Patry and colleagues reported an AUC of 0.88 for DGF (internal validation) [76]; Mishra and colleagues reported no AUC for DGF [77].

## 4. Discussion

The meta-analysis in this review concerns published regression scores only; ML and regression were compared descriptively. The review reached three findings. The Irish 2010 model, the most often validated DGF model, discriminated modestly outside the cohort in which it was built, and other named scores did no better. Neither ML nor regression was consistently better when both were evaluated in the same patients: the differences ran in both directions and centered near zero, and the comparisons were too different to rank the two approaches. Almost every report was at high risk of bias, and about half of the model reports assessed calibration.

The comparison of ML with regression needs careful reading. Our results agree with a multicenter study that found no advantage of ML over Cox models for kidney allograft failure [4] and with a review of clinical prediction models in general [8]. They do not show that ML is inferior. The comparisons differed in outcome, metric, predictors, tuning and validation design, and few reported the uncertainty of the difference. For that reason, we did not pool them, and we present each comparison with the features that distinguish it. A fair test would give both approaches the same predictors, outcome, and horizon, tune both with equal care, and evaluate both in an independent cohort for discrimination, calibration, and clinical usefulness. Until such comparisons exist, neither the superiority nor the inferiority of ML is established.

Reported discrimination was highest where a model was evaluated in its own development data. For DGF, models assessed only in their development data reported the highest values, and models tested in independent cohorts reported the lowest. This contrast is not paired, because different models were evaluated at each level. Where a report gave both values for the same model, the development value was higher by a median of 0.01 for DGF (0.02 in the 12 reports with an external cohort) and by 0.05 for graft survival, where the validation was internal in 20 of 23. For DGF, optimism therefore accounts for only part of the contrast; the rest reflects which models were ever taken to an independent cohort. Either way, performance in the development data is not evidence of performance elsewhere. The Irish 2010 model is the most often validated DGF model, and its discrimination was modest in every cohort that tested it. A program that adopts it should expect the same and should check its calibration locally, because the model overestimated risk in one of the five cohorts that assessed calibration and in pump-perfused kidneys in another report. Competing clinical scores were tested in fewer cohorts and performed no better.

For graft survival, single-center models reported higher discrimination than registry models, and models that used posttransplant information reported higher discrimination than models restricted to information available at the time of the offer. These contrasts reflect the clinical question as much as the method. A model that incorporates early graft function, biopsy findings, or biomarkers supports monitoring after transplantation; it cannot guide organ acceptance.

Risk of bias limits confidence in the reported performance of regression and ML models alike. Small numbers of events, data-driven selection of predictors, missing calibration, and unaddressed optimism were common to both. The central problem is not the class of model but the lack of rigorous development, complete reporting, and independent validation.

### 4.1. Clinical Implications

**Figure 8:**
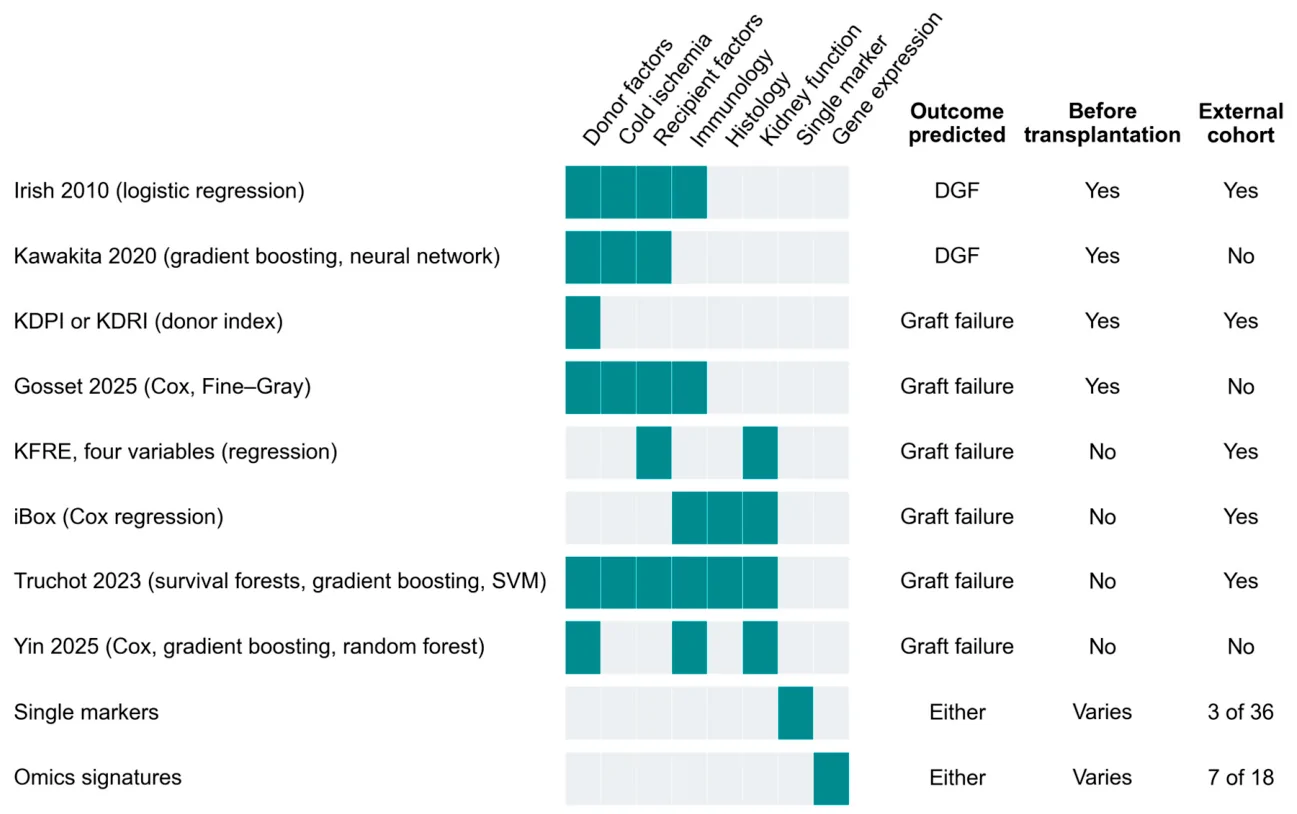
Types of predictors used by eight prediction models and by the two classes of marker study. The models were chosen by the authors as examples of those reviewed; the list is not exhaustive. Predictors are those described in the reports cited [4,5,18,72,78,79]; those of the KDPI and KDRI are as summarized in [35]. A shaded cell means that the model uses at least one predictor of that type. The last three columns give the outcome predicted, whether the prediction is available before transplantation, and whether an external cohort evaluated the model in the reports reviewed. Models that use cold ischemia time need an expected value when they are applied before transplantation. For single markers and omics signatures, the last column counts the reports whose estimate in Table 2 was of that class (a report on both outcomes counted once) and, of these, the reports with an estimate from an external cohort. DGF, delayed graft function; KDPI, Kidney Donor Profile Index; KDRI, Kidney Donor Risk Index; KFRE, Kidney Failure Risk Equation; SVM, support-vector machine.

A surgeon weighing a kidney offer, or counseling a recipient, needs a prediction that is available before transplantation and that has been shown to hold in patients like the one in front of them. Few published models meet that standard. Before a model is used, its user should know when the prediction is made, which variables it needs, and how it performed, in discrimination and calibration, in an independent cohort (Table 7). Figure 8 shows, for eight models chosen as examples and for the two classes of marker study, the types of predictors used, the outcome predicted, whether the prediction is available before transplantation, and whether an external cohort has tested it. Missing inputs, changes in practice between eras and centers, and poor calibration in subgroups can each degrade a model after deployment.

Prediction models used in allocation can also introduce bias. A model trained on past acceptance decisions may reproduce them, and a score that is poorly calibrated in a subgroup may reduce access for patients who would have benefited from transplantation. Few of the included studies assessed performance by subgroup, and none evaluated the effect of a model on clinical decisions. Local recalibration and prospective evaluation should therefore precede any use of a model in organ acceptance.

### 4.2. Limitations

The protocol was not registered. The graft-survival search included discrimination terms, so we may have missed graft-survival prediction studies that reported no such statistic. Keyword and citation searching did not retrieve the development report of every named model that the included studies evaluated, for example, the iBox [80], and such reports were not added outside the searches. Pooling was possible only for named models with few cohorts each; heterogeneity was moderate for the Irish 2010 model and substantial for the Chapal score and Jeldres nomogram; the Irish 2003 nomogram was pooled from three cohorts, and the pooled values of these scores are imprecise. Values printed only inside a figure may have been missed in some reports. All other estimates are descriptive, and the choice of one estimate per report, although prespecified, affects the medians. Outcome definitions, prediction times, and validation designs varied, and several omics studies analyzed the same public datasets, so their estimates are not independent. PROBAST judgments depend on what a report states, and a poorly reported study may be rated at higher risk than its conduct deserves. One report was assessed from the abstract because its full text could not be obtained, so its extracted data and risk-of-bias judgments rest on less information than the others. Finally, we used published summary measures and could not recalculate discrimination under uniform definitions.

## 5. Conclusions

Current evidence does not show that ML models predict DGF or graft survival better than regression, and it cannot show that they are worse. The best-validated DGF model discriminates modestly in new populations, and most published models have not been independently validated or calibrated. Before a prediction model informs the decision to accept a kidney or the counseling of a recipient, it should be validated in an independent cohort, report calibration, and use only information available when that decision is made.

## Figures and Tables

**Table 1:** Evidence included, by outcome.

Evidence	Delayed Graft Function	Graft Survival
Reports included	145	91
Pediatric reports (summarized separately)	3	2
Non-pediatric reports analyzed	142	89
Model development only	48	44
Model development with validation	31	28
External validation of an existing model only	11	9
Discrimination of a single marker	52	6
Other prediction report	0	2
Single-center cohort	98	23
Registry or multicenter cohort	33	46
Public dataset	11	19
Setting not reported	0	1
Reports with an AUC or C-statistic	135	78
Same-cohort comparisons of ML and regression	8	20
Reports at high overall risk of bias	141 of 142 (99%)	85 of 89 (96%)

Counts below the third row refer to non-pediatric reports. Reports that addressed both outcomes appear in both columns. ML, machine learning; AUC, area under the receiver operating characteristic curve.

**Table 2:** Reported discrimination by outcome, model class, evaluation design and setting.

Outcome and Subgroup	Reports	Median AUC or C-Statistic	IQR	Range
**Delayed graft function (recipient-level occurrence)**				
All reports	120	0.78	0.71–0.87	0.50–1.00
Regression or clinical score	59	0.79	0.73–0.85	0.50–0.96
Machine learning	17	0.81	0.72–0.87	0.62–1.00
Single biomarker	34	0.78	0.65–0.88	0.51–0.98
Omics signature	10	0.75	0.71–0.77	0.69–0.97
Development data only (apparent)	58	0.82	0.74–0.89	0.51–0.98
Internal validation	28	0.78	0.74–0.84	0.62–1.00
Temporal or geographic validation	11	0.78	0.73–0.86	0.70–0.91
External cohort	22	0.72	0.70–0.76	0.50–0.88
Evaluation design not reported	1	0.92	0.92–0.92	0.92–0.92
Single center	79	0.82	0.75–0.88	0.54–1.00
Registry or multicenter	30	0.73	0.65–0.78	0.50–0.90
Public dataset	11	0.74	0.71–0.81	0.69–0.97
**Graft survival**				
All reports	78	0.74	0.67–0.84	0.54–0.97
Regression or clinical score	32	0.72	0.65–0.81	0.54–0.91
Machine learning	29	0.76	0.70–0.84	0.59–0.97
Single biomarker	4	0.71	0.68–0.75	0.66–0.84
Omics signature	13	0.78	0.72–0.88	0.67–0.92
Development data only (apparent)	14	0.76	0.68–0.85	0.61–0.92
Internal validation	47	0.75	0.69–0.85	0.54–0.97
Temporal or geographic validation	3	0.62	0.60–0.63	0.59–0.63
External cohort	13	0.73	0.63–0.79	0.54–0.85
Evaluation design not reported	1	0.71	0.71–0.71	0.71–0.71
Single center	18	0.85	0.73–0.88	0.59–0.95
Registry or multicenter	42	0.69	0.65–0.79	0.54–0.97
Public dataset	18	0.77	0.72–0.86	0.67–0.92
Prediction before or at transplantation	32	0.69	0.65–0.74	0.59–0.97
Prediction from a post-transplant landmark	29	0.81	0.73–0.86	0.54–0.91
Dynamic prediction	8	0.79	0.71–0.81	0.54–0.95
Prediction time not reported	9	0.78	0.72–0.80	0.67–0.92
Registry or multicenter, 1-year horizon	12	0.70	0.66–0.75	0.59–0.87
Registry or multicenter, 3-year horizon	8	0.68	0.62–0.73	0.60–0.74
Registry or multicenter, 5-year horizon	14	0.68	0.63–0.74	0.54–0.81
Registry or multicenter, 10-year horizon	10	0.73	0.68–0.75	0.60–0.79

One estimate per report: the final model in its most independent evaluation. Values are descriptive and were not pooled. IQR, interquartile range.

**Table 4:** Comparisons of machine learning and regression in the same cohort.

Report	Outcome	Recipients	Metric	Evaluation	Same Predictors	Regression	ML (Mean of Families)	Difference
Feng 2026 [37]	DGF	140	AUC	Temporal or geographic	Yes	0.839	0.810	−0.029
Brier 2003 [69]	DGF	304	AUC	Internal	Yes	0.636	0.668	0.032
Costa 2020 [42]	DGF	443	AUC	Internal	Yes	0.695	0.820	0.125
Decruyenaere 2015b [38]	DGF	497	AUC	Internal	Yes	0.817	0.729	−0.088
Salgado 2026 [70]	DGF	523	AUC	Internal	Yes	0.670	0.522	−0.148
Jen 2021 [71]	DGF	2089	AUC	Internal	No	0.748	0.760	0.011
Kawakita 2020 [72]	DGF	55,044	AUC	Internal	Yes	0.728	0.735	0.007
Bae 2020 [39]	DGF	133,431	AUC	Temporal or geographic	Yes	0.721	0.720	−0.001
Milecki 2025 [53]	Graft survival, 5 y	108	C-statistic	External	No	0.665	0.613	−0.052
Mulugeta 2025 [54]	Graft survival	278	C-statistic	Internal	Yes	0.881	0.910	0.028
Shen 2026 [55]	Graft survival, 1 y	356	AUC	Not reported	Yes	0.723	0.616	−0.107
Hamidi 2016 [56]	Graft survival	378	C-statistic	Internal	Unclear	0.766	0.965	0.199
Achilonu 2024 [57]	Graft survival, 10 y	1738	AUC	Internal	Yes	0.670	0.815	0.145
Garcia-Lopez 2026 [58]	Graft survival, 1 y	2030	C-statistic	Internal	Yes	0.720	0.870	0.150
Yin 2025 [5]	Graft survival, 10 y	2575	AUC	Internal	Yes	0.868	0.849	−0.020
Yoo 2017 [59]	Graft survival	3117	C-statistic	Internal	Yes	0.650	0.745	0.095
Tang 2011 [60]	Graft survival, 3 y	3313	AUC	Internal	Yes	0.740	0.705	−0.035
Fan 2025 [61]	Graft survival, 1 y	4728	AUC	Internal	Yes	0.728	0.753	0.026
Senanayake 2021 [62]	Graft survival	7365	C-statistic	Internal	Yes	0.670	0.613	−0.057
Truchot 2023 [4]	Graft survival, 7 y	8422	C-statistic	External	Published model	0.826	0.832	0.006
Ali 2024c [63]	Graft survival	29,713	C-statistic	Not reported	Published model	0.570	0.640	0.070
Salaün 2024 [64]	Graft survival	36,653	C-statistic	Internal	Yes	0.618	0.628	0.010
Naqvi 2021 [3]	Graft survival, 5 y	52,827	AUC	Internal	Yes	0.620	0.653	0.033
Potluri 2026 [65]	Graft survival, 3 y	75,867	Time-dependent AUC	Internal	Yes	0.659	0.665	0.006
Bae 2020 [39]	Graft survival	133,431	C-statistic	Temporal or geographic	Yes	0.632	0.631	−0.001
Ali 2024a [66]	Graft survival, 1 y	156,749	Time-dependent AUC	External	Published model	0.590	0.620	0.030
Paquette 2022 [67]	Graft survival	180,141	C-statistic	Internal	Yes	0.646	0.650	0.004
Apanisile 2026 [68]	Graft survival	228,855	C-statistic	Internal	Yes	0.687	0.686	−0.001

Each row is one comparison. Regression and ML values are AUCs or C-statistics from the evaluation named in the fifth column. The difference is ML minus regression, calculated before rounding. Published model: the regression comparator was a published model applied unchanged, so its predictors differ from those of the ML model. For Ali 2024a, the comparison was made in the United Kingdom validation cohort of the report; the number of recipients is that of its development cohort. DGF, delayed graft function; ML, machine learning.

**Table 5:** PROBAST risk-of-bias judgments.

PROBAST Domain	Low Risk	Unclear Risk	High Risk
**DGF, multivariable models (*n* = 90)**			
Participants	32 (36%)	14 (16%)	44 (49%)
Predictors	79 (88%)	4 (4%)	7 (8%)
Outcome	74 (82%)	11 (12%)	5 (6%)
Analysis	1 (1%)	3 (3%)	86 (96%)
Overall	0 (0%)	1 (1%)	89 (99%)
**DGF, single markers (*n* = 52)**			
Participants	28 (54%)	7 (13%)	17 (33%)
Predictors	51 (98%)	0 (0%)	1 (2%)
Outcome	41 (79%)	0 (0%)	11 (21%)
Analysis	0 (0%)	0 (0%)	52 (100%)
Overall	0 (0%)	0 (0%)	52 (100%)
**Graft survival, multivariable models (*n* = 83)**			
Participants	28 (34%)	21 (25%)	34 (41%)
Predictors	72 (87%)	6 (7%)	5 (6%)
Outcome	42 (51%)	33 (40%)	8 (10%)
Analysis	3 (4%)	2 (2%)	78 (94%)
Overall	1 (1%)	3 (4%)	79 (95%)
**Graft survival, single markers (*n* = 6)**			
Participants	2 (33%)	2 (33%)	2 (33%)
Predictors	4 (67%)	2 (33%)	0 (0%)
Outcome	4 (67%)	2 (33%)	0 (0%)
Analysis	0 (0%)	0 (0%)	6 (100%)
Overall	0 (0%)	0 (0%)	6 (100%)

Values are numbers of reports (%). For single-marker reports, the questions on calibration, univariable selection and model weights were not applicable.

**Table 6:** Reporting and validation characteristics of multivariable model reports.

Characteristic	DGF Models (n = 90)	Graft-Survival Models (n = 83)
Too few events for the candidate predictors	66 of 90 (73%)	48 of 83 (58%)
Predictors selected by univariable screening	46 of 90 (51%)	26 of 83 (31%)
Handling of missing data not described	46 of 90 (51%)	35 of 83 (42%)
Multiple imputation	6 of 90 (7%)	15 of 83 (18%)
Any calibration assessment	38 of 90 (42%)	42 of 83 (51%)
Calibration slope or intercept	7 of 90 (8%)	7 of 83 (8%)
No internal validation	34 of 90 (38%)	14 of 83 (17%)
Random split only	22 of 90 (24%)	35 of 83 (42%)
Cross-validation or bootstrap	24 of 90 (27%)	24 of 83 (29%)
External validation reported	26 of 90 (29%)	27 of 83 (33%)
Clinical usefulness assessed	14 of 90 (16%)	15 of 83 (18%)
Performance by subgroup assessed	11 of 90 (12%)	15 of 83 (18%)

Values are numbers of reports (%).

**Table 7:** Questions to ask before using a kidney-transplant prediction model.

Question	Why It Matters
Was the model externally validated?	Performance should be tested in patients who were not used to develop the model, preferably at other centers.
Was calibration reported?	A model can rank patients correctly and still overestimate or underestimate their risk.
Was the development cohort large enough?	Few events for many candidate predictors lead to overfitting and unstable estimates.
Are the predictors available when the decision is made?	A model for organ acceptance cannot use information collected after transplantation.
Does the model improve on a validated alternative?	Added complexity is useful only if it improves prediction, calibration, or decisions.

## Data Availability

The estimate taken from each report, the risk-of-bias judgments, and the reports excluded at full text are provided in the Supplementary Materials. The full extraction dataset and the analysis code are available from the corresponding author on request.

## Supplementary Materials

The following supporting information can be downloaded at https://www.mdpi.com/article/10.3390/jcm15218535/s1, Table S1: Search strategies, limits and yields; Table S2: Characteristics and discrimination of delayed graft function reports; Table S3: External validations of named models and sensitivity analyses; Table S4: Characteristics and discrimination of graft-survival reports; Table S5: Comparisons of machine learning and regression in the same cohort; Table S6: PROBAST signaling questions and judgments for each report; Table S7: Reports excluded at full-text assessment, with reasons. Supplementary File S1: PRISMA 2020 checklist.
