# Supplementary material for: Machine Learning for Predicting Delayed Graft Function and Graft Survival After Kidney Transplantation: A Systematic Review and Meta-Analysis
Source: J Clin Med. 2026 Nov 2;15(21):8535. doi: 10.3390/jcm15218535 (PMC13764368; doi:10.3390/jcm15218535)
Supplement: Supplementary file 1 [file jcm-15-08535-s001.zip › Supplementary File S1.pdf]

Supplementary File S1

PRISMA 2020 checklist

Machine Learning for Predicting Delayed Graft Function and Graft Survival After Kidney Transplantation: A Systematic Review and Meta-Analysis

Manuscript jcm-4536474 • Second revision • 10 October 2026

Locations refer to the accompanying 26-page clean manuscript and Supplementary Tables S1–S7. The PRISMA 2020 for Abstracts checklist follows the main checklist.

Item	Section or topic	PRISMA 2020 checklist item	Location in manuscript
1	Title	Identify the report as a systematic review.	Title, p. 1: identifies a systematic review and meta-analysis.
2	Abstract	See the PRISMA 2020 for Abstracts checklist.	Abstract, p. 1; see the accompanying PRISMA 2020 for Abstracts checklist.
3	Rationale	Describe the rationale for the review in the context of existing knowledge.	Section 1, pp. 1–2: existing prediction models, validation limitations, previous reviews and the reason for this review.
4	Objectives	Provide an explicit statement of the objective(s) or question(s) the review addresses.	Section 1, p. 2, final paragraph: discrimination, calibration, validation, risk of bias, named-model meta-analysis and same-cohort ML–regression comparisons.
5	Eligibility criteria	Specify the inclusion and exclusion criteria for the review and how studies were grouped for the syntheses.	Sections 2.1–2.2, p. 2; Sections 2.5–2.7, pp. 3–4: eligibility, outcome definitions, pediatric separation and grouping/selection for each synthesis.
6	Information sources	Specify all databases, registers, websites, organisations, reference lists and other sources searched or consulted to identify studies. Specify the date when each source was last searched or consulted.	Section 2.3, p. 3; Table S1 and accompanying search documentation. Main database cutoff: 28 June 2026. Separate last-consulted dates for Consensus and Europe PMC are not reported.
7	Search strategy	Present the full search strategies for all databases, registers and websites, including any filters and limits used.	Section 2.3, p. 3; Table S1 and accompanying search documentation: saved search strings, limits and yields. The additional PubMed DGF search, PubMed graft-survival search and Consensus searches are documented as concept summaries.
8	Selection process	Specify the methods used to decide whether a study met the inclusion criteria of the review, including how many reviewers screened each record and each report retrieved, whether they worked independently, and if applicable, details of automation tools used in the process.	Section 2.4, p. 3: independent title/abstract screening by two reviewers, consensus resolution and full-text assessment by both reviewers.
9	Data collection process	Specify the methods used to collect data from reports, including how many reviewers collected data from each report, whether they worked independently, any processes for obtaining or confirming data from study investigators, and if applicable, details of automation tools used in the process.	Section 2.5, p. 3: duplicate extraction, prespecified form, source checking, consensus resolution and independent human review of machine-assisted extraction.

Item	Section or topic	PRISMA 2020 checklist item	Location in manuscript
10a	Data items	List and define all outcomes for which data were sought. Specify whether all results that were compatible with each outcome domain in each study were sought (e.g. for all measures, time points, analyses), and if not, the methods used to decide which results to collect.	Sections 2.2, 2.5 and 2.7, pp. 2–4: DGF/graft-failure definitions, metrics, horizons, prediction times, all reported discrimination estimates, and the rule for selecting one estimate per report/outcome.
10b	Data items	List and define all other variables for which data were sought (e.g. participant and intervention characteristics, funding sources). Describe any assumptions made about any missing or unclear information.	Section 2.5, p. 3; Tables S2–S5: study, population, model and validation variables; missing information indicated as not reported.
11	Study risk of bias assessment	Specify the methods used to assess risk of bias in the included studies, including details of the tool(s) used, how many reviewers assessed each study and whether they worked independently, and if applicable, details of automation tools used in the process.	Section 2.6, pp. 3–4; Table S6: PROBAST domains, signaling questions, judgment rules, single-marker adaptations and independent review of all assessments.
12	Effect measures	Specify for each outcome the effect measure(s) (e.g. risk ratio, mean difference) used in the synthesis or presentation of results.	Sections 2.5 and 2.7, pp. 3–4: AUC/C-statistic; logit-transformed AUC for pooling; ML minus regression discrimination differences.
13a	Synthesis methods	Describe the processes used to decide which studies were eligible for each synthesis (e.g. tabulating the study intervention characteristics and comparing against the planned groups for each synthesis (item #5)).	Sections 2.1–2.2, p. 2; Sections 2.5–2.7, pp. 3–4: report-level estimate selection, named-model external-validation eligibility, pediatric separation and same-cohort comparison rules.
13b	Synthesis methods	Describe any methods required to prepare the data for presentation or synthesis, such as handling of missing summary statistics, or data conversions.	Sections 2.5–2.7, pp. 3–4; Table S3: medians where several estimates qualify, horizon selection, logit conversion, Hanley–McNeil standard errors for sensitivity analyses and within-family ML averaging.
13c	Synthesis methods	Describe any methods used to tabulate or visually display results of individual studies and syntheses.	Section 2.7, p. 4; Tables 2–4 and Figures 2–6, pp. 8–17: tabulated estimates, descriptive distributions, forest/funnel plots and comparison displays; captions explain symbols and summaries.
13d	Synthesis methods	Describe any methods used to synthesize results and provide a rationale for the choice(s). If meta-analysis was performed, describe the model(s), method(s) to identify the presence and extent of statistical heterogeneity, and software package(s) used.	Section 2.7, p. 4: named-model random-effects pooling, DerSimonian–Laird variance, Hartung–Knapp–Sidik–Jonkman adjustment, prediction intervals, I^2/τ^2, descriptive medians, rationale against unrelated-model pooling, Python/NumPy/SciPy.
13e	Synthesis methods	Describe any methods used to explore possible causes of heterogeneity among study results (e.g. subgroup analysis, meta-regression).	Section 2.7, p. 4: descriptive grouping by outcome, model class, evaluation design and setting.
13f	Synthesis methods	Describe any sensitivity analyses conducted to assess robustness of the synthesized results.	Section 2.7, p. 4; Table S3: inclusion of cohorts without reported intervals and leave-one-cohort-out analysis where at least four cohorts were pooled.
14	Reporting bias assessment	Describe any methods used to assess risk of bias due to missing results in a synthesis (arising from reporting biases).	Section 2.7, p. 4: descriptive funnel-plot assessment for Irish 2010; formal asymmetry testing was not performed, with rationale.
15	Certainty assessment	Describe any methods used to assess certainty (or confidence) in the body of evidence for an outcome.	Section 2.7, p. 4: certainty grading was not performed, with rationale.

Item	Section or topic	PRISMA 2020 checklist item	Location in manuscript
16a	Study selection	Describe the results of the search and selection process, from the number of records identified in the search to the number of studies included in the review, ideally using a flow diagram.	Section 3.1, p. 5; Figure 1, p. 6: search and selection flow, including 217 included reports.
16b	Study selection	Cite studies that might appear to meet the inclusion criteria, but which were excluded, and explain why they were excluded.	Section 2.4, p. 3; Table S7: excluded full-text reports identified by author/year and PMID, with reasons.
17	Study characteristics	Cite each included study and present its characteristics.	Table 1, p. 7; Tables S2 and S4: included reports and characteristics; Section 3.5, p. 18, separately describes pediatric reports.
18	Risk of bias in studies	Present assessments of risk of bias for each included study.	Section 3.5, pp. 18–20; Table 5, pp. 18–19; Figure 7, p. 19; Table S6: report-level signaling answers, domain ratings and overall risk-of-bias judgments.
19	Results of individual studies	For all outcomes, present, for each study: (a) summary statistics for each group (where appropriate) and (b) an effect estimate and its precision (e.g. confidence/credible interval), ideally using structured tables or plots.	Tables S2–S5; Table 3, pp. 11–12; Figures 3–4, pp. 10 and 13; Section 3.5, p. 18: individual estimates, reported precision, sample sizes and pediatric results. Table S4 includes source-level graft-survival intervals; intervals are not assigned to medians across models or cohorts.
20a	Results of syntheses	For each synthesis, briefly summarise the characteristics and risk of bias among contributing studies.	Sections 3.2–3.5, pp. 7–20; Tables 1–6 and S2–S6: contributing-study characteristics and report-level risk-of-bias assessments.
20b	Results of syntheses	Present results of all statistical syntheses conducted. If meta-analysis was done, present for each the summary estimate and its precision (e.g. confidence/credible interval) and measures of statistical heterogeneity. If comparing groups, describe the direction of the effect.	Sections 3.2–3.4, pp. 7–17; Tables 2–4; Figures 2–6; Table S3: pooled AUCs, confidence/prediction intervals and heterogeneity, descriptive summaries and signed ML–regression differences.
20c	Results of syntheses	Present results of all investigations of possible causes of heterogeneity among study results.	Sections 3.2–3.4, pp. 7–17; Table 2; Figures 2, 5 and 6: descriptive contrasts by evaluation design, model class, setting, prediction horizon and cohort characteristics.
20d	Results of syntheses	Present results of all sensitivity analyses conducted to assess the robustness of the synthesized results.	Section 3.2.1, pp. 10–11; Table S3: leave-one-out results and inclusion of cohorts without reported intervals.
21	Reporting biases	Present assessments of risk of bias due to missing results (arising from reporting biases) for each synthesis assessed.	Section 3.2.1, p. 10; Figure 4B, p. 13: descriptive Irish 2010 funnel-plot findings; rationale for not testing asymmetry in Section 2.7, p. 4.
22	Certainty of evidence	Present assessments of certainty (or confidence) in the body of evidence for each outcome assessed.	Section 2.7, p. 4: no formal certainty grading; limitations of the evidence discussed in Section 4, pp. 20–22.
23a	Discussion	Provide a general interpretation of the results in the context of other evidence.	Section 4, p. 20: interpretation against previous transplant and general clinical-prediction evidence.
23b	Discussion	Discuss any limitations of the evidence included in the review.	Section 4, p. 20; Section 4.2, p. 22: bias, calibration/validation deficits, heterogeneous comparisons, sparse model-specific pooling and overlapping datasets.

Item	Section or topic	PRISMA 2020 checklist item	Location in manuscript
23c	Discussion	Discuss any limitations of the review processes used.	Section 4.2, p. 22: unregistered protocol, search limitations, estimate selection, figure-only values, abstract-only assessment and use of published summary data.
23d	Discussion	Discuss implications of the results for practice, policy, and future research.	Section 4.1, pp. 20–21; Table 7 and Section 5, p. 22: independent validation, calibration, clinical decision timing, fairness and prospective evaluation.
24a	Registration and protocol	Provide registration information for the review, including register name and registration number, or state that the review was not registered.	Sections 2.1, p. 2, and 4.2, p. 22: the review was not prospectively registered.
24b	Registration and protocol	Indicate where the review protocol can be accessed, or state that a protocol was not prepared.	Section 2.1, p. 2 describes the prespecified protocol; an access location is not reported.
24c	Registration and protocol	Describe and explain any amendments to information provided at registration or in the protocol.	Section 2.1, p. 2: protocol amendments concerning pediatric reports, named-model pooling and inclusion of reports without discrimination statistics.
25	Support	Describe sources of financial or non-financial support for the review, and the role of the funders or sponsors in the review.	Funding, p. 23: no specific grant from public, commercial or not-for-profit agencies.
26	Competing interests	Declare any competing interests of review authors.	Conflicts of Interest, p. 23: R.G. is guest editor of the special issue; other authors declare no conflicts.
27	Availability of data, code and other materials	Report which of the following are publicly available and where they can be found: template data collection forms; data extracted from included studies; data used for all analyses; analytic code; any other materials used in the review.	Data Availability Statement, p. 23; Tables S2–S7: supplementary extracted data, risk-of-bias judgments and exclusions; full extraction dataset and analysis code available from the corresponding author on request.

Source: Page MJ, McKenzie JE, Bossuyt PM, et al. The PRISMA 2020 statement: an updated guideline for reporting systematic reviews. BMJ. 2021;372:n71. https://doi.org/10.1136/bmj.n71. Checklist wording reproduced from https://www.prisma-statement.org/prisma-2020-checklist under CC BY 4.0 (https://creativecommons.org/licenses/by/4.0/); layout adapted and manuscript locations added.

PRISMA 2020 for Abstracts

All locations below refer to the title and abstract on manuscript p. 1. This mapping supports main-checklist item 2.

Item	Section or topic	Abstract checklist requirement	Location and reporting detail
1	Title	Identify the report as a systematic review.	Reported in the title.
2	Objectives	Provide an explicit statement of the main objective(s) or question(s) the review addresses.	Reported in Background/Objectives: ML compared with regression and the adequacy of validation.
3	Eligibility criteria	Specify the inclusion and exclusion criteria for the review.	Clinical/model scope stated; detailed eligibility criteria not provided.
4	Information sources	Specify the information sources used to identify studies and the date when each was last searched.	PubMed/MEDLINE, Embase, Web of Science, the 28 June 2026 cutoff and named-model citation searching stated. Consensus/Europe PMC and source-specific dates are not specified in the abstract.
5	Risk of bias	Specify the methods used to assess risk of bias in the included studies.	Reported: PROBAST.
6	Synthesis of results	Specify the methods used to present and synthesise results.	Pooling restricted to external validations of the same model; other summary methods not described in the abstract.
7	Included studies	Give the total number of included studies and participants and summarise relevant characteristics of studies.	217 reports and outcome counts; participant total not reported.
8	Synthesis of results	Present results for main outcomes, preferably indicating numbers of included studies and participants; report meta-analysis estimates and intervals and direction of group differences.	Irish 2010 pooled AUC and CI with seven cohorts, and 28 paired comparisons. Participant counts and a separate graft-survival discrimination summary are not provided in the abstract.
9	Limitations of evidence	Provide a brief summary of the limitations of the evidence included in the review.	Reported: high risk of bias and heterogeneous comparisons.
10	Interpretation	Provide a general interpretation of the results and important implications.	Reported in Conclusions.
11	Funding	Specify the primary source of funding for the review.	Not reported in the abstract; see main-text Funding statement.
12	Registration	Provide the register name and registration number.	No registration identifier; unregistered status stated in Section 2.1.

Abstract requirements adapted from Table 2 of Page et al., PRISMA 2020, https://pmc.ncbi.nlm.nih.gov/articles/PMC8007028/. CC BY 4.0.
