# Supplementary material for: Machine Learning for Predicting Delayed Graft Function and Graft Survival After Kidney Transplantation: A Systematic Review and Meta-Analysis
Source: J Clin Med. 2026 Nov 2;15(21):8535. doi: 10.3390/jcm15218535 (PMC13764368; doi:10.3390/jcm15218535)
Supplement: Supplementary file 1 [file jcm-15-08535-s001.zip › Supplementary Tables.pdf]

Supplementary Materials

Machine Learning for Predicting Delayed Graft Function and Graft Survival After Kidney Transplantation: A Systematic Review and Meta-Analysis

Table S1. Search strategies, limits and yields

Source	Outcome	Search string or strategy	Limits	Records	Retained for screening
PubMed/MEDLINE	DGF	Saved broad query plus concept summary: (delayed graft function) AND (machine learning OR artificial intelligence OR deep learning OR prediction model) AND kidney transplant (311 records), combined with a discrimination-focused set of DGF prediction records (AUC, C-statistic or ROC terms)	No limits	380	380
PubMed/MEDLINE	Graft survival	Concept summary: Kidney or renal transplantation AND (graft survival OR graft failure OR graft loss) AND prediction-model or machine-learning terms AND discrimination terms (AUC, C-statistic, concordance)	No limits	107	100 (6 also in DGF search; 1 systematic review)
Embase (Ovid, 1947–25 June 2026)	DGF	delayed graft function and (machine learning or artificial intelligence or deep learning or prediction model or nomogram or risk score) and (kidney or renal) and transplant* [.mp.; map-to-subject off]	limit to "remove medline records"; not (conference abstract or conference paper or conference review).pt.	269	37
Embase (Ovid)	Graft survival	(kidney or renal) and transplant* and (graft survival or graft failure or graft loss) and (machine learning or artificial intelligence or deep learning or neural network or random forest or random survival forest or prediction model or nomogram) [.mp.]	As above	747	96
Web of Science Core Collection	DGF	TS=("delayed graft function" AND ("machine learning" OR "artificial intelligence" OR "deep learning" OR "prediction model" OR nomogram OR "risk score") AND (kidney OR renal) AND transplant*)	Articles; records already retrieved removed	135	0 new
Web of Science Core Collection	Graft survival	TS=(("kidney transplant*" OR "renal transplant*") AND ("graft survival" OR "graft failure" OR "graft loss" OR "death-censored graft") AND ("machine learning" OR "artificial intelligence" OR "deep learning" OR "neural network" OR "random forest" OR "random survival forest" OR "prediction model" OR nomogram))	As above	252	0 new
Scopus and Semantic Scholar (via Consensus)	Both	Concept summary: Natural-language queries combining kidney transplantation, DGF or graft survival, and machine-learning or prediction-model terms	No limits	Relevance-ranked results	1 (PMID 34448704)
Europe PMC citations	DGF	All records citing Irish 2003, Irish 2010, Jeldres 2009, Chapal 2014 or Zaza 2015 DGF models	No limits	472 (77 already identified)	395

Databases were searched from inception to 28 June 2026. Of the 1,403 Embase and Web of Science records, 1,270 were removed before screening: 399 Embase records indexed in MEDLINE, 484 conference abstracts, 81 records that were not articles and 306 duplicates. The Embase segment covered 1947 to 25 June 2026. Twelve of the PubMed records retained were retrieved by both searches and were screened once, so the last column sums to 614 and 602 records were screened. The PubMed/MEDLINE graft-survival strategy is given by its concept structure. Grey literature and preprint servers were not searched.

Search documentation

The main database searches covered inception to 28 June 2026. Table S1 gives the saved PubMed broad DGF query and the Embase and Web of Science strings. The supplementary PubMed discrimination search, PubMed graft-survival strategy and Consensus searches are presented as concept summaries. Separate last-consulted dates for Consensus and Europe PMC were not recorded.

Database search sequence

The broad PubMed DGF query returned 311 records and was combined with the discrimination-focused set to give 380 unique records. In Embase, the DGF search returned 269 records, 152 after removal of MEDLINE records and 37 after exclusion of conference publications. The corresponding graft-survival counts were 747, 465 and 96. Web of Science returned 135 DGF records and 252 graft-survival records; article filtering retained 106 and 200, respectively, before duplicate removal. The reconciled screening counts are given in Table S1 and Figure 1.

Supplementary searching

Consensus was used for natural-language searches combining kidney transplantation with DGF or graft survival and machine-learning or prediction-model concepts. Europe PMC citation searching retrieved records citing the Irish 2003, Irish 2010, Jeldres 2009, Chapal 2014 and Zaza 2015 models. Citation records were deduplicated against the database searches and screened against the same eligibility criteria.

Table S2. Characteristics and discrimination of delayed graft function reports

Report	PMID	Country	Setting	Recipients	Events	DGF definition	Target	Report type	Model class	Evaluation	AUC	95% CI	Calibration	Overall risk of bias
Brier 2003	14605292	United States	Single center	304	NR	Dialysis within 7 days	Occurrence	Development	Machine learning	Internal	0.668	No	No	High
Irish 2003	14569108	United States	Registry or multicenter	13,846	NR	Dialysis within 7 days	Occurrence	Development and validation	Regression or clinical score	Temporal or geographic	0.700	No	No	High
Avihingsanon 2005	15888558	United States	Single center	75	10	Dialysis within 7 days	Occurrence	Development	Machine learning	Internal	1.000	No	No	High
Grossberg 2006	16436956	United States	Single center	169	42	Dialysis within 7 days	Occurrence	External validation	Not reported	NA	NR	NA	Yes	High
Parikh 2006	16827865	NR	Registry or multicenter	30	10	Dialysis within 7 days	Occurrence	Single marker	Single biomarker	Apparent	0.900	0.710–1.000	No	High
Moore 2007	17556406	United Kingdom	Single center	210	75	Dialysis within 7 days	Occurrence	External validation	Regression or clinical score	External	0.710	0.630–0.780	No	High
Mueller 2008	18021287	Canada	Single center	42	10	Dialysis within 7 days	Occurrence	Development	Not reported	NA	NR	NA	No	High
Jeldres 2009	19829730	Canada	Single center	532	103	Dialysis within 7 days	Occurrence	Development	Regression or clinical score	Internal	0.743	No	Yes	High
Alachkar 2010	21094786	United States	Single center	61	14	Composite	Delayed or slow function	Single marker	Regression or clinical score	Apparent	0.890	0.810–0.970	No	High
Dolegowska 2010	20530808	Poland	Single center	69	45	Composite	Delayed or slow function	Single marker	Single biomarker	Apparent	0.710	No	No	High
Dołęgowska 2010	20829075	Poland	Single center	69	NR	Composite	Delayed or slow function	Single marker	Single biomarker	Apparent	0.700	No	No	High
Hall 2010	19762491	United States	Registry or multicenter	91	34	Dialysis within 7 days	Occurrence	Single marker	Regression or clinical score	Apparent	0.880	No	No	High
Irish 2010	20883559	United States	Registry or multicenter	24,337	NR	Dialysis within 7 days	Occurrence	Development and validation	Regression or clinical score	Temporal or geographic	0.704	No	Yes	High
Kusaka 2010	20525436	Japan	Single center	34	10	Other	Occurrence	Single marker	Not reported	NA	NR	NA	No	High
Moers 2010	20861807	Several countries	Registry or multicenter	306	NR	Dialysis within 7 days	Occurrence	Single marker	Single biomarker	Apparent	0.625	No	No	High
Plata-Munoz 2010	19719466	United Kingdom	Single center	224	NR	Dialysis within 7	Occurrence	External	Not reported	NA	NR	NA	No	High

Report	PMID	Country	Setting	Recipients	Events	DGF definition	Target	Report type	Model class	Evaluation	AUC	95% CI	Calibration	Overall risk of bias
						days		validation						
Bataille 2011	21956199	France	Single center	41	15	Dialysis within 7 days	Occurrence	Single marker	Single biomarker	Apparent	0.970	0.930–1.000	No	High
Jochmans 2011	21834917	Several countries	Registry or multicenter	302	NR	Dialysis within 7 days	Occurrence	Single marker	Single biomarker	Apparent	0.580	No	No	High
Korbély 2011	20819195	Hungary	Single center	34	17	Dialysis within 7 days	Occurrence	Development	Regression or clinical score	Internal	0.750	No	Yes	High
Hollmen 2011a	20861824	Finland	Single center	176	70	Composite	Occurrence	Single marker	Single biomarker	Apparent	0.750	0.663–0.837	No	High
Hollmen 2011b	21545740	Finland	Single center	176	70	Composite	Occurrence	Single marker	Single biomarker	Apparent	0.595	0.506–0.749	No	High
Dołęgowska 2012	22279544	Poland	Single center	69	45	Composite	Delayed or slow function	Single marker	Single biomarker	Apparent	0.830	No	No	High
Rodrigo 2012	22026730	NR	Single center	342	NR	Dialysis within 7 days	Occurrence	External validation	Regression or clinical score	External	0.710	0.653–0.767	Yes	High
Fonseca 2013	24288591	Portugal	Single center	40	18	Dialysis within 7 days	Occurrence	Single marker	Single biomarker	Apparent	0.880	0.770–1.000	No	High
Gourishankar 2013	23731367	Canada	Single center	730	189	Not reported	Occurrence	External validation	Regression or clinical score	External	0.685	No	No	High
Kreepala 2013	23282320	Canada	Single center	70	12	Dialysis within 7 days	Occurrence	Development	Regression or clinical score	Internal	0.870	No	No	High
Schmidt 2013	23291472	United States	Registry or multicenter	78	26	Dialysis within 7 days	Occurrence	Single marker	Single biomarker	Apparent	0.860	No	No	High
Welberry Smith 2013	23739232	United Kingdom	Single center	194	55	Dialysis within 7 days	Occurrence	Single marker	Single biomarker	Temporal or geographic	0.755	Reported	Yes	High
Buemi 2014	25011070	Belgium	Single center	97	20	Dialysis within 7 days	Occurrence	Single marker	Single biomarker	Apparent	0.800	No	No	High
Chapal 2014	24897036	France	Registry or multicenter	1,844	468	Dialysis within 7 days	Occurrence	Development and validation	Regression or clinical score	Internal	0.730	0.680–0.770	Yes	High
Fonseca 2014	24406454	Portugal	Single center	40	18	Dialysis within 7 days	Occurrence	Single marker	Single biomarker	Apparent	0.900	No	No	High
Goncalves-Primo 2014	24503763	Brazil	Single center	72	NR	Dialysis within 7 days	Occurrence	Single marker	Single biomarker	Apparent	0.670	Reported	No	High
Pajek 2014	25027586	Slovenia	Single center	71	40	Composite	Delayed or slow function	Single marker	Single biomarker	Apparent	0.900	0.800–0.960	No	High
Wohlfahrtova 2014	24092381	Czech Republic	Single center	35	9	Dialysis within 7 days	Occurrence	Development	Regression or clinical score	Internal	0.810	No	No	High
Burgos Revilla 2015	25645760	Spain	Single center	90	NR	Creatinine-based	Occurrence	Single marker	Single biomarker	Apparent	0.580	0.440–0.710	No	High
Cantaluppi 2015	26125566	Italy	Single center	50	14	Dialysis within 7 days	Occurrence	Single marker	Single biomarker	Apparent	0.940	No	No	High
Chaumont 2015	26448870	NR	Single center	1,784	382	Other	Occurrence	Development	Regression or clinical score	Apparent	0.774	0.740–0.810	Yes	High
Gómez 2015	25645764	Spain	Single center	93	NR	Creatinine-based	Occurrence	Single marker	Single biomarker	Apparent	0.620	Reported	No	High
Hosgood 2015	26313559	United Kingdom	Single center	36	4	Dialysis within 7 days	Occurrence	Development	Not reported	NA	NR	NA	No	High
Muller 2015	25611655	France	Registry or	243	56	Dialysis within 7	Occurrence	Single marker	Single	Apparent	0.505	Reported	No	High

Report	PMID	Country	Setting	Recipients	Events	DGF definition	Target	Report type	Model class	Evaluation	AUC	95% CI	Calibration	Overall risk of bias
			multicenter			days			biomarker					
Yong 2015	26293021	NR	Single center	66	20	Dialysis within 7 days	Occurrence	Single marker	Not reported	NA	NR	NA	No	High
Zaza 2015	25164408	Italy	Registry or multicenter	2,755	774	Dialysis within 7 days	Occurrence	Development	Regression or clinical score	Apparent	0.660	Reported	No	High
Decruyenaere 2015a	26387917	Belgium	Single center	497	62	Dialysis within 7 days	Occurrence	Development and validation	Regression or clinical score	Apparent	0.822	0.790–0.854	Yes	High
Fonseca 2015a	25458832	Portugal	Single center	40	18	Dialysis within 7 days	Occurrence	Single marker	Single biomarker	Apparent	0.760	0.600–0.920	No	High
Pianta 2015a	25083615	Australia	Single center	81	23	Dialysis within 7 days	Occurrence	Single marker	Regression or clinical score	Apparent	0.810	0.660–0.960	No	High
Decruyenaere 2015b	26466993	Belgium	Single center	497	62	Dialysis within 7 days	Occurrence	Development	Machine learning	Internal	0.843	No	No	High
Fonseca 2015b	26162495	Portugal	Single center	40	18	Dialysis within 7 days	Occurrence	Development	Regression or clinical score	Apparent	0.960	0.905–1.000	No	High
Pianta 2015b	25938452	Australia	Single center	56	22	Dialysis within 7 days	Occurrence	Single marker	Regression or clinical score	Apparent	0.790	Reported	No	High
Pianta 2015c	26174580	Australia	Single center	56	22	Dialysis within 7 days	Occurrence	Single marker	Regression or clinical score	Apparent	0.850	0.720–0.990	No	High
Koo 2016	26986138	South Korea	Registry or multicenter	109	NR	Composite	Delayed or slow function	Development	Regression or clinical score	Internal	0.752	No	Yes	High
Mojtahedzadeh 2016	28855931	Iran	Single center	69	13	Composite	Occurrence	Single marker	Regression or clinical score	Apparent	0.871	0.750–1.000	No	High
Nguyen 2016	26425877	Canada	Single center	76	52	Dialysis within 7 days	Delayed or slow function	Development	Regression or clinical score	Apparent	0.900	No	No	High
Ding 2017	29052563	China	Single center	543	NR	Dialysis within 7 days	Occurrence	Development and validation	Regression or clinical score	Internal	0.783	0.680–0.886	Yes	High
Michalak 2017	29104860	Belgium	Single center	247	38	Dialysis within 7 days	Occurrence	External validation	Regression or clinical score	External	0.540	No	Yes	High
van Balkom 2017	28476951	Netherlands	Registry or multicenter	40	27	Dialysis within 7 days	Occurrence	Development	Regression or clinical score	Apparent	0.890	No	No	High
Ding 2018	30425191	China	Single center	366	58	Dialysis within 7 days	Occurrence	Development and validation	Regression or clinical score	Internal	0.706	0.608–0.811	Yes	High
Kers 2018	29462353	Netherlands	Registry or multicenter	3,333	1,183	Dialysis within 7 days	Occurrence	External validation	Regression or clinical score	External	0.760	No	Yes	High
Le Borgne 2018	28633603	France	Registry or multicenter	1,844	468	Dialysis within 7 days	Occurrence	Single marker	Single biomarker	Apparent	0.620	0.580–0.650	No	High
Maier 2018	29304176	Austria	Single center	170	52	Dialysis within 7 days	Occurrence	Single marker	Single biomarker	Apparent	0.871	No	No	High
McMahon 2018	29034718	United States	Single center	200	94	Dialysis within 7 days	Occurrence	Single marker	Single biomarker	Apparent	0.850	No	No	High
Schmitt 2018	29505681	Germany	Single center	91	19	Composite	Occurrence	Single marker	Regression or clinical score	Apparent	0.838	No	No	High
Sun 2018	29871001	China	Registry or multicenter	383	NR	Composite	Occurrence	Development and validation	Regression or clinical score	Temporal or geographic	0.780	0.610–0.900	No	High
Tai 2018	29407336	China	Single center	446	NR	Dialysis within 7 days	Occurrence	Development	Regression or clinical score	Apparent	0.851	0.799–0.903	No	High
Zhang 2018	29416727	China	Single center	711	125	Dialysis within 7	Occurrence	External	Regression or	External	0.737	0.684–0.790	Yes	High

Report	PMID	Country	Setting	Recipients	Events	DGF definition	Target	Report type	Model class	Evaluation	AUC	95% CI	Calibration	Overall risk of bias
						days		validation	clinical score					
Aceto 2019	30806201	Italy	Single center	125	38	Dialysis within 7 days	Occurrence	Single marker	Single biomarker	Apparent	0.680	No	No	High
Han 2019	30747854	China	Single center	141	NR	Composite	Delayed or slow function	Development and validation	Regression or clinical score	Apparent	0.887	Reported	No	High
Katou 2019	31703290	Germany	Single center	30	9	Dialysis within 7 days	Occurrence	Single marker	Not reported	NA	NR	NA	No	High
Khalid 2019	30837502	United Kingdom	Registry or multicenter	33	13	Dialysis within 7 days	Occurrence	Development and validation	Omics signature	External	0.750	No	No	High
Kim 2019	31847814	South Korea	Single center	291	8	Dialysis within 7 days	Occurrence	Single marker	Single biomarker	Apparent	0.984	No	No	High
Roest 2019	30444806	Netherlands	Single center	40	20	Dialysis within 7 days	Occurrence	Single marker	Single biomarker	Apparent	0.784	No	No	High
Truche 2019	31732205	France	Single center	15	9	Dialysis within 7 days	Occurrence	Single marker	Single biomarker	Apparent	0.980	Reported	No	High
Weissenbacher 2019	31567358	Austria	Single center	68	23	Dialysis within 7 days	Occurrence	Single marker	Single biomarker	Apparent	0.690	0.560–0.810	No	High
Bae 2020	32996170	United States	Registry or multicenter	133,431	NR	Dialysis within 7 days	Occurrence	Development and validation	Regression or clinical score	Temporal or geographic	0.721	0.714–0.727	Yes	Unclear
Chen 2020	33276737	China	Single center	461	78	Dialysis within 7 days	Occurrence	Development and validation	Regression or clinical score	Internal	0.839	Reported	Yes	High
Costa 2020	32027717	Brazil	Registry or multicenter	443	235	Dialysis within 7 days	Occurrence	Development and validation	Machine learning	Internal	0.886	No	No	High
Halpern 2020	33144442	United States	Single center	378	124	Dialysis within 7 days	Occurrence	Development	Regression or clinical score	Internal	0.755	0.704–0.787	No	High
Kawakita 2020	33110142	United States	Registry or multicenter	55,044	13,792	Dialysis within 7 days	Occurrence	Development and validation	Machine learning	Temporal or geographic	0.734	No	Yes	High
Okada 2020	31811409	Japan	Single center	20	3	Other	Severity	Single marker	Single biomarker	Apparent	0.922	No	No	High
Saha-Chaudhuri 2020	32637142	Canada	Single center	163	40	Dialysis within 7 days	Occurrence	Development	Regression or clinical score	Apparent	0.687	No	No	High
Zheng 2020	32338125	China	Single center	333	46	Dialysis within 7 days	Occurrence	Development	Regression or clinical score	Apparent	0.890	0.840–0.940	No	High
Arias-Cabrales 2021	33841865	Spain	Single center	59	21	Dialysis within 7 days	Occurrence	Single marker	Single biomarker	Apparent	0.780	No	No	High
Ietto 2021	34728075	Italy	Single center	247	41	Not reported	Occurrence	External validation	Regression or clinical score	External	0.690	No	No	High
Irish 2021	34386656	Australia and New Zealand	Registry or multicenter	8,971	2,800	Dialysis within 7 days	Occurrence	Development	Regression or clinical score	Apparent	0.760	No	Yes	High
Jen 2021	33560727	United States	Single center	2,089	NR	Dialysis within 7 days	Occurrence	Development	Machine learning	Internal	0.760	No	No	High
Kemmner 2021	34208140	Germany	Single center	239	64	Dialysis within 7 days	Occurrence	Single marker	Regression or clinical score	Apparent	0.813	0.755–0.871	No	High
Konieczny 2021	34830526	NR	Single center	157	NR	Dialysis within 7 days	Occurrence	Development	Machine learning	Internal	0.920	No	No	High
Pan 2021	34876844	China	Single center	211	29	Dialysis within 7 days	Occurrence	Development and validation	Regression or clinical score	Internal	0.746	No	Yes	High
Qiao 2021	34924501	China	Single center	113	20	Composite	Occurrence	Development	Regression or	Apparent	0.888	0.842–0.933	No	High

Report	PMID	Country	Setting	Recipients	Events	DGF definition	Target	Report type	Model class	Evaluation	AUC	95% CI	Calibration	Overall risk of bias
									clinical score					
Xue 2021	33719820	China	Registry or multicenter	1,807	343	Dialysis within 7 days	Donor-level	Development and validation	Regression or clinical score	Temporal or geographic	0.755	0.670–0.840	Yes	High
Yu 2021	33968652	NR	Public dataset	64	53	Dialysis within 7 days	Occurrence	Development	Omics signature	Apparent	0.971	No	Yes	High
Goujon 2022	35945114	France	Single center	115	15	Dialysis within 7 days	Occurrence	Development	Regression or clinical score	Apparent	0.790	0.640–0.940	No	High
Kurian 2022	36406898	United States	Registry or multicenter	12,856	NR	Dialysis within 7 days	Severity	Development	Regression or clinical score	Internal	0.700	No	Yes	High
Li 2022	36035383	China	Single center	517	90	Composite	Occurrence	Development	Single biomarker	Internal	0.828	No	Yes	High
Matas 2022	33675321	United States	Registry or multicenter	896	183	Dialysis within 7 days	Occurrence	External validation	Regression or clinical score	External	0.706	Reported	No	High
Miret Alomar 2022	33887895	Spain	Single center	194	43	Dialysis within 7 days	Occurrence	Development	Regression or clinical score	Apparent	0.820	0.730–0.910	No	High
Scurt 2022	36043436	Germany	Registry or multicenter	223	109	Dialysis within 7 days	Occurrence	Single marker	Regression or clinical score	Apparent	0.779	Reported	Yes	High
Tepel 2022	35260630	Denmark and the Netherlands	Registry or multicenter	257	32	Dialysis within 7 days	Occurrence	Single marker	Single biomarker	Apparent	0.640	0.530–0.760	No	High
Wei 2022	35211472	NR	Public dataset	203	NR	Not reported	Occurrence	Development and validation	Omics signature	External	0.754	No	No	High
Wu 2022	36532016	NR	Public dataset	203	NR	Not reported	Occurrence	Development and validation	Omics signature	External	0.744	No	No	High
Xiao 2022	36372566	China	Single center	84	54	Dialysis within 7 days	Occurrence	Development and validation	Regression or clinical score	Temporal or geographic	0.905	0.738–1.000	Yes	High
Zhang 2022	36346017	China	Single center	199	44	Composite	Occurrence	Single marker	Regression or clinical score	Apparent	0.873	0.823–0.923	No	High
Zhao 2022	35971094	China	Single center	71	23	Dialysis within 7 days	Occurrence	Development and validation	Regression or clinical score	Temporal or geographic	0.879	0.715–0.967	Yes	High
Bi 2023	37275548	NR	Public dataset	202	NR	Not reported	Occurrence	Development and validation	Omics signature	External	0.773	No	No	High
He 2023	37722724	China	Single center	90	25	Dialysis within 7 days	Occurrence	Development and validation	Regression or clinical score	Temporal or geographic	0.840	0.620–1.000	No	High
Li 2023	37965311	NR	Public dataset	203	NR	Not reported	Occurrence	Development	Omics signature	Internal	0.694	No	No	High
Mao 2023	37739236	China	Single center	54	9	Composite	Delayed or slow function	Development	Regression or clinical score	Apparent	0.871	0.720–1.000	No	High
Pilichowska 2023	38137583	Poland	Single center	365	144	Dialysis within 7 days	Occurrence	Single marker	Single biomarker	Apparent	0.622	0.563–0.680	No	High
Quinino 2023	36872507	Brazil	Single center	859	186	Creatinine-based	Delayed or slow function	Development	Machine learning	Internal	0.780	0.710–0.840	No	High
Scurt 2023	37901299	Germany	Registry or multicenter	223	109	Dialysis within 7 days	Occurrence	External validation	Regression or clinical score	External	0.505	Reported	No	High
Song 2023	35603734	China	Single center	89	16	Composite	Occurrence	Development	Regression or clinical score	Apparent	0.735	Reported	No	High
Lin 2023a	37183066	Taiwan	Single center	42	26	Composite	Occurrence	Development	Regression or clinical score	Apparent	0.844	No	No	High
Lin 2023b	37398694	Australia	Registry or multicenter	696	380	Dialysis within 7 days	Occurrence	Development	Machine learning	Internal	0.720	0.680–0.760	No	High

Report	PMID	Country	Setting	Recipients	Events	DGF definition	Target	Report type	Model class	Evaluation	AUC	95% CI	Calibration	Overall risk of bias
Cirillo 2024	38460018	Belgium	Single center	49	19	Creatinine-based	Occurrence	Development	Omics signature	Not reported	0.917	No	No	High
Ernst 2024	38632055	Eurotransplant countries	Registry or multicenter	620	166	Dialysis within 7 days	Occurrence	Development and validation	Regression or clinical score	External	0.702	No	Yes	High
Leite 2024	38743065	Brazil	Registry or multicenter	43	10	Dialysis within 7 days	Occurrence	Single marker	Single biomarker	Apparent	0.766	0.603–0.929	No	High
Maslauskiene 2024	38611630	Lithuania	Single center	76	21	Dialysis within 7 days	Occurrence	Development	Regression or clinical score	Apparent	0.863	No	No	High
Tam 2024	38993786	United Kingdom	Registry or multicenter	143	37	Dialysis within 7 days	Occurrence	Development	Machine learning	Internal	0.642	No	No	High
Tirasattayapitak 2024	39768425	Thailand	Single center	354	64	Dialysis within 7 days	Occurrence	Development	Machine learning	Internal	0.989	0.972–1.000	Yes	High
Wang 2024	38163575	NR	Public dataset	NR	NR	Not reported	Occurrence	Development and validation	Omics signature	External	0.725	No	Yes	High
Li 2024a	38641807	China	Single center	492	96	Dialysis within 7 days	Occurrence	Development and validation	Regression or clinical score	Temporal or geographic	0.870	0.800–0.940	Yes	High
Li 2024b	39188701	China	Single center	50	12	Creatinine-based	Occurrence	Single marker	Single biomarker	Apparent	0.908	No	No	High
Mao 2024b	39549926	NR	Public dataset	203	NR	Dialysis within 7 days	Occurrence	Development and validation	Omics signature	External	0.705	No	Yes	High
Benning 2025	40475974	Germany	Single center	159	53	Dialysis within 7 days	Occurrence	Single marker	Single biomarker	External	0.880	0.750–1.000	No	High
Hu 2025	40770395	NR	Public dataset	202	NR	Not reported	Occurrence	Development and validation	Omics signature	External	0.706	No	No	High
Ma 2025	40165952	China	Single center	108	48	Composite	Occurrence	Development	Regression or clinical score	Apparent	0.832	0.756–0.908	No	High
Wrigge 2025	39891083	Germany	Single center	70	18	Dialysis within 7 days	Occurrence	Single marker	Single biomarker	Apparent	0.967	No	No	High
Yu 2025	41350284	China	Single center	150	45	Creatinine-based	Occurrence	Single marker	Single biomarker	External	0.864	No	No	High
Wu 2025a	40086342	NR	Public dataset	NR	NR	Not reported	Occurrence	Development and validation	Machine learning	External	0.845	No	Yes	High
Banks 2026	41541788	United Kingdom	Registry or multicenter	237	70	Dialysis within 7 days	Occurrence	Development and validation	Regression or clinical score	External	0.805	Reported	Yes	High
Feng 2026	42001037	China	Single center	140	67	Dialysis within 7 days	Occurrence	Development and validation	Regression or clinical score	Temporal or geographic	0.839	0.795–0.883	Yes	High
He 2026	41969322	China	Single center	670	146	Creatinine-based	Occurrence	Development	Machine learning	Internal	0.848	0.798–0.899	No	High
Ma 2026	42417841	China	Single center	43	20	Composite	Delayed or slow function	Development	Regression or clinical score	Internal	0.960	No	No	High
Nica 2026	41869992	United States	Single center	2,725	1,549	Dialysis within 7 days	Occurrence	Development and validation	Machine learning	Internal	0.810	No	Yes	High
Potluri 2026	41135690	United States	Registry or multicenter	NR	NR	Dialysis within 7 days	Occurrence	Development	Regression or clinical score	Internal	0.750	No	Yes	High
Ramalhete 2026	41977063	Portugal	Single center	56	14	Dialysis within 7 days	Occurrence	Development	Machine learning	Internal	0.796	No	Yes	High
Ribeiro Neto 2026	42054170	Brazil	Single center	27	9	Other	Severity	Single marker	Single biomarker	Apparent	0.746	0.534–0.897	No	High
Salgado 2026	41884255	Chile	Registry or	523	NR	Dialysis within 7	Occurrence	Development	Machine	Internal	0.620	No	No	High

Report	PMID	Country	Setting	Recipients	Events	DGF definition	Target	Report type	Model class	Evaluation	AUC	95% CI	Calibration	Overall risk of bias
			multicenter			days			learning					
Yan 2026	41782890	China	Single center	36	18	Composite	Occurrence	Development	Regression or clinical score	Apparent	0.932	0.855–1.000	No	High
Zhang 2026	42283098	NR	Public dataset	202	91	Not reported	Occurrence	Development	Machine learning	Internal	0.871	No	Yes	High
Zhu 2026	42216373	China	Single center	280	88	Dialysis within 7 days	Occurrence	Development	Regression or clinical score	Apparent	0.923	0.888–0.957	No	High
Dong 2026a	41736560	China	Single center	2,041	264	Dialysis within 7 days	Occurrence	Development	Regression or clinical score	Internal	0.780	0.760–0.820	Yes	High
Dong 2026b	41947228	NR	Public dataset	NR	NR	Dialysis within 7 days	Occurrence	Development and validation	Machine learning	External	0.700	No	No	High

One estimate per report: the final model in its most independent evaluation; the median when several estimates remained at that level. 95% CI: the interval of the estimate taken; “Reported” where an interval was reported but the estimate taken is the median of several models; “No” where none was reported. A letter after the year distinguishes reports whose first authors share a surname and a year of publication, in order of PubMed identifier. NR, not reported; NA, not applicable; CI, confidence interval.

Table S3. External validations of named models and sensitivity analyses

Model	Outcome	Cohort	PMID	Recipients	Events	AUC or C-statistic	95% CI	Interval	Sample taken
Irish 2010	DGF	Rodrigo 2012	22026730	342	NR	0.710	0.653–0.767	Reported	Only estimate reported
Irish 2010	DGF	Kreepala 2013	23282320	68	12	0.680	Not reported	Derived from events	Largest sample
Irish 2010	DGF	Gourishankar 2013	23731367	730	189	0.680	Not reported	Derived from events	Value reported for the 2010 version
Irish 2010	DGF	Pianta 2015b	25938452	56	22	0.720	0.580–0.860	Reported	Largest sample
Irish 2010	DGF	Decruyenaere 2015a	26387917	497	62	0.779	0.745–0.814	Reported	Only estimate reported
Irish 2010	DGF	van Balkom 2017	28476951	40	27	0.590	Not reported	Derived from events	Only estimate reported
Irish 2010	DGF	Michalak 2017	29104860	247	38	0.690	Not reported	Derived from events	Only estimate reported
Irish 2010	DGF	Zhang 2018	29416727	711	125	0.737	0.684–0.790	Reported	Only estimate reported
Irish 2010	DGF	Kers 2018	29462353	3,166	1,148	0.758	Not reported	Derived from events	Sample meeting the model's own eligibility criteria
Irish 2010	DGF	Sun 2018	29871001	38	5	0.750	0.590–0.880	Reported	Only estimate reported
Irish 2010	DGF	Khalid 2019	30837502	33	13	0.710	Not reported	Derived from events	Only estimate reported
Irish 2010	DGF	Costa 2020	32027717	443	235	0.685	Not reported	Derived from events	Only estimate reported
Irish 2010	DGF	Matas 2022	33675321	478	116	0.701	0.646–0.756	Reported	Sample meeting the model's own eligibility criteria
Irish 2010	DGF	Scurt 2023	37901299	223	109	0.684	0.612–0.757	Reported	Only estimate reported
Irish 2003	DGF	Moore 2007	17556406	210	75	0.710	0.630–0.780	Reported	Only estimate reported
Irish 2003	DGF	Gourishankar 2013	23731367	730	189	0.680	Not reported	Derived from events	Only estimate reported
Irish 2003	DGF	Zhang 2018	29416727	711	125	0.676	0.619–0.733	Reported	Only estimate reported

Model	Outcome	Cohort	PMID	Recipients	Events	AUC or C-statistic	95% CI	Interval	Sample taken
Irish 2003	DGF	Miret Alomar 2022	33887895	194	43	0.760	0.670–0.850	Reported	Score not calculated for pre-emptive recipients (13 of 202); number evaluated not stated
Irish 2003	DGF	Ietto 2021	34728075	155	25	0.690	Not reported	Derived from events	Only estimate reported
Chapal	DGF	Decruyenaere 2015a	26387917	497	62	0.596	0.558–0.635	Reported	Only estimate reported
Chapal	DGF	Michalak 2017	29104860	247	38	0.510	Not reported	Derived from events	Only estimate reported
Chapal	DGF	Zhang 2018	29416727	711	125	0.664	0.603–0.724	Reported	Only estimate reported
Chapal	DGF	Kers 2018	29462353	1,362	292	0.617	Not reported	Derived from events	Sample meeting the model's own eligibility criteria
Chapal	DGF	Ietto 2021	34728075	41	7	0.570	Not reported	Derived from events	Only estimate reported
Chapal	DGF	Zhao 2022	35971094	71	23	0.720	0.601–0.820	Reported	Largest sample
Chapal	DGF	Scurt 2023	37901299	131	NR	0.709	0.617–0.801	Reported	Only estimate reported
Chapal	DGF	Ernst 2024	38632055	620	166	0.596	Not reported	Derived from events	Largest sample
Jeldres	DGF	Chapal 2014	24897036	454	NR	0.580	0.520–0.730	Reported	Largest sample
Jeldres	DGF	Decruyenaere 2015a	26387917	497	62	0.654	0.615–0.692	Reported	Only estimate reported
Jeldres	DGF	Michalak 2017	29104860	247	38	0.540	Not reported	Derived from events	Only estimate reported
Jeldres	DGF	Zhang 2018	29416727	711	125	0.504	0.442–0.566	Reported	Only estimate reported
Jeldres	DGF	Kers 2018	29462353	3,333	1,183	0.565	Not reported	Derived from events	Only estimate reported
Jeldres	DGF	Ietto 2021	34728075	235	41	0.600	Not reported	Derived from events	Only estimate reported
Jeldres	DGF	Scurt 2023	37901299	223	109	0.503	0.423–0.582	Reported	Only estimate reported
Zaza	DGF	Decruyenaere 2015a	26387917	497	62	0.669	0.631–0.707	Reported	Only estimate reported
Zaza	DGF	Zhang 2018	29416727	711	125	0.579	0.521–0.638	Reported	Only estimate reported
Zaza	DGF	Kers 2018	29462353	2,268	801	0.546	Not reported	Derived from events	Sample meeting the model's own eligibility criteria
KDPI/KDRI	DGF	Kreepala 2013	23282320	70	12	0.710	Not reported	Derived from events	Largest sample
KDPI/KDRI	DGF	van Balkom 2017	28476951	40	27	0.550	Not reported	Derived from events	Only estimate reported
KDPI/KDRI	DGF	Weissenbacher 2019	31567358	68	23	0.620	Not reported	Derived from events	Value given in the authors' reply to discussants; sample of the main analysis assumed
KDPI/KDRI	DGF	Zhao 2022	35971094	71	23	0.764	0.649–0.857	Reported	Largest sample
KDPI/KDRI	DGF	Xiao 2022	36372566	68	45	0.660	0.527–0.794	Reported	Largest sample
KDPI/KDRI	DGF	He 2023	37722724	60	21	0.740	0.600–0.890	Reported	Only estimate reported
KDPI/KDRI	DGF	Ma 2025	40165952	108	48	0.678	0.575–0.782	Reported	Only estimate reported
Schold	DGF	Kreepala 2013	23282320	70	12	0.690	Not reported	Derived from events	Largest sample
Schold	DGF	Scurt 2023	37901299	222	NR	0.451	0.373–0.530	Reported	Only estimate reported
Balaz	DGF	Scurt 2023	37901299	171	NR	0.506	0.417–0.595	Reported	Largest sample; first of two of equal size

Model	Outcome	Cohort	PMID	Recipients	Events	AUC or C-statistic	95% CI	Interval	Sample taken
Balaz	DGF	Ernst 2024	38632055	620	166	0.512	Not reported	Derived from events	Largest sample
Remuzzi	DGF	Weissenbacher 2019	31567358	68	23	0.610	0.460–0.760	Reported	Only estimate reported
Remuzzi	DGF	Zheng 2020	32338125	333	46	0.650	0.550–0.740	Reported	Only estimate reported
Remuzzi	DGF	Ma 2025	40165952	108	48	0.604	0.494–0.715	Reported	Only estimate reported

Model	Analysis	Cohorts	Pooled AUC	95% CI	95% prediction interval	I^2	τ^2 (logit)	Q (P)
Chapal	Cohorts with a reported interval	4	0.658	0.553–0.750	0.389–0.853	65%	0.047	8.68 (P = 0.034)
Chapal	Adding cohorts with an interval derived from events	8	0.619	0.570–0.666	0.521–0.708	51%	0.019	14.40 (P = 0.044)
Chapal	Omitting Decruyenaere 2015a	3	0.683	0.573–0.776	—	—	—	—
Chapal	Omitting Zhang 2018	3	0.664	0.444–0.831	—	—	—	—
Chapal	Omitting Zhao 2022	3	0.646	0.494–0.773	—	—	—	—
Chapal	Omitting Scurt 2023	3	0.645	0.489–0.775	—	—	—	—
Irish 2003	Cohorts with a reported interval	3	0.700	0.598–0.786	0.352–0.909	6%	0.002	2.12 (P = 0.346)
Irish 2003	Adding cohorts with an interval derived from events	5	0.690	0.646–0.731	0.640–0.737	0%	0.000	2.49 (P = 0.647)
Irish 2010	Cohorts with a reported interval	7	0.729	0.689–0.766	0.646–0.799	39%	0.016	9.84 (P = 0.132)
Irish 2010	Adding cohorts with an interval derived from events	14	0.717	0.688–0.745	0.635–0.787	57%	0.026	30.09 (P = 0.005)
Irish 2010	Omitting Rodrigo 2012	6	0.733	0.682–0.778	—	—	—	—
Irish 2010	Omitting Pianta 2015b	6	0.729	0.682–0.771	—	—	—	—
Irish 2010	Omitting Decruyenaere 2015a	6	0.712	0.673–0.748	—	—	—	—
Irish 2010	Omitting Zhang 2018	6	0.726	0.672–0.775	—	—	—	—
Irish 2010	Omitting Sun 2018	6	0.728	0.681–0.770	—	—	—	—
Irish 2010	Omitting Matas 2022	6	0.736	0.688–0.778	—	—	—	—
Irish 2010	Omitting Scurt 2023	6	0.737	0.693–0.776	—	—	—	—
Jeldres	Cohorts with a reported interval	4	0.563	0.413–0.702	0.190–0.876	86%	0.120	21.96 (P < 0.001)
Jeldres	Adding cohorts with an interval derived from events	7	0.567	0.509–0.623	0.425–0.699	76%	0.040	24.54 (P < 0.001)
Jeldres	Omitting Chapal 2014	3	0.558	0.315–0.777	—	—	—	—
Jeldres	Omitting Decruyenaere 2015a	3	0.516	0.417–0.613	—	—	—	—
Jeldres	Omitting Zhang 2018	3	0.584	0.355–0.782	—	—	—	—
Jeldres	Omitting Scurt 2023	3	0.582	0.344–0.787	—	—	—	—

Random-effects model on the logit scale; DerSimonian–Laird between-study variance; Hartung–Knapp–Sidik–Jonkman adjustment with the variance not below the conventional estimate. Where a report gave the same model in several samples, the sample to which the model's own eligibility criteria had been applied was taken, and otherwise the largest sample (last column). For Zhang 2018 the values of four models are those printed in a figure of the report, whose text describes them as below 0.6. Standard errors derived from events use the Hanley–McNeil method.

Table S4. Characteristics and discrimination of graft-survival reports

Report	PMID	Country	Setting	Recipients	Events	Outcome	Prediction time	Report type	Model class	Evaluation	C-statistic or AUC	Horizon	1 y	3 y	5 y	10 y	Interval reported	Calibration	Overall risk of bias
Tiong 2009	19167732	United States	Registry or multicenter	20,085	2,300	Not reported	Before or at transplantation	Development	Regression or clinical score	Internal	0.747	5 y			0.747		No	Yes	Unclear
Greco 2010	20534243	NR	Not reported	194	NR	Not reported	Not reported	Development	Not reported	NA	NR	NA					NA	No	High

Report	PMID	Country	Setting	Recipients	Events	Outcome	Prediction time	Report type	Model class	Evaluation	C-statistic or AUC	Horizon	1 y	3 y	5 y	10 y	Interval reported	Calibration	Overall risk of bias
Kasiske 2010	20801565	United States	Registry or multicenter	59,091	NR	All-cause graft loss	Before or at transplantation	Development and validation	Regression or clinical score	Internal	0.674	5 y			0.674		No	Yes	High
Plata-Munoz 2010	19719466	United Kingdom	Single center	224	NR	Death-censored graft failure	Before or at transplantation	External validation	Not reported	NA	NR	NA					NA	No	High
Tang 2011	21701272	United States	Registry or multicenter	3,313	NR	Not reported	Before or at transplantation	Development	Regression or clinical score	Internal	0.740	3 y		0.740			Yes	No	High
Brown 2012 †	23221105	United States	Registry or multicenter	5,144	NR	Not reported	Before or at transplantation	Development and validation	Machine learning	External	0.630	Overall	0.590	0.600			No	No	Unclear
Gourishankar 2013	23731367	Canada	Single center	730	145	Death-censored graft failure	Before or at transplantation	External validation	Not reported	NA	NR	NA					NA	No	High
Ho 2013	23192155	Canada	Single center	231	20	Death-censored graft failure	Posttransplant landmark	Development	Regression or clinical score	Apparent	0.890	Overall					No	No	High
Hamidi 2016	27057518	Iran	Registry or multicenter	378	37	Other	Before or at transplantation	Development	Machine learning	Internal	0.965	Overall					No	No	High
Hassanain 2016	27569934	Canada	Registry or multicenter	473	NR	Death-censored graft failure	Posttransplant landmark	Development and validation	Regression or clinical score	Apparent	0.610	Overall					No	Yes	High
Martínez-Mier 2017	28591501	Mexico	Registry or multicenter	109	NR	Death-censored graft failure	Dynamic	External validation	Regression or clinical score	External	0.540	5 y			0.540		Yes	Yes	High
Molnar 2017	28270930	Canada	Registry or multicenter	315	87	Death-censored graft failure	Posttransplant landmark	Development	Regression or clinical score	Internal	0.810	Overall					Yes	Yes	High
Prémaud 2017	28671951	France	Registry or multicenter	664	69	Death-censored graft failure	Dynamic	Development and validation	Machine learning	External	0.790	10 y				0.790	Yes	Yes	High
Yoo 2017	28827646	South Korea	Registry or multicenter	3,117	304	Not reported	Before or at transplantation	Development	Machine learning	Internal	0.800	Overall					No	No	High
Le Borgne 2018	28633603	France	Registry or multicenter	5,158	678	Death-censored graft failure	Posttransplant landmark	Single marker	Single biomarker	Apparent	0.660	10 y				0.660	Yes	No	High
Peters-Sengers 2018a	28731905	Netherlands	Registry or multicenter	3,201	NR	All-cause graft loss	Before or at transplantation	External validation	Regression or clinical score	External	0.630	5 y			0.630		Yes	Yes	Low
Hu 2019	31722677	China	Single center	372	58	All-cause graft loss	Posttransplant landmark	Development	Regression or clinical score	Apparent	0.850	3 y		0.850			No	No	High
Kusaka 2019	30460731	Japan	Single center	315	NR	Not reported	Before or at transplantation	Model update	Regression or clinical score	Apparent	0.731	Overall					Yes	No	High
Stamenic 2019	31093367	France	Single center	616	68	Death-censored graft failure	Posttransplant landmark	Development and validation	Not reported	NA	NR	NA					NA	Yes	High
Vinson 2019	30656212	United States	Registry or multicenter	77,827	23,262	All-cause graft loss	Before or at transplantation	Development and validation	Regression or clinical score	Internal	0.629	Overall					Yes	Yes	High
Bae 2020	32996170	United States	Registry or multicenter	133,431	NR	Death-censored graft failure	Before or at transplantation	Development and validation	Regression or clinical score	Temporal or geographic	0.635	Overall					Yes	Yes	Unclear
Chu 2020	33319199	Several countries	Registry or multicenter	2,889	252	Death-censored graft failure	Posttransplant landmark	External validation	Regression or clinical score	External	0.810	5 y			0.810		Yes	Yes	High
Mottola 2020	33125000	France	Registry or multicenter	754	142	Death-censored graft failure	Posttransplant landmark	Single marker	Single biomarker	External	0.693	Overall					Yes	No	High
Saha-Chaudhuri 2020	32637142	Canada	Single center	163	50	All-cause graft loss	Dynamic	Development	Regression or clinical score	Apparent	0.710	Overall					No	No	High
Zhang 2020	33376751	United States	Public dataset	282	51	Not reported	Posttransplant landmark	Development and validation	Omics signature	Internal	0.763	3 y	0.805	0.763			No	No	High
Irish 2021	34386656	Australia and New Zealand	Registry or multicenter	8,971	2,307	All-cause graft loss	Before or at transplantation	Development	Not reported	NA	NR	NA					NA	Yes	High
Naqvi 2021	34448704	United States	Registry or multicenter	52,827	7,554	All-cause graft loss	Before or at transplantation	Development	Machine learning	Internal	0.690	5 y	0.820		0.690		No	No	High

Report	PMID	Country	Setting	Recipients	Events	Outcome	Prediction time	Report type	Model class	Evaluation	C-statistic or AUC	Horizon	1 y	3 y	5 y	10 y	Interval reported	Calibration	Overall risk of bias
Raynaud 2021	34756569	France	Registry or multicenter	13,608	1,893	Death-censored graft failure	Dynamic	Development and validation	Regression or clinical score	External	0.851	Overall					Yes	Yes	High
Senanayake 2021	34154541	Australia	Registry or multicenter	7,365	693	Death-censored graft failure	Before or at transplantation	Development and validation	Regression or clinical score	Internal	0.670	Overall					No	Yes	High
Divard 2022	36418380	France	Registry or multicenter	400	84	Death-censored graft failure	Posttransplant landmark	Other	Regression or clinical score	Apparent	0.789	7 y					No	Yes	High
Dou 2022	35281025	NR	Public dataset	NR	NR	Not reported	Not reported	Development	Omics signature	Apparent	0.793	3 y	0.804	0.793			No	No	High
Jeong 2022	35610279	South Korea	Registry or multicenter	4,854	NR	Death-censored graft failure	Before or at transplantation	Development	Regression or clinical score	Apparent	0.671	Overall					No	No	High
Kurian 2022	36406898	United States	Registry or multicenter	NR	22,778	All-cause graft loss	Posttransplant landmark	Development	Regression or clinical score	Apparent	0.650	Overall					No	Yes	High
Lu 2022	36393969	NR	Public dataset	NR	NR	Not reported	Not reported	Single marker	Not reported	NA	NR	NA					NA	No	High
Pang 2022	36483557	NR	Public dataset	105	30	Not reported	Posttransplant landmark	Development and validation	Omics signature	Internal	0.830	5 y		0.790	0.830	0.760	No	No	High
Paquette 2022	35700006	United States	Registry or multicenter	180,141	NR	Death-censored graft failure	Before or at transplantation	Development	Machine learning	Internal	0.659	Overall					No	Yes	High
Roller 2022	36388342	Germany	Single center	1,516	NR	Death-censored graft failure	Dynamic	Development	Machine learning	Internal	0.953	1 y	0.953				Yes	No	High
Wu 2022	36532016	NR	Public dataset	NR	NR	Not reported	Not reported	Development and validation	Omics signature	Internal	0.720	2 y	0.767				No	No	High
Yi 2022	34757124	United States and Australia	Registry or multicenter	404	49	Death-censored graft failure	Posttransplant landmark	Development and validation	Not reported	NA	NR	NA					NA	No	High
Badrouchi 2023	38042904	Tunisia	Single center	407	NR	Death-censored graft failure	Posttransplant landmark	Development	Machine learning	Internal	0.897	5 y			0.897		No	No	High
Bi 2023	37275548	NR	Public dataset	206	51	Not reported	Posttransplant landmark	Development and validation	Omics signature	Internal	0.735	3 y	0.770	0.735			No	No	High
Dasariraju 2023	37120046	United States	Registry or multicenter	166,574	NR	Not reported	Before or at transplantation	Development	Not reported	NA	NR	NA					NA	No	High
Mulugeta 2023	37217892	Ethiopia	Single center	278	21	Not reported	Not reported	Development	Not reported	NA	NR	NA					NA	Yes	High
Sandal 2023	37211638	Canada	Single center	792	NR	Death-censored graft failure	Posttransplant landmark	Development and validation	Not reported	NA	NR	NA					NA	No	High
Scurt 2023	37901299	Germany	Registry or multicenter	223	49	Death-censored graft failure	Before or at transplantation	External validation	Regression or clinical score	External	0.617	5 y	0.681	0.604	0.617		Yes	No	High
Truchot 2023	36572246	France	Registry or multicenter	8,422	1,081	Death-censored graft failure	Posttransplant landmark	Development and validation	Machine learning	External	0.833	7 y					Yes	Yes	High
Achilonu 2024	39290362	South Africa	Single center	1,738	NR	Death-censored graft failure	Before or at transplantation	Development and validation	Machine learning	Temporal or geographic	0.585	10 y				0.585	No	No	High
Denic 2024	38881746	United States	Single center	835	58	Death-censored graft failure	Posttransplant landmark	Development	Regression or clinical score	Internal	0.854	5 y			0.854		No	No	High
Ernst 2024	38632055	Eurotransplant countries	Registry or multicenter	711	61	Death-censored graft failure	Before or at transplantation	Development and validation	Regression or clinical score	External	0.733	1 y	0.733				No	Yes	High
Fabreti-Oliveira 2024	38797338	Brazil	Single center	627	64	Other	Posttransplant landmark	Development	Machine learning	Internal	0.840	0.25 y					No	No	High
Ji 2024	39664334	NR	Public dataset	282	NR	Not reported	Not reported	Development	Omics signature	Apparent	0.923	4 y	0.910	0.923			No	No	High
Leite 2024	38743065	Brazil	Registry or multicenter	43	6	Other	Before or at transplantation	Single marker	Single biomarker	Apparent	0.840	0.25 y					Yes	No	High

Report	PMID	Country	Setting	Recipients	Events	Outcome	Prediction time	Report type	Model class	Evaluation	C-statistic or AUC	Horizon	1 y	3 y	5 y	10 y	Interval reported	Calibration	Overall risk of bias
Salaün 2024	39075081	United Kingdom	Registry or multicenter	36,653	NR	Death-censored graft failure	Before or at transplantation	Development	Machine learning	Internal	0.627	Overall	0.622		0.626	0.684	No	Yes	High
Scurt 2024	39047699	Germany	Registry or multicenter	196	26	Death-censored graft failure	Not reported	External validation	Not reported	NA	NR	NA					NA	No	High
Shen 2024	39602449	NR	Public dataset	282	NR	Not reported	Posttransplant landmark	Development	Omics signature	Internal	0.881	3 y	0.816	0.881			No	No	High
Swolinsky 2024	37858309	Germany	Single center	709	49	Death-censored graft failure	Posttransplant landmark	Single marker	Regression or clinical score	Internal	0.844	Overall					Yes	No	High
Wang 2024	38163575	NR	Public dataset	NR	NR	Not reported	Not reported	Development and validation	Omics signature	Apparent	0.875	5 y		0.818	0.875	0.740	No	Yes	High
Yi 2024	37923131	United States	Registry or multicenter	1,560	NR	Death-censored graft failure	Before or at transplantation	Development and validation	Regression or clinical score	Internal	0.670	2 y	0.700				No	No	High
Yu 2024	38184216	China	Single center	353	NR	All-cause graft loss	Before or at transplantation	External validation	Regression or clinical score	External	0.728	3 y		0.728			No	No	High
Zhang 2024	38495888	NR	Public dataset	282	NR	Not reported	Not reported	Single marker	Single biomarker	Apparent	0.723	Overall					No	No	High
Ali 2024a	38552178	United States	Registry or multicenter	156,749	NR	Death-censored graft failure	Before or at transplantation	Development and validation	Machine learning	Internal	0.660	6 y	0.660				No	Yes	High
Mao 2024a	39243908	NR	Public dataset	282	NR	Not reported	Posttransplant landmark	Development	Omics signature	Not reported	0.710	Overall	0.700	0.660			No	No	High
Ali 2024b	38684469	United States	Registry or multicenter	66,914	NR	Death-censored graft failure	Before or at transplantation	Development	Machine learning	Internal	0.700	5 y			0.700	0.675	No	Yes	High
Mao 2024b	39549926	NR	Public dataset	282	NR	Not reported	Not reported	Development and validation	Omics signature	Internal	0.670	2 y	0.660				No	Yes	High
Ali 2024c	39437817	United Kingdom	Registry or multicenter	29,713	NR	All-cause graft loss	Before or at transplantation	Development	Machine learning	Internal	0.740	Overall	0.720	0.740	0.750	0.730	No	Yes	High
Ali 2025	39838510	United Kingdom	Registry or multicenter	12,661	NR	Death-censored graft failure	Before or at transplantation	Development	Machine learning	Internal	0.720	Overall		0.730		0.750	No	Yes	High
Fan 2025	41254094	Switzerland	Registry or multicenter	4,728	NR	Not reported	Dynamic	Development	Machine learning	Internal	0.790	1 y	0.790				Yes	No	High
Gosset 2025	40686676	Several countries	Registry or multicenter	7,040	1,113	Death-censored graft failure	Before or at transplantation	Development and validation	Regression or clinical score	Temporal or geographic	0.620	5 y	0.660	0.630	0.620	0.600	No	Yes	High
He 2025	40236452	China	Single center	508	87	All-cause graft loss	Before or at transplantation	Development	Regression or clinical score	Internal	0.827	Overall	0.985		0.816	0.853	No	Yes	High
Hu 2025	40770395	NR	Public dataset	282	NR	Not reported	Posttransplant landmark	Development and validation	Omics signature	Internal	0.905	4 y	0.861	0.871			No	No	High
Labriffe 2025	41126939	Belgium and France	Registry or multicenter	1,237	222	Death-censored graft failure	Posttransplant landmark	External validation	Machine learning	External	0.760	5 y			0.760	0.730	Yes	No	High
Milecki 2025	40413148	NR	Single center	108	NR	Death-censored graft failure	Posttransplant landmark	Development and validation	Machine learning	External	0.727	5 y			0.727		No	No	High
Mulugeta 2025	39901148	Ethiopia	Single center	278	21	Death-censored graft failure	Before or at transplantation	Development	Regression or clinical score	Internal	0.911	Overall					No	Yes	High
Qiu 2025	41080156	NR	Public dataset	NR	NR	Not reported	Not reported	Development	Omics signature	Internal	0.780	3 y	0.830	0.780			No	Yes	High
Salybekov 2025	41113995	United States	Registry or multicenter	NR	NR	Not reported	Dynamic	Development	Machine learning	Internal	0.700	6 y					No	No	High
Shabani 2025	41068878	Iran	Registry or multicenter	558	40	Other	Dynamic	Development	Machine learning	Internal	0.790	10 y				0.790	No	Yes	High
Shen 2025	41217598	NR	Public dataset	NR	NR	Not reported	Not reported	Development	Machine learning	Internal	0.797	3 y	0.883	0.797			No	No	High
Yin 2025	40521169	China	Single center	2,575	113	Death-censored graft	Posttransplant landmark	Development	Regression or clinical score	Internal	0.868	10 y				0.868	Yes	Yes	High

Report	PMID	Country	Setting	Recipients	Events	Outcome	Prediction time	Report type	Model class	Evaluation	C-statistic or AUC	Horizon	1 y	3 y	5 y	10 y	Interval reported	Calibration	Overall risk of bias
						failure													
Wu 2025a	40086342	NR	Public dataset	NR	NR	Not reported	Not reported	Development and validation	Machine learning	Internal	0.710	Overall	0.896	0.853			No	Yes	High
Wu 2025b	40624201	NR	Public dataset	NR	51	Not reported	Posttransplant landmark	Development	Machine learning	Internal	0.740	Overall	0.888	0.890			No	No	High
Apanisile 2026	42361104	United States	Registry or multicenter	228,855	55,346	Death-censored graft failure	Before or at transplantation	Development	Regression or clinical score	Internal	0.687	Overall	0.692	0.688	0.699	0.746	No	No	High
Banks 2026	41541788	United Kingdom	Registry or multicenter	237	64	Death-censored graft failure	Posttransplant landmark	Development and validation	Regression or clinical score	Internal	0.535	Overall					Yes	Yes	High
Filho 2026	41580479	Canada and United States	Public dataset	282	74	Death-censored graft failure	Posttransplant landmark	Development	Machine learning	Internal	0.870	Overall	0.772	0.954			Yes	Yes	High
Gao 2026	42272126	China	Single center	940	171	All-cause graft loss	Posttransplant landmark	Development	Machine learning	Internal	0.859	5 y			0.859		Yes	Yes	High
Garcia-Lopez 2026	42088642	Colombia	Registry or multicenter	2,030	186	Death-censored graft failure	Before or at transplantation	Development	Machine learning	Internal	0.870	1 y	0.870				No	Yes	High
Potluri 2026	41135690	United States	Registry or multicenter	75,867	9,509	All-cause graft loss	Before or at transplantation	Development	Regression or clinical score	Internal	0.670	5 y		0.678	0.670		No	Yes	High
Shehab 2026	41615413	Netherlands	Single center	78	29	Death-censored graft failure	Posttransplant landmark	Development	Machine learning	Internal	0.878	Overall					Yes	Yes	High
Shen 2026	42270478	China	Single center	356	89	Not reported	Before or at transplantation	Development and validation	Machine learning	Internal	0.714	1 y	0.714				Yes	Yes	High
Zhang 2026	42283098	NR	Public dataset	206	51	Not reported	Posttransplant landmark	Development	Omics signature	Internal	0.683	3 y	0.714	0.683			No	Yes	High

One estimate per report as for Table S2; the overall C-statistic where given, otherwise the horizon closest to five years. Horizon columns give the final model at that horizon where reported. † Full text could not be obtained; data and risk-of-bias answers are from the published abstract. The interval indicator refers to the selected main estimate or its component estimates; uncertainty for additional horizons is listed below.

Table S4 continued Reported uncertainty for graft survival estimates

Individual model and cohort estimates are shown below with their reported uncertainty. “Component” identifies an estimate contributing to a median in Table S4; its interval applies to that estimate, not to the median. “Selected” identifies an individual estimate used directly in Table S4. Additional horizon-specific estimates retain their own intervals. Where no numerical interval was reported, none is assigned. CI, confidence interval; CrI, credible interval; SE, standard error. All values are transcribed from the source reports.

Report and PMID	Model and cohort	Metric	Horizon years	Evaluation	Estimate	Reported uncertainty	Relation to Table S4
Tang 2011<br>PMID 21701272	Logistic regression, 38 predictors	AUC	3	cross validation	0.74	95% CI 0.72–0.77	Selected
Martínez-Mier 2017<br>PMID 28591501	Kasiske tool applied at time of transplant	C-statistic	5	external	0.54	95% CI 0.423–0.657	Component
Martínez-Mier 2017<br>PMID 28591501	Kasiske tool applied 7 days after transplant	C-statistic	5	external	0.517	95% CI 0.399–0.635	Component

Report and PMID	Model and cohort	Metric	Horizon years	Evaluation	Estimate	Reported uncertainty	Relation to Table S4
Martínez-Mier 2017 PMID 28591501	Kasiske tool applied 1 year after transplant	C-statistic	5	external	0.682	95% CI 0.524–0.841	Component
Molnar 2017 PMID 28270930	Post-AKI graft-loss risk score, bootstrap internal validation	C-statistic	Overall	bootstrap	0.81	95% CrI 0.79–0.82	Selected
Prémaud 2017 PMID 28671951	AdGFS, external validation (Tours and Poitiers)	Time-dependent AUC	10	external	0.79	95% CI 0.74–0.84	Selected
Le Borgne 2018 PMID 28633603	12-month serum creatinine, standardized and weighted (IPW) time-dependent ROC	Time-dependent AUC	10	apparent	0.66	95% CI 0.63–0.7	Selected
Peters-Sengers 2018a PMID 28731905	KDRIfull (Rao 2009), all-cause graft failure (death or return to dialysis)	C-statistic	5	external	0.63	95% CI 0.62–0.64	Component
Peters-Sengers 2018a PMID 28731905	KDRIfull (Rao 2009), death-censored graft survival	C-statistic	5	external	0.63	95% CI 0.61–0.65	Component
Kusaka 2019 PMID 30460731	KDRI factors + WIT >30 min	C-statistic	Overall	apparent	0.731	Reported interval 0.66–0.803	Selected
Vinson 2019 PMID 30656212	Model 3 (independent D-R factors plus D-R pairing variables), 30% validation set	C-statistic	Overall	internal split	0.629	95% CI 0.623–0.635	Selected
Bae 2020 PMID 32996170	Cox regression (DCGF)	C-statistic	Overall	geographic	0.637	95% CI 0.629–0.646	Component
Bae 2020 PMID 32996170	Cox regression (ACGF)	C-statistic	Overall	geographic	0.634	95% CI 0.628–0.64	Component
Bae 2020 PMID 32996170	Cox regression (DCGF), Uno C	C-statistic	Overall	geographic	0.623	Not reported	Component
Bae 2020 PMID 32996170	Cox regression (ACGF), Uno C	C-statistic	Overall	geographic	0.635	Not reported	Component
Chu 2020 PMID 33319199	4-variable KFRE, 5-year risk, overall cohort	C-statistic	5	external	0.81	95% CI 0.78–0.84	Selected

Report and PMID	Model and cohort	Metric	Horizon years	Evaluation	Estimate	Reported uncertainty	Relation to Table S4
Mottola 2020<br>PMID 33125000	3-month eGFR (linear), multicentre validation cohort	C-statistic	Overall	external	0.693	Reported range 0.664–0.721	Selected
Saha-Chaudhuri 2020<br>PMID 32637142	Post-transplant Cox model with DGF and time-varying longitudinal eGFR, all-cause graft loss	C-statistic	Overall	apparent	0.71	SE 0.047	Selected
Raynaud 2021<br>PMID 34756569	DISPO, European external validation cohort	Time-dependent AUC	Overall	external	0.845	95% CI 0.837–0.854	Component
Raynaud 2021<br>PMID 34756569	DISPO, US external validation cohort	Time-dependent AUC	Overall	external	0.82	95% CI 0.808–0.831	Component
Raynaud 2021<br>PMID 34756569	DISPO, South American external validation cohort	Time-dependent AUC	Overall	external	0.868	95% CI 0.856–0.88	Component
Raynaud 2021<br>PMID 34756569	DISPO, pooled randomised-trial external validation cohort	Time-dependent AUC	Overall	external	0.857	95% CI 0.84–0.875	Component
Roller 2022<br>PMID 36388342	GBRT CDSS, graft failure within 360 days (internal validation)	AUC	1.0	cross validation	0.953	95% CI 0.934–0.972	Selected
Scurt 2023<br>PMID 37901299	Navarro full score, 5-year graft survival	AUC	5	external	0.617	95% CI 0.513–0.722	Component
Scurt 2023<br>PMID 37901299	Navarro score >5 vs <=5, 5-year graft survival	AUC	5	external	0.567	95% CI 0.462–0.673	Component
Scurt 2023<br>PMID 37901299	Port score, 1-year graft survival	AUC	1	external	0.662	95% CI 0.369–0.955	Component
Scurt 2023<br>PMID 37901299	Port score, 3-year graft survival	AUC	3	external	0.603	95% CI 0.515–0.692	Component
Scurt 2023<br>PMID 37901299	Rao score (KDRI), 1-year graft survival	AUC	1	external	0.699	95% CI 0.459–0.939	Component
Scurt 2023<br>PMID 37901299	Rao score (KDRI), 3-year graft survival	AUC	3	external	0.557	95% CI 0.456–0.658	Component

Report and PMID	Model and cohort	Metric	Horizon years	Evaluation	Estimate	Reported uncertainty	Relation to Table S4
Scurt 2023<br>PMID 37901299	Rao score (KDRI), 5-year graft survival	AUC	5	external	0.576	95% CI 0.474–0.679	Component
Scurt 2023<br>PMID 37901299	Snoeijs score, 5-year graft survival	AUC	5	external	0.63	95% CI 0.513–0.746	Component
Scurt 2023<br>PMID 37901299	de Vusser score, 3-year graft survival	AUC	3	external	0.637	95% CI 0.538–0.736	Component
Scurt 2023<br>PMID 37901299	de Vusser score, 5-year graft survival	AUC	5	external	0.62	95% CI 0.524–0.717	Component
Scurt 2023<br>PMID 37901299	Remuzzi score, 3-year graft survival	AUC	3	external	0.605	95% CI 0.501–0.709	Component
Truchot 2023<br>PMID 36572246	RSF - North American cohort	C-statistic	7	external	0.853	95% CI 0.825–0.878	Component
Truchot 2023<br>PMID 36572246	RSF-ERT - North American cohort	C-statistic	7	external	0.852	95% CI 0.825–0.877	Component
Truchot 2023<br>PMID 36572246	CIF - North American cohort	C-statistic	7	external	0.857	95% CI 0.831–0.881	Component
Truchot 2023<br>PMID 36572246	XGBoost - North American cohort	C-statistic	7	external	0.855	95% CI 0.829–0.878	Component
Truchot 2023<br>PMID 36572246	RSF - European cohort	C-statistic	7	external	0.801	95% CI 0.768–0.831	Component
Truchot 2023<br>PMID 36572246	RSF-ERT - European cohort	C-statistic	7	external	0.814	95% CI 0.785–0.841	Component
Truchot 2023<br>PMID 36572246	CIF - European cohort	C-statistic	7	external	0.811	95% CI 0.782–0.838	Component
Truchot 2023<br>PMID 36572246	XGBoost - European cohort	C-statistic	7	external	0.81	95% CI 0.781–0.838	Component
Leite 2024<br>PMID 38743065	Perfusate LDH (Results/Abstract value)	AUC	0.25	apparent	0.84	95% CI 0.598–1.0	Selected

Report and PMID	Model and cohort	Metric	Horizon years	Evaluation	Estimate	Reported uncertainty	Relation to Table S4
Swolinsky 2024<br>PMID 37858309	Base risk factors + log2(eGFR) + log2(pNGAL) (Fine-Gray)	C-statistic	Overall	bootstrap	0.844	95% CI 0.791–0.897	Selected
Fan 2025<br>PMID 41254094	LightGBM, baseline model (year 1)	AUC	1	cross validation	0.684	95% CI 0.644–0.725	Component
Fan 2025<br>PMID 41254094	LightGBM, follow-up model (year 2+)	AUC	1	cross validation	0.896	95% CI 0.875–0.914	Component
Gosset 2025<br>PMID 40686676	Fine-Gray model, validation, year 1	C-statistic	1	geographic	0.66	95% CI 0.63–0.7	Horizon-specific
Labriffe 2025<br>PMID 41126939	AdGFS, 5-year horizon	Time-dependent AUC	5	external	0.76	95% CI 0.7–0.82	Selected
Labriffe 2025<br>PMID 41126939	AdGFS, 10-year horizon	Time-dependent AUC	10	external	0.73	95% CI 0.69–0.77	Horizon-specific
Yin 2025<br>PMID 40521169	Model 3 Cox (abbreviated iBOX + HLA matches + dCRC), testing cohort	AUC	10	internal split	0.868	95% CI 0.816–0.92	Selected
Banks 2026<br>PMID 41541788	DCGS linear predictor (ACY1, CysC, midkine, recipient age), discovery, all transplants	C-statistic	Overall	bootstrap	0.55	95% CI 0.48–0.63	Component
Banks 2026<br>PMID 41541788	DCGS linear predictor (ACY1, CysC, midkine), discovery, deceased donor only	C-statistic	Overall	bootstrap	0.52	95% CI 0.43–0.6	Component
Filho 2026<br>PMID 41580479	GBM 11-gene transcriptomic risk score, 30% test set	C-statistic	Overall	internal split	0.87	95% CI 0.77–0.95	Selected
Gao 2026<br>PMID 42272126	KAPTOR-full (XGBoost, 1-year landmark)	AUC	5	internal split	0.904	95% CI 0.82–0.973	Component
Gao 2026<br>PMID 42272126	KAPTOR-pre (XGBoost, pre-transplant)	AUC	5	internal split	0.813	95% CI 0.698–0.912	Component
Potluri 2026<br>PMID 41135690	Cox model F (Kidney Allograft Survival Index, final)	Time-dependent AUC	3	internal split	0.678	95% CI 0.677–0.679	Horizon-specific

Report and PMID	Model and cohort	Metric	Horizon years	Evaluation	Estimate	Reported uncertainty	Relation to Table S4
Shehab 2026<br>PMID 41615413	RSF combined clinical + proteomic	C-statistic	Overall	internal split	0.878	95% CI 0.714–0.964	Selected
Shen 2026<br>PMID 42270478	LGBM final model, internal validation cohort	AUC	1	internal split	0.714	95% CI 0.698–0.725	Selected

Table S5. Comparisons of machine learning and regression in the same cohort

Report	PMID	Outcome	Recipients	Metric	Evaluation	Same predictors	Regression	ML, mean of families	ML, best family	Difference (mean)	Difference (best)	ML families	Difference tested in the report	Summarized
Feng 2026	42001037	DGF	140	AUC	Temporal or geographic	Yes	0.839	0.810	0.839	−0.029	0.000	Bayesian network, stacked ensemble, k-nearest neighbors, neural network, penalized regression, single tree, support-vector machine, tree ensemble	Yes	Yes
Brier 2003	14605292	DGF	304	AUC	Internal	Yes	0.636	0.668	0.668	0.032	0.032	neural network	Yes	Yes
Costa 2020	32027717	DGF	443	AUC	Internal	Yes	0.695	0.820	0.886	0.125	0.191	neural network, support-vector machine, tree ensemble	No	Yes
Decruyenaere 2015b	26466993	DGF	497	AUC	Internal	Yes	0.817	0.729	0.825	−0.088	0.008	other, single tree, support-vector machine, tree ensemble	Yes	Yes
Salgado 2026	41884255	DGF	523	AUC	Internal	Yes	0.670	0.522	0.587	−0.148	−0.083	neural network, support-vector machine, tree ensemble	Yes	Yes
Jen 2021	33560727	DGF	2,089	AUC	Internal	No	0.748	0.760	0.760	0.011	0.011	neural network	No	Yes
Kawakita 2020	33110142	DGF	55,044	AUC	Internal	Yes	0.728	0.735	0.739	0.007	0.011	neural network, penalized regression, tree ensemble	No	Yes
Bae 2020	32996170	DGF	133,431	AUC	Temporal or geographic	Yes	0.721	0.720	0.720	−0.001	−0.001	tree ensemble	No	Yes
Milecki 2025	40413148	Graft	108	C-statistic	External	No	0.665	0.613	0.640	−0.052	−0.025	neural network,	No	Yes

Report	PMID	Outcome	Recipients	Metric	Evaluation	Same predictors	Regression	ML, mean of families	ML, best family	Difference (mean)	Difference (best)	ML families	Difference tested in the report	Summarized
		survival										penalized regression		
Mulugeta 2025	39901148	Graft survival	278	C-statistic	Internal	Yes	0.881	0.910	0.910	0.028	0.028	tree ensemble	No	Yes
Shen 2026	42270478	Graft survival	356	AUC	Not reported	Yes	0.723	0.616	0.796	−0.107	0.074	Bayesian network, neural network, support-vector machine, tree ensemble	No	Yes
Hamidi 2016	27057518	Graft survival	378	C-statistic	Internal	Unclear	0.766	0.965	0.965	0.199	0.199	tree ensemble	No	Yes
Achilonu 2024	39290362	Graft survival	1,738	AUC	Internal	Yes	0.670	0.815	0.925	0.145	0.255	neural network, support-vector machine, tree ensemble	Yes	Yes
Garcia-Lopez 2026	42088642	Graft survival	2,030	C-statistic	Internal	Yes	0.720	0.870	0.870	0.150	0.150	tree ensemble	No	Yes
Yin 2025	40521169	Graft survival	2,575	AUC	Internal	Yes	0.868	0.849	0.849	−0.020	−0.020	tree ensemble	No	Yes
Yoo 2017	28827646	Graft survival	3,117	C-statistic	Internal	Yes	0.650	0.745	0.745	0.095	0.095	single tree	No	Yes
Tang 2011	21701272	Graft survival	3,313	AUC	Internal	Yes	0.740	0.705	0.710	−0.035	−0.030	neural network, single tree	Yes	Yes
Fan 2025	41254094	Graft survival	4,728	AUC	Internal	Yes	0.728	0.753	0.790	0.026	0.063	neural network, support-vector machine, tree ensemble	No	Yes
Senanayake 2021	34154541	Graft survival	7,365	C-statistic	Internal	Yes	0.670	0.613	0.660	−0.057	−0.010	single tree, support-vector machine, tree ensemble	No	Yes
Truchot 2023	36572246	Graft survival	8,422	C-statistic	External	Published model	0.826	0.832	0.832	0.006	0.006	tree ensemble	No	Yes
Ali 2024c	39437817	Graft survival	29,713	C-statistic	Not reported	Published model	0.570	0.640	0.640	0.070	0.070	tree ensemble	Yes	Yes
Salaün 2024	39075081	Graft survival	36,653	C-statistic	Internal	Yes	0.618	0.628	0.629	0.010	0.011	neural network, tree ensemble	No	Yes
Naqvi 2021	34448704	Graft survival	52,827	AUC	Internal	Yes	0.620	0.653	0.670	0.033	0.050	neural network, support-vector machine, tree ensemble	No	Yes
Potluri 2026	41135690	Graft survival	75,867	Time-dependent AUC	Internal	Yes	0.659	0.665	0.680	0.006	0.021	penalized regression, tree ensemble	No	Yes
Bae 2020	32996170	Graft survival	133,431	C-statistic	Temporal or geographic	Yes	0.632	0.631	0.631	−0.001	−0.001	tree ensemble	No	Yes

Report	PMID	Outcome	Recipients	Metric	Evaluation	Same predictors	Regression	ML, mean of families	ML, best family	Difference (mean)	Difference (best)	ML families	Difference tested in the report	Summarized
Ali 2024a	38552178	Graft survival	156,749	Time-dependent AUC	External	Published model	0.590	0.620	0.620	0.030	0.030	tree ensemble	No	Yes
Paquette 2022	35700006	Graft survival	180,141	C-statistic	Internal	Yes	0.646	0.650	0.657	0.004	0.011	neural network, tree ensemble	No	Yes
Apanisile 2026	42361104	Graft survival	228,855	C-statistic	Internal	Yes	0.687	0.686	0.687	−0.001	0.000	penalized regression, support-vector machine, tree ensemble	No	Yes
Tirasattayapitak 2024	39768425	DGF			Regression: Apparent; ML: Internal		0.760	0.903						No (different evaluation designs)
Ali 2024b	38684469	Graft survival			Regression: Not reported; ML: Internal		0.560	0.672						No (published index; evaluation sample not stated)

ML models were averaged within family; the main comparison uses the mean of the family averages. Difference is ML minus regression, calculated before rounding. Published model: the regression comparator was a published model applied unchanged. Comparisons were not pooled.

Table S6. PROBAST signaling questions and judgments for each report

Report	PMID	Outcome	1.1	1.2	2.1	2.2	2.3	3.1	3.2	3.3	3.4	3.5	3.6	4.1	4.2	4.3	4.4	4.5	4.6	4.7	4.8	4.9	Participants	Predictors	Outcome domain	Analysis	Overall
Brier 2003	14605292	DGF	Y	N	PY	PY	Y	Y	Y	Y	Y	PY	Y	N	PY	PN	PN	Y	Y	N	PN	NA	High	Low	Low	High	High
Irish 2003	14569108	DGF	Y	N	PY	PY	Y	PY	Y	Y	PY	PY	Y	PY	Y	PN	PN	PY	Y	N	PY	PY	High	Low	Low	High	High
Avihingsanon 2005	15888558	DGF	Y	PY	Y	PY	Y	Y	PY	Y	Y	PY	Y	N	PY	PY	PN	N	Y	N	PN	NA	Low	Low	Low	High	High
Grossberg 2006	16436956	DGF	Y	N	Y	PY	Y	Y	Y	Y	Y	PY	Y	PN	PY	PN	PN	NA	Y	PY	NA	NA	High	Low	Low	High	High
Mishra 2006	16528543	DGF	Y	PN	Y	Y	Y	Y	Y	Y	Y	PY	Y	N	PN	NI	NI	NA	Y	NA	N	NA	High	Low	Low	High	High
Parikh 2006	16827865	DGF	Y	NI	Y	Y	Y	Y	Y	Y	Y	Y	Y	N	PN	PY	NI	NA	Y	NA	N	NA	Unclear	Low	Low	High	High
Moore 2007	17556406	DGF	Y	PN	Y	PY	Y	Y	Y	Y	Y	PY	Y	PN	Y	PN	NI	NA	Y	N	NA	NA	High	Low	Low	High	High
Mueller 2008	18021287	DGF	Y	PY	Y	Y	PY	Y	Y	Y	Y	PY	Y	N	PN	PY	NI	N	Y	N	N	PY	Low	Low	Low	High	High
Jeldres 2009	19829730	DGF	Y	N	Y	PY	Y	Y	Y	Y	Y	PY	Y	N	N	PN	PN	PY	Y	Y	PY	PY	High	Low	Low	High	High
Tiong 2009	19167732	Graft survival	Y	NI	Y	PY	Y	PY	NI	PY	PY	PY	PY	Y	Y	Y	PY	Y	Y	Y	Y	PY	Unclear	Low	Unclear	Low	Unclear
Alachkar 2010	21094786	DGF	Y	PY	Y	PY	Y	PY	PY	Y	Y	PY	Y	N	N	PN	NI	NA	Y	NA	N	NA	Low	Low	Low	High	High
Dolegowska 2010	20530808	DGF	PY	NI	Y	Y	Y	PY	PY	Y	Y	PY	Y	PN	N	PY	NI	NA	Y	NA	N	NA	Unclear	Low	Low	High	High
Dołęgowska 2010	20829075	DGF	Y	NI	Y	PY	Y	Y	PY	Y	Y	PY	Y	PN	N	PY	NI	NA	Y	NA	N	NA	Unclear	Low	Low	High	High
Greco 2010	20534243	Graft survival	Y	PY	PY	PY	PY	PY	NI	PY	PY	PY	PN	PN	N	PY	NI	PY	PN	N	PY	NA	Low	Low	High	High	High
Hall 2010	19762491	DGF	Y	PY	Y	Y	Y	Y	Y	PY	Y	PY	Y	N	N	PY	NI	NA	Y	NA	N	NA	Low	Low	Low	High	High
Irish 2010	20883559	DGF	Y	PN	Y	PY	Y	PY	Y	Y	PY	PY	Y	Y	Y	PY	PN	Y	Y	Y	PY	Y	High	Low	Low	High	High
Kasiske 2010	20801565	Graft survival	Y	Y	Y	PY	Y	PY	Y	Y	Y	PY	Y	PY	PY	Y	PN	N	Y	PY	Y	PY	Low	Low	Low	High	High
Kusaka 2010	20525436	DGF	Y	NI	Y	Y	Y	PY	PN	Y	Y	PY	PY	PN	N	Y	NI	NA	Y	NA	N	NA	Unclear	Low	High	High	High
Moers 2010	20861807	DGF	Y	PY	Y	Y	Y	Y	Y	Y	Y	PY	Y	NI	Y	PN	NI	NA	Y	NA	N	NA	Low	Low	Low	High	High
Plata-Munoz 2010	19719466	Both	Y	PY	Y	PY	Y	PY	Y	Y	Y	PY	Y	PN	PY	PY	NI	NA	Y	N	NA	NA	Low	Low	Low	High	High
Bataille 2011	21956199	DGF	Y	Y	Y	Y	Y	Y	Y	Y	Y	Y	Y	PN	N	Y	NI	NA	Y	NA	N	NA	Low	Low	Low	High	High
Hollmen 2011a	20861824	DGF	Y	PY	PY	PY	Y	PY	Y	Y	Y	PY	Y	Y	PN	PN	PN	NA	Y	NA	N	NA	Low	Low	Low	High	High

Report	PMID	Outcome	1.1	1.2	2.1	2.2	2.3	3.1	3.2	3.3	3.4	3.5	3.6	4.1	4.2	4.3	4.4	4.5	4.6	4.7	4.8	4.9	Participants	Predictors	Outcome domain	Analysis	Overall
Hollmen 2011b	21545740	DGF	Y	PY	Y	Y	Y	PY	Y	Y	Y	PY	Y	Y	PN	PN	NI	NA	Y	NA	N	NA	Low	Low	Low	High	High
Jochmans 2011	21834917	DGF	Y	PN	Y	Y	Y	Y	Y	Y	Y	PY	Y	PY	Y	PN	NI	NA	Y	NA	N	NA	High	Low	Low	High	High
Korbély 2011	20819195	DGF	PN	NI	Y	PY	PY	Y	Y	Y	Y	PY	Y	N	Y	PY	NI	N	Y	PY	PN	Y	High	Low	Low	High	High
Tang 2011	21701272	Graft survival	Y	PN	Y	PY	PN	PY	NI	Y	Y	PY	Y	PY	NI	PN	PN	PY	PN	N	PY	PY	High	High	Unclear	High	High
Brown 2012 †	23221105	Graft survival	Y	PY	PY	PY	Y	PY	NI	Y	NI	PY	PY	NI	NI	NI	NI	NI	NI	NI	PY	NA	Low	Low	Unclear	Unclear	Unclear
Dołęgowska 2012	22279544	DGF	Y	PY	Y	Y	Y	PY	PY	Y	Y	Y	Y	PN	N	PY	NI	NA	Y	NA	N	NA	Low	Low	Low	High	High
Rodrigo 2012	22026730	DGF	Y	PY	Y	PY	Y	Y	Y	Y	Y	PY	Y	Y	PY	PN	PN	NA	Y	Y	NA	NA	Low	Low	Low	High	High
Fonseca 2013	24288591	DGF	Y	PN	Y	Y	Y	Y	Y	Y	Y	PY	PY	PY	PN	PN	PN	NA	Y	NA	N	NA	High	Low	Low	High	High
Gourishankar 2013	23731367	Both	Y	Y	PY	PY	Y	PY	NI	PY	PY	PY	Y	Y	PY	NI	NI	NA	Y	N	NA	NA	Low	Low	Unclear	High	High
Ho 2013	23192155	Graft survival	Y	N	Y	PY	Y	Y	NI	Y	Y	PY	Y	N	PY	PN	PN	N	PY	N	N	NI	High	Low	Unclear	High	High
Kreepala 2013	23282320	DGF	Y	PY	Y	PY	PY	Y	Y	Y	Y	PY	Y	N	PY	PY	NI	N	Y	N	PY	PY	Low	Low	Low	High	High
Schmidt 2013	23291472	DGF	Y	PY	Y	PY	Y	Y	Y	Y	Y	PY	Y	PN	PN	PY	NI	NA	Y	NA	N	NA	Low	Low	Low	High	High
Welberry Smith 2013	23739232	DGF	Y	NI	Y	PY	Y	Y	Y	Y	Y	PY	Y	PN	PN	PN	NI	NA	Y	NA	N	NA	Unclear	Low	Low	High	High
Buemi 2014	25011070	DGF	Y	Y	Y	PY	PY	Y	Y	Y	Y	PY	PN	N	Y	Y	NI	NA	Y	NA	N	NA	Low	Low	High	High	High
Chapal 2014	24897036	DGF	Y	PN	Y	PY	Y	Y	Y	Y	Y	PY	Y	PY	PN	PN	PN	N	Y	Y	PY	Y	High	Low	Low	High	High
Fonseca 2014	24406454	DGF	Y	PN	Y	PY	Y	Y	Y	Y	Y	PY	Y	PN	PN	PN	NI	NA	Y	NA	N	NA	High	Low	Low	High	High
Goncalves-Primo 2014	24503763	DGF	Y	PY	Y	Y	PY	Y	Y	Y	Y	PY	Y	N	N	PY	NI	NA	Y	NA	N	NA	Low	Low	Low	High	High
Pajek 2014	25027586	DGF	Y	PY	Y	Y	Y	Y	PY	PN	Y	PY	Y	PN	PN	PN	NI	NA	Y	NA	N	NA	Low	Low	High	High	High
Wohlfahrtova 2014	24092381	DGF	Y	PN	Y	Y	Y	Y	Y	Y	Y	PY	Y	N	PY	PN	NI	N	Y	N	PN	NI	High	Low	Low	High	High
Burgos Revilla 2015	25645760	DGF	Y	PN	Y	Y	Y	PY	PN	Y	Y	PY	Y	N	PN	PN	PN	NA	Y	NA	N	NA	High	Low	High	High	High
Cantaluppi 2015	26125566	DGF	Y	PY	Y	PY	Y	Y	Y	Y	Y	PY	PY	PN	PN	Y	NI	NA	Y	NA	N	NA	Low	Low	Low	High	High
Chaumont 2015	26448870	DGF	Y	PY	PY	PY	PY	PY	PY	Y	PY	PY	Y	PY	N	PN	PN	Y	Y	PY	N	Y	Low	Low	Low	High	High
Decruyenaere 2015a	26387917	DGF	Y	PN	PY	PY	Y	Y	Y	Y	Y	PY	Y	N	Y	PN	NI	Y	Y	Y	N	Y	High	Low	Low	High	High
Decruyenaere 2015b	26466993	DGF	Y	N	PY	PY	NI	Y	Y	Y	Y	PY	Y	N	Y	PY	PN	PY	Y	N	PN	NI	High	Unclear	Low	High	High
Fonseca 2015a	25458832	DGF	Y	Y	Y	PY	PY	Y	Y	Y	Y	PY	PY	PN	PY	PY	NI	NA	Y	NA	N	NA	Low	Low	Low	High	High
Fonseca 2015b	26162495	DGF	Y	PN	Y	PY	Y	Y	Y	Y	Y	PY	Y	N	Y	PN	NI	PN	Y	N	N	PN	High	Low	Low	High	High
Gómez 2015	25645764	DGF	Y	PN	Y	PY	Y	PY	PN	Y	Y	PY	Y	PY	PN	PN	PN	NA	Y	NA	N	NA	High	Low	High	High	High
Hosgood 2015	26313559	DGF	Y	Y	Y	Y	Y	Y	Y	Y	Y	PY	Y	N	N	Y	NI	Y	Y	N	N	PN	Low	Low	Low	High	High
Muller 2015	25611655	DGF	Y	PY	Y	Y	Y	Y	Y	Y	Y	PY	Y	PY	Y	PN	PN	NA	Y	NA	N	NA	Low	Low	Low	High	High
Pianta 2015a	25083615	DGF	Y	N	Y	PY	Y	PY	Y	Y	Y	PY	PY	N	Y	PN	NI	NA	Y	NA	N	NA	High	Low	Low	High	High
Pianta 2015b	25938452	DGF	Y	PY	Y	PY	Y	Y	Y	Y	Y	PY	PY	N	PY	PN	NI	NA	Y	NA	N	NA	Low	Low	Low	High	High
Pianta 2015c	26174580	DGF	Y	N	Y	PY	Y	PY	Y	PY	Y	PY	Y	N	Y	PN	NI	NA	Y	NA	N	NA	High	Low	Low	High	High
Yong 2015	26293021	DGF	Y	PY	PY	Y	PY	Y	Y	Y	Y	PY	Y	PN	Y	PN	NI	NA	Y	NA	NA	NA	Low	Low	Low	High	High
Zaza 2015	25164408	DGF	Y	PY	PY	PY	Y	Y	Y	Y	PY	PY	Y	Y	Y	PN	PN	N	Y	PN	N	NI	Low	Low	Low	High	High
Hamidi 2016	27057518	Graft survival	Y	N	PY	PY	NI	NI	NI	NI	PY	NI	PY	N	Y	PN	Y	Y	Y	N	PY	NA	High	Unclear	Unclear	High	High
Hassanain 2016	27569934	Graft survival	Y	N	Y	PY	Y	Y	Y	Y	Y	PY	Y	N	N	PN	PN	Y	Y	PY	N	NI	High	Low	Low	High	High
Koo 2016	26986138	DGF	Y	PY	Y	Y	Y	PY	PY	Y	Y	PY	Y	PN	Y	PY	NI	PY	Y	Y	PY	PY	Low	Low	Low	High	High
Mojtahedzadeh 2016	28855931	DGF	Y	PY	Y	Y	Y	PY	PY	N	Y	PY	PN	N	PY	PN	PN	NA	Y	NA	N	NA	Low	Low	High	High	High
Nguyen 2016	26425877	DGF	Y	PY	Y	Y	PY	PY	PY	Y	Y	PY	Y	PN	Y	Y	NI	N	Y	N	N	PY	Low	Low	Low	High	High
Ding 2017	29052563	DGF	Y	PN	PY	PY	Y	Y	Y	Y	Y	PY	Y	N	PN	PY	NI	N	Y	Y	PN	PN	High	Low	Low	High	High
Martínez-Mier 2017	28591501	Graft survival	Y	NI	PY	PY	Y	PY	NI	Y	PY	PY	Y	PN	Y	PN	NI	NA	PN	PY	NA	NA	Unclear	Low	Unclear	High	High
Michalak 2017	29104860	DGF	Y	N	PY	PY	Y	Y	Y	Y	Y	PY	Y	PN	PY	PN	PN	NA	Y	Y	NA	NA	High	Low	Low	High	High
Molnar 2017	28270930	Graft survival	Y	Y	Y	PY	PY	Y	Y	Y	Y	PY	Y	N	PY	PY	PN	PY	Y	Y	PY	Y	Low	Low	Low	High	High
Prémaud 2017	28671951	Graft survival	Y	PY	Y	PY	Y	Y	Y	Y	Y	PY	Y	PN	N	PN	PY	Y	PY	Y	PN	NI	Low	Low	Low	High	High
Yoo 2017	28827646	Graft survival	Y	PN	PY	PY	PY	PY	NI	PY	PY	PY	PN	N	N	PN	PN	PY	PY	N	PN	NA	High	Low	High	High	High
van Balkom 2017	28476951	DGF	Y	PY	PN	Y	Y	PY	Y	Y	Y	PY	Y	N	Y	PY	NI	N	Y	N	N	NI	Low	High	Low	High	High
Ding 2018	30425191	DGF	Y	PY	Y	PY	Y	Y	Y	Y	Y	PY	Y	N	N	PY	NI	N	Y	PY	PN	PN	Low	Low	Low	High	High
Kers 2018	29462353	DGF	Y	N	PY	PY	Y	PY	Y	Y	Y	PY	Y	Y	NI	PY	Y	NA	Y	Y	NA	NA	High	Low	Low	Unclear	High
Le Borgne 2018	28633603	Both	Y	PY	Y	PY	Y	Y	Y	Y	Y	PY	Y	Y	Y	PY	NI	NA	Y	NA	N	NA	Low	Low	Low	High	High
Maier 2018	29304176	DGF	Y	Y	Y	PY	Y	Y	Y	Y	Y	PY	PY	PY	Y	PN	NI	NA	Y	NA	N	NA	Low	Low	Low	High	High
McMahon 2018	29034718	DGF	Y	Y	Y	PY	Y	Y	Y	Y	Y	PY	Y	PY	N	Y	NI	NA	Y	NA	N	NA	Low	Low	Low	High	High
Peters-Sengers 2018a	28731905	Graft survival	Y	Y	PY	PY	Y	PY	Y	Y	Y	PY	Y	PY	PY	PY	PY	NA	Y	Y	NA	NA	Low	Low	Low	Low	Low

Report	PMID	Outcome	1.1	1.2	2.1	2.2	2.3	3.1	3.2	3.3	3.4	3.5	3.6	4.1	4.2	4.3	4.4	4.5	4.6	4.7	4.8	4.9	Participants	Predictors	Outcome domain	Analysis	Overall
Schmitt 2018	29505681	DGF	Y	PY	Y	PY	Y	Y	PY	Y	Y	PY	Y	N	PY	PY	NI	NA	Y	NA	N	NA	Low	Low	Low	High	High
Sun 2018	29871001	DGF	Y	PY	PY	PY	PY	PY	PY	PY	Y	PY	Y	PN	PY	PN	PN	NI	Y	N	PN	PY	Low	Low	Low	High	High
Tai 2018	29407336	DGF	Y	PY	Y	PY	Y	PY	Y	Y	Y	PY	Y	N	PY	NI	NI	N	Y	N	N	PY	Low	Low	Low	High	High
Zhang 2018	29416727	DGF	Y	PY	PY	PY	Y	Y	Y	Y	Y	PY	Y	Y	PY	PN	PN	NA	Y	Y	NA	NA	Low	Low	Low	High	High
Aceto 2019	30806201	DGF	Y	PY	Y	PY	Y	Y	Y	Y	Y	PY	Y	PN	N	PN	PN	NA	Y	NA	N	NA	Low	Low	Low	High	High
Han 2019	30747854	DGF	Y	N	Y	PY	Y	PY	PY	Y	Y	PY	Y	N	Y	PN	NI	PY	Y	N	N	PN	High	Low	Low	High	High
Hu 2019	31722677	Graft survival	Y	PY	Y	PY	Y	PY	Y	PY	Y	PY	Y	N	PY	PY	NI	Y	PN	N	N	NI	Low	Low	Low	High	High
Katou 2019	31703290	DGF	Y	PN	Y	PY	Y	PY	PY	Y	Y	PY	PY	N	N	PN	NI	NA	Y	NA	N	NA	High	Low	Low	High	High
Khalid 2019	30837502	DGF	PN	PY	PN	PY	Y	Y	Y	Y	Y	PY	PY	N	PY	PN	NI	N	Y	N	N	NI	High	High	Low	High	High
Kim 2019	31847814	DGF	Y	PN	Y	PY	PY	Y	Y	PN	Y	PY	PN	N	Y	PN	NI	NA	Y	NA	N	NA	High	Low	High	High	High
Kusaka 2019	30460731	Graft survival	Y	N	Y	PY	Y	PY	NI	Y	PY	PY	PY	PY	PY	PN	PN	N	PN	N	N	NI	High	Low	Unclear	High	High
Roest 2019	30444806	DGF	PN	N	Y	PY	Y	PY	Y	Y	Y	PY	Y	N	PY	PN	NI	NA	Y	NA	PN	NA	High	Low	Low	High	High
Stamenic 2019	31093367	Graft survival	Y	PN	Y	PY	Y	Y	Y	Y	Y	PY	Y	PN	PN	PN	PN	N	Y	PY	N	PY	High	Low	Low	High	High
Truche 2019	31732205	DGF	Y	N	Y	PY	Y	Y	Y	Y	Y	PY	Y	N	Y	PN	PN	NA	Y	NA	N	NA	High	Low	Low	High	High
Vinson 2019	30656212	Graft survival	Y	Y	Y	PY	Y	Y	Y	Y	Y	PY	PY	PY	PY	PN	PN	N	Y	PY	PY	PY	Low	Low	Low	High	High
Weissenbacher 2019	31567358	DGF	Y	PY	Y	Y	Y	Y	Y	Y	Y	PY	Y	PN	PY	PN	PN	NA	Y	NA	N	NA	Low	Low	Low	High	High
Bae 2020	32996170	Both	Y	Y	Y	PY	Y	PY	Y	Y	Y	PY	Y	Y	Y	Y	Y	Y	Y	Y	PY	NI	Low	Low	Low	Unclear	Unclear
Chen 2020	33276737	DGF	Y	PY	PY	PY	PY	Y	Y	Y	Y	PY	Y	N	PY	PN	PN	N	Y	Y	PN	PN	Low	Low	Low	High	High
Chu 2020	33319199	Graft survival	Y	PY	Y	Y	Y	Y	Y	Y	Y	PY	Y	Y	Y	PN	PN	NA	Y	Y	NA	NA	Low	Low	Low	High	High
Costa 2020	32027717	DGF	Y	PN	Y	PY	NI	Y	Y	Y	Y	PY	Y	N	PY	PN	PN	N	Y	N	PN	NA	High	Unclear	Low	High	High
Halpern 2020	33144442	DGF	Y	PY	PY	PY	Y	Y	Y	Y	Y	PY	Y	PY	N	PN	NI	PY	Y	N	PY	PY	Low	Low	Low	High	High
Kawakita 2020	33110142	DGF	Y	Y	Y	PY	Y	Y	Y	Y	Y	PY	Y	Y	Y	PN	PY	Y	Y	Y	PY	NA	Low	Low	Low	High	High
Mottola 2020	33125000	Graft survival	Y	PN	Y	PY	Y	Y	Y	Y	Y	PY	PY	PY	PN	PN	NI	NA	PY	NA	PN	NA	High	Low	Low	High	High
Okada 2020	31811409	DGF	Y	PN	Y	PY	Y	PY	PN	Y	Y	PY	PY	N	N	PY	NI	NA	Y	NA	N	NA	High	Low	High	High	High
Saha-Chaudhuri 2020	32637142	Both	Y	PN	Y	PY	Y	Y	Y	Y	Y	PY	Y	N	Y	PN	PN	N	Y	N	N	PY	High	Low	Low	High	High
Zhang 2020	33376751	Graft survival	PY	NI	PY	PY	Y	PY	NI	Y	PY	PY	PY	N	PY	PY	NI	N	Y	N	PN	Y	Unclear	Low	Unclear	High	High
Zheng 2020	32338125	DGF	Y	PN	Y	PY	Y	Y	Y	Y	Y	PY	Y	N	PN	PN	PN	N	Y	N	N	PY	High	Low	Low	High	High
Arias-Cabrales 2021	33841865	DGF	Y	N	Y	PY	PN	Y	Y	Y	Y	PY	PN	PN	N	PN	PN	NA	Y	NA	N	NA	High	High	High	High	High
Ietto 2021	34728075	DGF	Y	N	PY	PY	Y	NI	NI	PY	NI	PY	PY	PN	PY	PN	PN	NA	Y	N	NA	NA	High	Low	Unclear	High	High
Irish 2021	34386656	Both	Y	PY	Y	PY	PY	Y	PY	PY	PN	PY	Y	Y	PY	PN	PY	PY	Y	Y	N	PY	Low	Low	High	High	High
Jen 2021	33560727	DGF	Y	N	Y	PY	Y	Y	Y	Y	Y	PY	Y	Y	PY	PN	PN	PY	Y	N	PN	NA	High	Low	Low	High	High
Kaboré 2021	32989448	Graft survival	Y	Y	Y	PY	Y	Y	Y	Y	Y	PY	Y	PY	Y	PN	PN	Y	Y	PY	PY	Y	Low	Low	Low	High	High
Kemmner 2021	34208140	DGF	Y	Y	Y	Y	Y	Y	Y	Y	Y	PY	Y	N	PY	Y	NI	NA	Y	NA	N	NA	Low	Low	Low	High	High
Konieczny 2021	34830526	DGF	Y	NI	PY	PY	Y	Y	Y	Y	PY	PY	Y	PN	PY	PY	NI	PY	Y	N	PN	NA	Unclear	Low	Low	High	High
Naqvi 2021	34448704	Graft survival	Y	N	Y	PY	Y	Y	Y	Y	Y	PY	PY	Y	PY	PN	PN	Y	PN	N	PY	NA	High	Low	Low	High	High
Pan 2021	34876844	DGF	Y	PN	PY	PY	Y	Y	Y	Y	Y	PY	Y	N	N	PN	PN	N	Y	PY	PN	PN	High	Low	Low	High	High
Qiao 2021	34924501	DGF	Y	PN	Y	PY	Y	PY	PY	Y	Y	PY	Y	N	PY	PY	NI	N	Y	N	N	PY	High	Low	Low	High	High
Raynaud 2021	34756569	Graft survival	Y	PY	PY	PY	Y	Y	Y	Y	Y	PY	Y	PY	NI	NI	NI	PY	PN	Y	Y	NI	Low	Low	Low	High	High
Senanayake 2021	34154541	Graft survival	Y	PY	Y	PY	Y	Y	NI	Y	Y	PY	Y	N	PN	Y	PY	PY	PY	Y	PY	PY	Low	Low	Unclear	High	High
Xue 2021	33719820	DGF	Y	PY	Y	PY	Y	Y	Y	Y	Y	PY	Y	Y	PY	PN	PN	N	Y	Y	N	PY	Low	Low	Low	High	High
Yu 2021	33968652	DGF	Y	NI	Y	PY	Y	Y	Y	Y	Y	PY	Y	N	Y	Y	NI	N	Y	PY	N	Y	Unclear	Low	Low	High	High
Divard 2022	36418380	Graft survival	Y	Y	Y	PY	Y	Y	Y	Y	Y	PY	Y	PN	PY	PY	NI	NA	PY	Y	NA	NA	Low	Low	Low	High	High
Dou 2022	35281025	Graft survival	PY	NI	Y	PY	PY	PY	NI	Y	PY	PY	NI	NI	PY	NI	NI	N	Y	N	N	NI	Unclear	Low	Unclear	High	High
Goujon 2022	35945114	DGF	Y	PY	PN	PY	Y	Y	Y	Y	Y	PY	Y	N	Y	PY	NI	PY	Y	N	N	NI	Low	High	Low	High	High
Jeong 2022	35610279	Graft survival	Y	PY	Y	PY	Y	Y	NI	Y	Y	PY	Y	N	PY	PY	NI	Y	PN	N	N	NI	Low	Low	Unclear	High	High
Kurian 2022	36406898	Both	Y	PN	Y	PY	PN	PN	PN	PY	Y	PY	PY	Y	Y	PY	Y	Y	Y	Y	Y	PY	High	High	High	Low	High
Li 2022	36035383	DGF	Y	PN	Y	PY	Y	PY	PY	PY	Y	PY	Y	N	PN	PN	PN	Y	Y	Y	PY	Y	High	Low	Low	High	High
Lu 2022	36393969	Graft survival	PY	NI	PY	PY	NI	PY	NI	PY	NI	PY	NI	NI	PN	NI	NI	NA	PN	NA	N	NA	Unclear	Unclear	Unclear	High	High
Matas 2022	33675321	DGF	Y	PN	PY	PY	Y	Y	Y	Y	Y	PY	Y	Y	PY	PN	PN	NA	Y	PN	NA	NA	High	Low	Low	High	High
Miret Alomar 2022	33887895	DGF	Y	PY	PY	Y	Y	Y	Y	Y	Y	PY	Y	PN	PY	PY	NI	PN	Y	N	N	PY	Low	Low	Low	High	High
Pang 2022	36483557	Graft survival	Y	PN	Y	PY	Y	PY	NI	Y	PY	PY	PY	N	PY	PN	NI	N	Y	N	PN	PY	High	Low	Unclear	High	High
Paquette 2022	35700006	Graft survival	Y	PY	Y	PY	Y	Y	PY	Y	Y	PY	Y	PY	NI	PN	NI	Y	Y	Y	PY	NA	Low	Low	Low	High	High

Report	PMID	Outcome	1.1	1.2	2.1	2.2	2.3	3.1	3.2	3.3	3.4	3.5	3.6	4.1	4.2	4.3	4.4	4.5	4.6	4.7	4.8	4.9	Participants	Predictors	Outcome domain	Analysis	Overall
Roller 2022	36388342	Graft survival	Y	PN	PY	PY	Y	Y	Y	Y	Y	PY	Y	NI	PY	PN	NI	PY	PN	N	Y	NA	High	Low	Low	High	High
Scurt 2022	36043436	DGF	Y	PN	Y	PY	PY	Y	Y	Y	Y	PY	Y	PY	PY	PN	NI	NA	Y	NA	N	NA	High	Low	Low	High	High
Tepel 2022	35260630	DGF	Y	Y	Y	Y	Y	Y	Y	Y	Y	Y	Y	Y	N	PY	NI	NA	Y	NA	PN	NA	Low	Low	Low	High	High
Wei 2022	35211472	DGF	Y	NI	PY	PY	PY	NI	NI	PY	NI	PY	PY	PN	PY	PY	NI	PY	Y	N	PN	Y	Unclear	Low	Unclear	High	High
Wu 2022	36532016	Both	PY	NI	PY	PY	PY	NI	NI	NI	NI	NI	PY	PN	PY	PY	NI	PY	Y	N	PN	Y	Unclear	Low	Unclear	High	High
Xiao 2022	36372566	DGF	Y	PY	Y	PY	Y	Y	Y	Y	Y	PY	Y	N	Y	PN	PN	N	Y	Y	PN	Y	Low	Low	Low	High	High
Yi 2022	34757124	Graft survival	Y	PY	PY	PY	Y	PY	PY	Y	Y	PY	Y	PN	PN	PN	PN	PY	PY	N	N	NI	Low	Low	Low	High	High
Zhang 2022	36346017	DGF	Y	NI	Y	PY	Y	PY	PY	Y	Y	PY	Y	PN	PY	PY	NI	NA	Y	NA	N	NA	Unclear	Low	Low	High	High
Zhao 2022	35971094	DGF	Y	PN	Y	PY	Y	Y	Y	Y	Y	PY	Y	N	Y	PY	NI	N	Y	Y	PN	Y	High	Low	Low	High	High
Badrouchi 2023	38042904	Graft survival	Y	N	PY	PY	PY	PY	Y	Y	Y	PY	PY	N	NI	PN	PN	PY	PY	N	PN	NA	High	Low	Low	High	High
Bi 2023	37275548	Both	Y	NI	Y	PY	Y	PY	NI	Y	NI	PY	PY	N	Y	PN	NI	N	Y	N	PN	PY	Unclear	Low	Unclear	High	High
Dasariraju 2023	37120046	Graft survival	Y	NI	PY	PY	Y	PY	NI	Y	PY	PY	PY	NI	PY	NI	NI	PN	Y	N	Y	NA	Unclear	Low	Unclear	High	High
He 2023	37722724	DGF	Y	PY	Y	Y	Y	Y	Y	Y	Y	PY	Y	N	Y	PY	NI	PN	Y	N	N	PN	Low	Low	Low	High	High
Li 2023	37965311	DGF	PY	NI	PY	PY	PY	NI	NI	PY	NI	PY	PY	PY	Y	NI	NI	PY	Y	N	PN	PY	Unclear	Low	Unclear	High	High
Lin 2023a	37183066	DGF	Y	N	Y	PY	PY	PY	PY	Y	Y	PY	Y	N	PY	PN	PN	PY	Y	N	N	PN	High	Low	Low	High	High
Lin 2023b	37398694	DGF	Y	PY	Y	PY	PY	Y	Y	Y	Y	PY	Y	PY	PY	PN	NI	Y	Y	N	PY	NA	Low	Low	Low	High	High
Mao 2023	37739236	DGF	Y	PY	Y	Y	Y	PY	PY	Y	Y	PY	Y	N	PY	PY	NI	N	Y	N	N	PY	Low	Low	Low	High	High
Mulugeta 2023	37217892	Graft survival	Y	PN	PY	PY	PY	PY	NI	NI	PY	PY	NI	N	Y	Y	PY	Y	PN	Y	PN	NA	High	Low	Unclear	High	High
Pilichowska 2023	38137583	DGF	Y	PY	Y	PY	PY	Y	Y	Y	Y	PY	PY	PY	N	PN	NI	NA	Y	NA	N	NA	Low	Low	Low	High	High
Quinino 2023	36872507	DGF	Y	PN	Y	PY	Y	PY	PY	Y	Y	PY	Y	N	PY	PN	NI	Y	Y	N	PN	NA	High	Low	Low	High	High
Sandal 2023	37211638	Graft survival	Y	PN	Y	PY	Y	Y	Y	Y	Y	PY	Y	PY	PY	PN	Y	Y	PY	N	Y	PY	High	Low	Low	High	High
Scurt 2023	37901299	Both	Y	PN	PY	PY	N	PY	PY	Y	Y	PY	PY	PN	PY	PN	PN	NA	PN	N	NA	NA	High	High	Low	High	High
Song 2023	35603734	DGF	Y	PN	Y	PY	Y	PY	PY	PY	Y	PY	PY	N	PY	PN	PN	N	Y	N	N	NI	High	Low	Low	High	High
Truchot 2023	36572246	Graft survival	Y	PY	Y	PY	Y	Y	Y	Y	Y	PY	Y	PY	PY	Y	PN	Y	PY	Y	PY	NA	Low	Low	Low	High	High
Achilonu 2024	39290362	Graft survival	Y	PN	PY	PY	NI	Y	Y	Y	Y	PY	PN	N	PY	PN	PN	PN	PN	N	PN	NA	High	Unclear	High	High	High
Ali 2024a	38552178	Graft survival	Y	PY	Y	PY	Y	Y	NI	Y	Y	PY	Y	PY	PY	PN	PY	PY	PY	Y	PY	NA	Low	Low	Unclear	High	High
Ali 2024b	38684469	Graft survival	Y	PY	PY	PY	Y	PY	PY	Y	Y	PY	Y	PY	PY	PN	PY	Y	PY	Y	PY	NA	Low	Low	Low	High	High
Ali 2024c	39437817	Graft survival	Y	PN	PY	PY	Y	PY	Y	Y	Y	PY	Y	NI	Y	PN	PN	PY	Y	PY	PY	NA	High	Low	Low	High	High
Cirillo 2024	38460018	DGF	Y	NI	Y	PY	Y	PY	PY	Y	PY	PY	Y	N	Y	PN	NI	N	Y	N	PN	NI	Unclear	Low	Low	High	High
Denic 2024	38881746	Graft survival	Y	PN	Y	Y	Y	Y	PY	Y	Y	PY	Y	N	PY	PN	PN	N	Y	N	PY	Y	High	Low	Low	High	High
Ernst 2024	38632055	Both	Y	PN	Y	PY	Y	Y	PY	Y	PN	PY	Y	N	PN	PN	PN	N	PN	Y	N	PY	High	Low	High	High	High
Fabreti-Oliveira 2024	38797338	Graft survival	Y	N	PY	PY	PN	PY	PN	Y	PY	PY	PN	PN	PY	PY	NI	PY	Y	N	PN	NA	High	High	High	High	High
Ji 2024	39664334	Graft survival	Y	NI	PY	PY	PY	PY	NI	Y	PY	PY	PY	N	PY	PY	NI	N	Y	N	N	NI	Unclear	Low	Unclear	High	High
Leite 2024	38743065	Both	Y	N	Y	PY	Y	Y	Y	Y	Y	PY	Y	N	Y	PN	NI	NA	Y	NA	N	NA	High	Low	Low	High	High
Li 2024a	38641807	DGF	Y	PN	PY	PY	PY	Y	Y	Y	Y	PY	Y	N	PY	PN	NI	PN	Y	Y	PN	PN	High	Low	Low	High	High
Li 2024b	39188701	DGF	Y	NI	PY	PY	Y	PY	PN	Y	Y	PY	Y	PN	Y	Y	NI	NA	Y	NA	N	NA	Unclear	Low	High	High	High
Liu 2024	39304842	DGF	Y	N	Y	PY	PN	Y	Y	Y	Y	PY	Y	N	Y	PN	PN	N	Y	N	PN	NA	High	High	Low	High	High
Mao 2024a	39243908	Graft survival	PY	NI	PY	PY	PY	PY	NI	Y	PY	PY	PY	N	PY	NI	NI	N	PY	N	PN	NI	Unclear	Low	Unclear	High	High
Mao 2024b	39549926	Both	PY	NI	PY	PY	PY	PY	PY	Y	PY	PY	PY	PN	Y	NI	NI	PN	Y	PY	PN	PY	Unclear	Low	Low	High	High
Maslauskiene 2024	38611630	DGF	Y	Y	Y	Y	Y	Y	Y	Y	Y	PY	Y	N	PY	PN	NI	N	Y	N	N	PN	Low	Low	Low	High	High
Salaün 2024	39075081	Graft survival	Y	PY	Y	PY	Y	PY	PY	Y	Y	PY	Y	PY	Y	PY	PN	PY	PY	Y	PY	NA	Low	Low	Low	High	High
Scurt 2024	39047699	Graft survival	Y	PN	Y	PY	Y	PY	Y	Y	Y	PY	PY	PN	PY	PN	NI	NA	PY	N	NA	NA	High	Low	Low	High	High
Shen 2024	39602449	Graft survival	Y	NI	PY	PY	PY	PY	NI	Y	PY	PY	PY	NI	Y	NI	NI	N	Y	N	PN	PY	Unclear	Low	Unclear	High	High
Swolinsky 2024	37858309	Graft survival	Y	PY	Y	Y	Y	Y	Y	Y	Y	PY	Y	N	PY	PY	Y	NA	Y	NA	PY	NA	Low	Low	Low	High	High
Tam 2024	38993786	DGF	Y	PN	PY	Y	Y	PY	Y	Y	PY	PY	Y	N	Y	PN	PN	Y	Y	N	Y	NA	High	Low	Low	High	High
Tirasattayapitak 2024	39768425	DGF	Y	PN	PY	PY	N	Y	Y	Y	Y	PY	Y	PN	PN	PN	PY	PN	Y	PY	PY	NA	High	High	Low	High	High
Wang 2024	38163575	Both	PY	NI	Y	PY	NI	PY	NI	Y	PY	PY	PY	NI	PY	NI	NI	N	PY	PY	N	NI	Unclear	Unclear	Unclear	High	High
Yi 2024	37923131	Graft survival	Y	NI	Y	PY	Y	Y	PY	Y	Y	PY	Y	PN	PN	PY	NI	PY	PN	N	PN	NI	Unclear	Low	Low	High	High
Yu 2024	38184216	Graft survival	Y	N	Y	PY	Y	PY	Y	Y	Y	PY	PY	PN	PY	PN	PN	NA	PN	N	NA	NA	High	Low	Low	High	High
Zhang 2024	38495888	Graft survival	PY	NI	PY	PY	NI	PY	NI	Y	PY	PY	NI	NI	PN	NI	NI	NA	PY	NA	N	NA	Unclear	Unclear	Unclear	High	High
Ali 2025	39838510	Graft survival	Y	PN	Y	PY	Y	Y	NI	Y	Y	PY	Y	NI	Y	PN	PN	Y	PY	PY	PY	NA	High	Low	Unclear	High	High
Benning 2025	40475974	DGF	Y	PY	Y	PY	Y	PY	Y	Y	Y	PY	PY	Y	PN	PN	PN	NA	Y	NA	N	NA	Low	Low	Low	High	High

Report	PMID	Outcome	1.1	1.2	2.1	2.2	2.3	3.1	3.2	3.3	3.4	3.5	3.6	4.1	4.2	4.3	4.4	4.5	4.6	4.7	4.8	4.9	Participants	Predictors	Outcome domain	Analysis	Overall
Fan 2025	41254094	Graft survival	Y	Y	Y	PY	Y	PY	NI	PY	PY	PY	Y	PN	PY	PY	NI	Y	PN	N	Y	NA	Low	Low	Unclear	High	High
Gosset 2025	40686676	Graft survival	Y	PY	Y	PY	Y	Y	Y	Y	Y	PY	Y	Y	PY	PN	Y	PY	Y	Y	PY	PN	Low	Low	Low	High	High
He 2025	40236452	Graft survival	Y	N	Y	PY	PY	PY	Y	Y	Y	PY	Y	N	Y	PN	Y	PY	Y	Y	PY	PN	High	Low	Low	High	High
Hu 2025	40770395	Both	PY	NI	PY	PY	PY	PY	NI	PY	NI	PY	PY	PN	PY	PY	NI	N	Y	N	PN	NI	Unclear	Low	Unclear	High	High
Labriffe 2025	41126939	Graft survival	Y	PY	PY	PY	Y	Y	Y	Y	Y	PY	Y	Y	PN	PY	PN	NA	PY	N	NA	NA	Low	Low	Low	High	High
Ma 2025	40165952	DGF	Y	PN	Y	Y	Y	PY	PY	Y	Y	PY	Y	N	PY	PN	NI	N	Y	N	N	PN	High	Low	Low	High	High
Milecki 2025	40413148	Graft survival	Y	PN	PY	PY	Y	Y	Y	Y	Y	PY	Y	PN	PY	PN	NI	Y	Y	N	Y	NI	High	Low	Low	High	High
Mulugeta 2025	39901148	Graft survival	Y	PN	PY	PY	PN	PY	NI	PY	PY	PY	PN	N	Y	PY	Y	N	PN	PY	PN	NA	High	High	High	High	High
Qiu 2025	41080156	Graft survival	PY	NI	PY	PY	NI	PY	NI	Y	PY	PY	NI	NI	PY	NI	NI	N	PY	PY	PN	PY	Unclear	Unclear	Unclear	High	High
Salybekov 2025	41113995	Graft survival	Y	PN	PY	PY	Y	PY	NI	NI	PY	PY	NI	NI	PY	PN	Y	PY	PN	N	PN	NA	High	Low	Unclear	High	High
Shabani 2025	41068878	Graft survival	Y	PY	PY	PY	Y	PY	Y	Y	Y	PY	Y	N	PN	PN	PY	Y	PY	PY	PY	NA	Low	Low	Low	High	High
Shen 2025	41217598	Graft survival	Y	NI	PY	PY	NI	PY	NI	Y	PY	PY	NI	NI	PY	NI	NI	N	PY	N	PN	NA	Unclear	Unclear	Unclear	High	High
Suh 2025	39981772	Graft survival	PN	N	PY	PY	Y	PN	PN	Y	PY	PY	PN	Y	PY	PN	PN	N	PN	N	PY	NA	High	Low	High	High	High
Wrigge 2025	39891083	DGF	Y	PY	Y	PY	Y	Y	Y	Y	Y	PY	Y	N	PN	Y	NI	NA	Y	NA	N	NA	Low	Low	Low	High	High
Wu 2025a	40086342	Both	PY	NI	PY	PY	PY	NI	NI	PY	NI	PY	PY	NI	PY	NI	NI	PN	Y	PY	N	NA	Unclear	Low	Unclear	High	High
Wu 2025b	40624201	Graft survival	PY	NI	PY	PY	PY	PY	NI	Y	PY	PY	NI	PN	PY	PY	NI	PY	PY	N	PN	NA	Unclear	Low	Unclear	High	High
Yin 2025	40521169	Graft survival	Y	N	Y	PY	PY	PY	NI	Y	Y	PY	Y	PN	PY	PN	PY	Y	PY	Y	PY	NI	High	Low	Unclear	High	High
Yu 2025	41350284	DGF	Y	PN	Y	PY	Y	PY	PN	Y	Y	PY	Y	PY	PY	PN	PN	NA	Y	NA	PN	NA	High	Low	High	High	High
Apanisile 2026	42361104	Graft survival	Y	PN	Y	PY	PY	Y	Y	Y	Y	PY	PY	Y	Y	PN	PN	Y	PY	N	PY	PY	High	Low	Low	High	High
Banks 2026	41541788	Both	Y	NI	PY	Y	Y	PY	Y	PY	PY	PY	PY	N	Y	PN	Y	Y	Y	Y	PY	Y	Unclear	Low	Low	High	High
Dong 2026a	41736560	DGF	Y	N	Y	PY	Y	Y	Y	Y	Y	PY	Y	N	Y	PN	PN	PN	Y	Y	PY	Y	High	Low	Low	High	High
Dong 2026b	41947228	DGF	PY	NI	PY	PY	PY	PY	NI	Y	NI	PY	PY	NI	Y	NI	NI	N	Y	N	PN	NA	Unclear	Low	Unclear	High	High
Feng 2026	42001037	DGF	Y	PN	Y	PY	Y	Y	Y	Y	Y	PY	Y	N	Y	PN	NI	N	Y	Y	PN	Y	High	Low	Low	High	High
Filho 2026	41580479	Graft survival	Y	NI	Y	PY	Y	Y	Y	Y	Y	PY	PY	PN	Y	PY	NI	N	Y	Y	PN	NA	Unclear	Low	Low	High	High
Gao 2026	42272126	Graft survival	Y	N	Y	PY	Y	Y	Y	Y	Y	PY	Y	N	Y	PN	PN	PY	PN	Y	PN	NA	High	Low	Low	High	High
Garcia-Lopez 2026	42088642	Graft survival	Y	N	Y	PY	Y	Y	Y	Y	Y	PY	Y	N	PY	Y	Y	Y	Y	Y	PN	NA	High	Low	Low	High	High
He 2026	41969322	DGF	Y	PN	PY	PY	PY	PY	Y	PY	Y	PY	Y	N	NI	PN	PN	Y	Y	N	PN	NA	High	Low	Low	High	High
Ma 2026	42417841	DGF	Y	PN	Y	NI	PY	PY	PY	N	Y	PY	PN	PN	Y	PN	PN	NI	Y	N	PY	NI	High	Unclear	High	High	High
Nica 2026	41869992	DGF	Y	PN	PY	PY	PY	Y	Y	Y	PY	PY	Y	Y	Y	NI	NI	PY	Y	PY	PY	NA	High	Low	Low	Unclear	High
Patry 2026	41939206	DGF	Y	PN	PY	PY	Y	Y	Y	PY	Y	PY	PY	N	Y	PN	Y	PY	Y	Y	PN	Y	High	Low	Low	High	High
Potluri 2026	41135690	Both	Y	PY	Y	PY	PY	Y	Y	Y	Y	PY	Y	PY	PY	PN	PN	Y	Y	Y	PY	NI	Low	Low	Low	High	High
Ramalhete 2026	41977063	DGF	Y	PN	PY	PY	Y	Y	Y	Y	Y	PY	Y	N	PY	PN	PN	N	Y	Y	Y	NI	High	Low	Low	High	High
Ribeiro Neto 2026	42054170	DGF	Y	N	Y	Y	Y	PY	PY	Y	Y	PY	Y	N	N	PN	PN	NA	Y	NA	N	NA	High	Low	Low	High	High
Salgado 2026	41884255	DGF	Y	PY	PY	PY	Y	Y	Y	Y	Y	PY	Y	N	PY	PY	PN	N	Y	N	PN	NA	Low	Low	Low	High	High
Shehab 2026	41615413	Graft survival	PY	PN	Y	PY	Y	Y	Y	Y	Y	PY	PY	N	Y	PY	NI	PY	PN	PY	PN	NA	High	Low	Low	High	High
Shen 2026	42270478	Graft survival	Y	PN	PY	PY	NI	PY	NI	PY	PY	PY	Y	N	NI	PN	PN	N	PN	PY	PN	NA	High	Unclear	Unclear	High	High
Yan 2026	41782890	DGF	Y	Y	Y	PY	PN	PY	PY	PY	Y	PY	PN	N	PY	PY	NI	N	Y	N	N	NI	Low	High	High	High	High
Zhang 2026	42283098	Both	PY	NI	PY	PY	PY	PY	NI	PY	PY	PY	PY	N	Y	PN	NI	PY	Y	Y	PN	Y	Unclear	Low	Unclear	High	High
Zhu 2026	42216373	DGF	Y	PN	PY	PY	PY	Y	Y	Y	Y	PY	PY	N	Y	PN	PN	N	Y	N	N	Y	High	Low	Low	High	High

Y, yes; PY, probably yes; PN, probably no; N, no; NI, no information; NA, not applicable. A domain was at high risk when any question was answered N or PN, at low risk when all were Y or PY, and unclear otherwise. † Full text could not be obtained; data and risk-of-bias answers are from the published abstract.

Table S7. Reports excluded at full-text assessment, with reasons

Report	PMID	Reason for exclusion
Nicholson 1996	8819280	Outcome was not DGF or graft failure
Nyberg 2004	15233808	Review, secondary analysis or no original data
Lazzeri 2005	15880073	Association analysis without a prediction model or marker evaluation
Nyberg 2005	16249740	Association analysis without a prediction model or marker evaluation

Report	PMID	Reason for exclusion
Kaisar 2006	16509938	Review, secondary analysis or no original data
Kubal 2006	16939512	Review, secondary analysis or no original data
Irish 2007	17318090	Review, secondary analysis or no original data
Morrissey 2007	17414723	Review, secondary analysis or no original data
Kusaka 2008	18472448	Association analysis without a prediction model or marker evaluation
Ho 2008	18551064	Association analysis without a prediction model or marker evaluation
Mengel 2008	18782296	Review, secondary analysis or no original data
Moore 2009	19307797	Outcome was not DGF or graft failure
Schröppel 2010	20019169	Association analysis without a prediction model or marker evaluation
Glassock 2010	20498249	Review, secondary analysis or no original data
Moore 2010	20844467	Outcome was not DGF or graft failure
Goldfarb 2011	22088725	Review, secondary analysis or no original data
Hall 2012	22723447	Review, secondary analysis or no original data
Arnau 2012	23146452	Review, secondary analysis or no original data
Hoogland 2013	23296150	Review, secondary analysis or no original data
Astor 2013	23657143	Association analysis without a prediction model or marker evaluation
Gingell-Littlejohn 2013	23861858	Review, secondary analysis or no original data
Balaz 2013	23912171	Review, secondary analysis or no original data
Losappio 2014	24342976	Outcome was not DGF or graft failure
Malat 2014	24621536	Review, secondary analysis or no original data
Guy 2015	25222017	Association analysis without a prediction model or marker evaluation
Pierson RN 2015	26271536	Review, secondary analysis or no original data
Allen 2016	26361242	Review, secondary analysis or no original data
van den Akker 2015	26451602	Association analysis without a prediction model or marker evaluation
Endre 2016	26950884	Review, secondary analysis or no original data
Malyszko 2016	27496436	Review, secondary analysis or no original data
Wadström 2017	28125449	Review, secondary analysis or no original data
Feng 2017	28213389	Review, secondary analysis or no original data
Hall 2017	28476950	Review, secondary analysis or no original data
McKane 2017	28512161	Review, secondary analysis or no original data
Peters-Sengers 2018b	29380523	Association analysis without a prediction model or marker evaluation
Sandal 2018	29493843	Association analysis without a prediction model or marker evaluation
Garonzik-Wang 2018	30408329	Association analysis without a prediction model or marker evaluation
Mark 2019	30625130	Outcome was not DGF or graft failure
Kim 2019	30861203	Outcome was not DGF or graft failure
Bellos 2019	30954370	Review, secondary analysis or no original data
Konkel 2019	31086705	Association analysis without a prediction model or marker evaluation
Ambrosi 2019	31379196	Association analysis without a prediction model or marker evaluation
Pravisani 2020	32229046	Association analysis without a prediction model or marker evaluation
Friedewald 2020	32632028	Review, secondary analysis or no original data
Mansour 2021	32769629	Association analysis without a prediction model or marker evaluation
Teixeira 2020	33077181	Association analysis without a prediction model or marker evaluation
Ramspek 2021	33340517	Outcome was not DGF or graft failure
Piemonti 2021	33758270	Association analysis without a prediction model or marker evaluation
Singh 2021	34253338	Association analysis without a prediction model or marker evaluation
Korytowska 2021	34437442	Outcome was not DGF or graft failure
van Leeuwen 2021	34448265	Association analysis without a prediction model or marker evaluation
Pinar 2021	34536192	Outcome was not DGF or graft failure
Territo 2022	34657175	Outcome was not DGF or graft failure

Report	PMID	Reason for exclusion
Pravisani 2022	35325442	Not kidney transplantation
Pontrelli 2022	35371047	Association analysis without a prediction model or marker evaluation
Dembowska 2021	35502732	Outcome was not DGF or graft failure
Wang 2022	35844570	Review, secondary analysis or no original data
Jahn 2022	36294469	Outcome was not DGF or graft failure
Ngai 2023	37210325	Association analysis without a prediction model or marker evaluation
Sparding 2023	37238662	Association analysis without a prediction model or marker evaluation
Vanin 2023	37525450	Review, secondary analysis or no original data
Sikosana 2024	37726883	Review, secondary analysis or no original data
Tan 2024	38345067	Association analysis without a prediction model or marker evaluation
Masset 2024	38460985	Review, secondary analysis or no original data
Parajuli 2024	39158946	Association analysis without a prediction model or marker evaluation
Yang 2025	39486235	Review, secondary analysis or no original data
Zhu 2025	40304354	Association analysis without a prediction model or marker evaluation
Buckenmayer 2026	41468499	Association analysis without a prediction model or marker evaluation
Luo 2026	41520687	Animal or in-vitro study
Genç 2026	41772874	Association analysis without a prediction model or marker evaluation
Altamimi 2026	41935146	Outcome was not DGF or graft failure
Momaya 2026	42175357	Association analysis without a prediction model or marker evaluation

72 reports. Records excluded at title and abstract are available from the corresponding author.
